# A novel aggregation framework based on complex n,m-rung orthopair fuzzy aczel-alsina operators for renewable energy decision-making

**DOI:** 10.1038/s41598-025-22119-7

**Published:** 2025-10-31

**Authors:** Ibtesam Alshammari

**Affiliations:** Department of Mathematics, College of Sciences, University of Hafr Al Batin, Hafr Al Batin, Saudi Arabia

**Keywords:** Complex n,m-rung, Orthopair fuzzy sets, Aczé l-Alsina aggregation methods, Uncertainty management, Renewable energy selection, Engineering, Mathematics and computing

## Abstract

This paper develops an advanced decision-making framework using complex n,m-rung orthopair fuzzy (Cn,m-ROF) sets combined with aczel-alsina aggregation operations to effectively manage uncertainty and ambiguity in multiple attribute decision-making (MADM). Two novel aggregation operators—Cn,m-ROFAAWA (weighted average) and Cn,m-ROFAAWG (weighted geometric)—are formulated and examined for their theoretical properties, such as boundedness, idempotency, and monotonicity. The framework is demonstrated through a renewable energy selection case study, where numerical results indicate that Wind Energy consistently ranks highest across varying parameter settings, highlighting the reliability and stability of the proposed approach. Comparative evaluations reveal that the suggested operators outperform existing methods in distinguishing among alternatives and enhancing decision precision. Analysis of parameter influence shows that while larger parameter values increase alternative scores, the optimal choice remains unchanged, confirming isotonicity. Sensitivity assessments further indicate that smaller parameter values improve differentiation among alternatives, ensuring practical applicability. The study underscores the effectiveness of integrating complex n,m-rung orthopair fuzziness with aczel-alsina operations for MADM, and suggests future extensions to other fuzzy information frameworks to enhance applicability and flexibility.

## Introduction

Multi-attribute decision-making is a fundamental approach used in decision analysis for selecting the best alternative among several options based on multiple criteria. This technique is pivotal in fields such as management, engineering, finance, healthcare, and environmental science, where decision-makers often face complex situations involving various attributes and conflicting goals. The main challenge in MADM is to make rational decisions that accommodate uncertainty, imprecision, and subjective judgments. To address these challenges, fuzzy set theory and its extensions have been widely adopted to model and process uncertain and vague information effectively. Fuzzy set (FS) theory, introduced by Zadeh^1^, provides a mathematical framework for handling uncertainty and imprecision by allowing partial membership of elements within a set. Unlike classical sets, where an element either belongs or does not belong to a set, fuzzy sets permit a gradual membership, which is defined by a membership function ranging between 0 and 1. This makes FS particularly suitable for real-world decision-making scenarios, where exact data and crisp boundaries are often unavailable. Fuzzy set theory has found broad applications in MCDM, mathematical modeling, and engineering analysis. In recent years, significant progress has been made in extending fuzzy set theory to more advanced frameworks capable of handling higher-order uncertainty and complex decision-making scenarios. Özlü and Karaaslan^2^ introduced correlation coefficients for T-spherical type-2 hesitant fuzzy sets and demonstrated their effectiveness in clustering applications. Building on this line of research, Özlü^3^ proposed vector similarity measures for picture type-2 hesitant fuzzy sets to enhance multi-criteria decision-making processes, while Özlü^4^ developed generalized Dice measures within the neutrosophic type-2 hesitant fuzzy environment, further broadening the applicability of these models. Complementing these developments, Alqahtani et al.^5^ explored novel fuzzy Ostrowski integral inequalities for convex fuzzy-valued mappings, offering new mathematical tools beyond traditional Sugeno integrals. More recently, Musa et al.^6^ advanced the field by formulating fuzzy N-bipolar soft sets for multi-criteria decision-making, thereby addressing decision contexts that involve bipolarity and complex soft information. To extend the concept of fuzzy sets further, intuitionistic fuzzy sets (IFSs) were presented by Atanassov^7^ as a way to express not just the degree of membership but also the degree of non-membership. An IFS is characterized by a membership function, a non-membership function, and a hesitation margin, which represents the degree of uncertainty or indeterminacy regarding the membership of an element. The combination of these three components enables decision-makers to model more nuanced and realistic scenarios where information is incomplete or uncertain. Pythagorean fuzzy sets (PFSs) were proposed by Yager^8^ as an extension to IFS to handle situations with higher degrees of uncertainty. In PFS, every element is characterized by membership and non-membership degrees whose squared sum does not exceed one. This additional flexibility allows for a broader range of modeling scenarios where the hesitation margin is incorporated into the decision-making process, providing a more comprehensive understanding of complex problems. The q-rung orthopair fuzzy sets (q-ROFSs) are a sophisticated enhancement of fuzzy theory, expanding upon the principles of intuitionistic and Pythagorean fuzzy sets as proposed by Yager^9^. These sets are defined by membership and non-membership functions, together with a parameter *q* that regulates the degree of orthogonality between them. The parameter *q* can vary between 1 and $\infty$, enabling decision-makers to better capture the uncertainty and complexity intrinsic to practical problems. The flexibility of q-ROFSs has made them valuable tools for MADM, allowing for more refined evaluations of alternatives under complex criteria. Fermatean fuzzy sets (FFSs) mark a notable advancement in fuzzy set theory, offering a distinctive approach to handling uncertainty, as presented by Senapati and Yager^10^. FFSs provide a way to represent decision-maker preferences through different types of aggregation operations, adding an extra layer of flexibility in defining how membership and non-membership values are combined. This approach is particularly useful when dealing with decision problems that require complex aggregation strategies for assessing various criteria or when the problem needs to incorporate various levels of confidence and granularity in decision-making. Researchers have actively advanced the theory and applications of fuzzy set environments to address complex decision-making challenges. Fahmi et al.^11^ introduced triangular intuitionistic fuzzy Frank aggregation for renewable energy project selection, while subsequent work Fahmi et al.^12^ proposed circular intuitionistic fuzzy Hamacher aggregation operators to strengthen MADM. In another study, group decision-making was explored through cubic Fermatean fuzzy environments using the Einstein fuzzy weighted geometric operator^13^. Complementing these developments, Özlü and Aktaş^14^ presented correlation coefficients of *r*, *s*, *t*-spherical hesitant fuzzy sets and applied them to MADM problems via clustering algorithms and the TOPSIS method. The n,m-rung orthopair fuzzy set (n,m-ROFS) is an advanced concept that extends the idea of traditional fuzzy and intuitionistic fuzzy sets, as developed by Ibrahim and Alshammari^15^. It incorporates the benefits of both *n*-rung and *m*-rung fuzzy sets, creating a structure where the membership and non-membership values are evaluated at different levels of granularity. The parameters *n* and *m* represent the rungs or levels of the membership and non-membership values, respectively. This set is highly flexible and can model situations with varying degrees of uncertainty and complexity. In practical MADM applications, n,m-ROFSs allow for more precise assessments of alternatives and better handling of decision problems with complex attributes. To elaborate on this argument more comprehensively, let us examine an example where the membership and non-membership degrees are given as (0.83, 0.86). In this context, it becomes clear that the inequality $0.83^q + 0.86^q > 1$ holds true for values of $q \le 4$. This situation underscores a critical limitation: when a uniform exponent *q* is applied to both degrees, the resulting sum exceeds the desired threshold, thereby violating the required constraints. To address this issue, we can introduce asymmetry by employing distinct exponents *n* and *m* for the membership and non-membership degrees, respectively. By choosing appropriate parameters *n* and *m*, the requirement $0.83^n + 0.86^m < 1$ can be satisfied. For example, this condition is met when $n > 4$ and $m = 4$, or alternatively, when $n = 4$ and $m > 4$. These scenarios demonstrate how the strategic use of non-symmetric exponents can effectively ensure that the sum of the powered degrees remains within the acceptable bounds. The integration of these various fuzzy set theories into MADM methodologies enhances the capability to process and aggregate information effectively. Each extension—whether it be IFS, PFS, q-ROFS, FFS, or n,m-ROFS—provides unique features that help decision-makers handle different degrees of uncertainty and complexity. By employing these fuzzy set frameworks in combination with advanced aggregation operators, MADM techniques can achieve a higher level of precision and adaptability, ultimately leading to better-informed decision-making in complex environments.

In recent advancements in MADM, it has become clear that traditional FS theory, IFS, and their extended forms, such as n,m-ROFSs, effectively handle the vagueness and ambiguity inherent in data but fall short in addressing challenges like the absence of historical information and sensitivity to data. These limitations often result in inadequate modeling of dynamic and complex decision-making environments where both periodicity and uncertainty must be accounted for simultaneously. To address these issues, complex fuzzy (CF) sets have emerged by Ramot et al.^16^ as a more powerful tool capable of capturing not just the membership and non-membership of elements but also incorporating the degree of uncertainty through complex-valued functions. Fahmi et al.^17^ examined global economic dynamics by employing the complex cubic fuzzy TODIM method in the context of the Afghan–America war. In another contribution, Özlü^18^ developed bipolar-valued complex hesitant fuzzy Dombi aggregation operators to address multi-criteria decision-making problems. Furthermore, Fahmi et al.^19^ introduced a novel framework for group decision-making grounded in generalized bipolar neutrosophic sets. Complex IF (CIF) sets, as stated by Alkouri and Salleh^20^, build upon IFSs by incorporating complex numbers to represent membership, non-membership, and hesitation, thereby improving the model’s capacity to capture complex relationships within data. Ullah et al.^21^ displayed a complex PF (CPF) sets as an advanced extension of PFSs, integrating the concept of complex numbers to represent membership and non-membership degrees in a more nuanced way. Traditional PFSs expand upon IFSs by allowing the membership and non-membership values to satisfy the condition that their squares sum to a value no greater than one, thus capturing uncertainty and hesitation in decision-making processes. However, CF sets take this further by incorporating complex numbers, which enables the modeling of more sophisticated and detailed relationships within data. Liu et al.^22^ introduced complex q-ROF (Cq-ROF) sets as an advanced extension of fuzzy set theory, expanding upon the concept of q-ROFSs initially proposed by Yager. By incorporating complex numbers into this framework, Cq-ROFS offers a more detailed representation of membership, non-membership, and hesitation, thereby improving the modeling of uncertainty in decision-making processes. Complex FF (CFF) sets, created by Chinnadurai et al.^23^, represent an advanced extension of FFSs, incorporating complex numbers to enhance their expressive capabilities. Complex n,m-ROF (Cn,m-ROF) sets, offered by Ibrahim^24^, provided an advanced extension of n,m-ROFSs, incorporating complex numbers to further enhance their modeling capabilities. By integrating complex numbers into the n,m-rung orthopair fuzzy framework, Cn,m-ROF sets enable the representation of both real and imaginary components in the membership and non-membership values. This development enables for a more sophisticated and refined modeling of data, especially useful in scenarios involving phase-based or oscillatory behavior that traditional real-valued fuzzy sets cannot adequately represent. Owing to its distinctive framework, this approach facilitates a more nuanced and sensitive representation of continuous environments within the context of MADM methodology. As a case in point, envision a decision-maker assigning the complex 5,2-ROF value $(0.85 e^{2 \pi 0.85i}, 0.73 e^{2 \pi 0.73i})$. This value poses a challenge for existing models such as CIFS, CPFS, CFFS, or Cq-ROFS, as these frameworks fail to accommodate it. Specifically, in these models, the condition $0.85^q + 0.73^q \le 1$ is violated for $q \le 3$, underscoring their limitations in handling such scenarios. By leveraging these complex fuzzy set theories, decision-makers can better handle the multidimensional and dynamic nature of real-world problems, providing a more accurate and adaptable approach to MADM in the face of data ambiguity, periodicity, and uncertainty.

Aczél and Alsina^25^ offered a novel class of operations in the context of fuzzy logic, known as Aczél-Alsina (AA) t-norms. These operations belong to the family of triangular norms (t-norms), which are binary functions widely used for modeling the intersection of fuzzy sets in decision-making and information aggregation processes. The AA t-norm is characterized by its parametric structure, offering remarkable flexibility in controlling the aggregation process. Senapati et al.^26^ outlined the intuitionistic fuzzy aggregation operators derived from AA operations, highlighting their benefits when applied to real-life problem-solving scenarios. Hussain et al.^27^ described that similarly, Pythagorean fuzzy AA operators enhance this versatility by relaxing the constraint on membership and non-membership degrees, facilitating more expressive representations of uncertainty. Fermatean fuzzy AA operators, as described by Haq et al.^28^, take this concept further by extending the square sum condition, which allows for the aggregation of higher-dimensional data, thus enabling more complex and detailed representations of uncertainty. The q-ROF AA, and p,q-ROF AA operators, represent a significant generalization, accommodating even broader ranges of uncertainty through increased flexibility in defining orthopair structures, as discussed in^29–31^. In the realm of complex numbers, complex intuitionistic fuzzy AA operators established by Mahmood et al.^32^ as a new dimension of decision-making, adeptly handling problems where both amplitude and phase information are crucial. Extending this to Pythagorean fuzzy structures as outlined by Jin et al.^33^, Fermatean fuzzy structures by Chen et al.^34^, and q-rung orthopair structures by Ali and Naeem^35^ with in the complex domain offers comprehensive aggregation methods that seamlessly merge the interpretability of fuzzy logic with the computational capabilities of complex analysis. These advanced operators form a robust toolkit for tackling intricate problems across various fields, fostering a connection between recent theoretical developments and practical applications in uncertainty modeling and decision sciences.

### Motivation and research gap

The growing demand for robust MADM frameworks has stimulated extensive research into fuzzy set extensions. Traditional models such as intuitionistic fuzzy sets, Pythagorean fuzzy sets, and q-rung orthopair fuzzy sets have enhanced the ability to handle uncertainty; however, they still impose symmetrical constraints on membership and non-membership values. These restrictions limit their adaptability in situations where decision-makers face bipolar, heterogeneous, and multi-layered uncertainty. For example, in renewable energy evaluation or complex resource allocation, existing models may not capture the differing degrees of confidence associated with membership and non-membership judgments. This limitation highlights the theoretical and practical gap in current MADM techniques: the absence of a generalized fuzzy model capable of accommodating distinct rung levels for membership and non-membership, while preserving mathematical soundness.

To address this gap, we raise the central research question of this study: How can a more generalized fuzzy set framework be constructed to enhance aggregation mechanisms and improve MADM under diverse uncertainty conditions? Motivated by this question, we introduce Cn,m-rung orthopair fuzzy sets, a novel extension that unifies the strengths of Cn-rung and Cm-rung fuzzy models. Unlike existing approaches, Cn,m-ROFSs allow membership and non-membership degrees to be defined at independent rungs (n and m), thereby providing an enriched structure for modeling complex information.

The novelty of this work lies in several key contributions. First, we formally establish the operational behavior of Aczél-Alsina t-norms and t-conorms within the Cn,m-ROFS framework, ensuring strong theoretical foundations supported by illustrative examples. Second, we design and analyze novel aggregation operators—namely, the Cn,m-ROFAAWA and Cn,m-ROFAAWAG operators—which exploit the flexibility of the new set structure to generate more reliable decision outcomes. Third, we validate these operators through a real-world application to renewable energy selection, demonstrating their effectiveness in addressing practical MADM challenges. Finally, we conduct a parameter influence analysis and a comparative evaluation with existing methods, confirming the robustness, superiority, and broader applicability of the suggested techniques. Through these contributions, the present study not only fills the identified methodological gap but also extends the boundaries of fuzzy decision-making by providing a more versatile framework that strengthens both theoretical development and practical implementation.

The structure of this manuscript is organized as follows: Section 1 provides a comprehensive review of the historical background and related research, establishing the foundation of the study. Section 2 introduces essential preliminary concepts, including Cn,m-ROFS, Cq-ROFS, CFFS, CPFS, CIFS, along with the fundamental operations of Cn,m-ROFS. Section 3 presents the Aczél–Alsina operational laws specifically designed for Cn,m-ROFNs, forming the core of the mathematical framework. Section 4 extends these laws to construct Aczél–Alsina-based weighted average and weighted geometric aggregation operators for Cn,m-ROFS data. In Section 5, a MADM method is developed within the Cn,m-ROFS environment, and its practical effectiveness is demonstrated through real-world examples. Section 6 offers a comparative analysis, emphasizing the advantages of the suggested method over existing MADM approaches. Section 7 evaluates the sensitivity of the aggregation operators under varying conditions and discusses the limitations of the framework. Finally, Section 8 concludes the paper by summarizing the key findings and contributions, and by highlighting potential directions for future research.

## Preliminaries

This section establishes the foundational preliminary concepts necessary for the subsequent developments.

### Definition 2.1

Consider a universal set $\mathscr {U}$, and define $\mathscr {S}$ as

$$\begin{aligned} \mathscr {S}=\left\{ (\mathscr {E}, \mathscr {P}_{\mathscr {S}}(\mathscr {E}), \mathscr {R}_{\mathscr {S}}(\mathscr {E})): \mathscr {E} \in \mathscr {U}\right\} , \end{aligned}$$

where $\mathscr {P}_{\mathscr {S}}: \mathscr {U} \rightarrow \left\{ \omega _1: \omega _1 \in \mathscr {S},\left| \omega _1\right| \le 1\right\}$    and    $\mathscr {R}_{\mathscr {S}}: \mathscr {U} \rightarrow \left\{ \omega _2: \omega _2 \in \mathscr {S},\left| \omega _2\right| \le 1\right\}$. These mappings satisfy the following:

$$\begin{aligned} \mathscr {P}_{\mathscr {S}}(\mathscr {E})=\omega _1=\alpha _1+i \beta _1 \,\, \text {and}\,\, \mathscr {R}_{\mathscr {S}}(\mathscr {E})=\omega _2=\alpha _2+i \beta _2 , \end{aligned}$$

such that

$$\begin{aligned} 0 \le \left| \omega _1\right| ^n+\left| \omega _2\right| ^m \le 1, \end{aligned}$$

or alternatively,

$$\begin{aligned} \mathscr {P}_{\mathscr {S}}(\mathscr {E})=\mathscr {A}_{\mathscr {S}}(\mathscr {E}) \cdot e^{i .2 \pi \mathscr {D}_{\mathscr {A}_{\mathscr {S}}}(\mathscr {E})} \,\,\, and \,\,\, \mathscr {R}_{\mathscr {S}}(\mathscr {E})=\mathscr {B}_{\mathscr {S}}(\mathscr {E}) \cdot e^{i .2 \pi \mathscr {D}_{\mathscr {B}_{\mathscr {S}}}(\mathscr {E})}, \end{aligned}$$

where the following constraints hold:

$$\begin{aligned} 0 \le \mathscr {A}_{\mathscr {S}}^n(\mathscr {E})+\mathscr {B}_{\mathscr {S}}^m(\mathscr {E}) \le 1 \,\,\text {and}\,\, 0 \le \mathscr {D}_{\mathscr {A}_{\mathscr {S}}}^n(\mathscr {E})+\mathscr {D}_{\mathscr {B}_{\mathscr {S}}}^m(\mathscr {E}) \le 1 . \end{aligned}$$

Here, $\mathscr {A}_{\mathscr {S}}, \mathscr {D}_{\mathscr {A}_{\mathscr {S}}},\mathscr {B}_{\mathscr {S}}, \mathscr {D}_{\mathscr {B}_{\mathscr {S}}}\in [0, 1]$, and $i=\sqrt{-1}$. Based on these definitions, $\mathscr {S}$ is classified as a:

1. CIFS if $$n=m=1$$^20^.
2. CPFS if $$n=m=2$$^21^.
3. CFFS if $$n=m=3$$^23^.
4. Cq-ROFS if $n=m=q$^22^.
5. Cn,m-ROFS if $n\ne m$^24^.

**Fig. 1:**
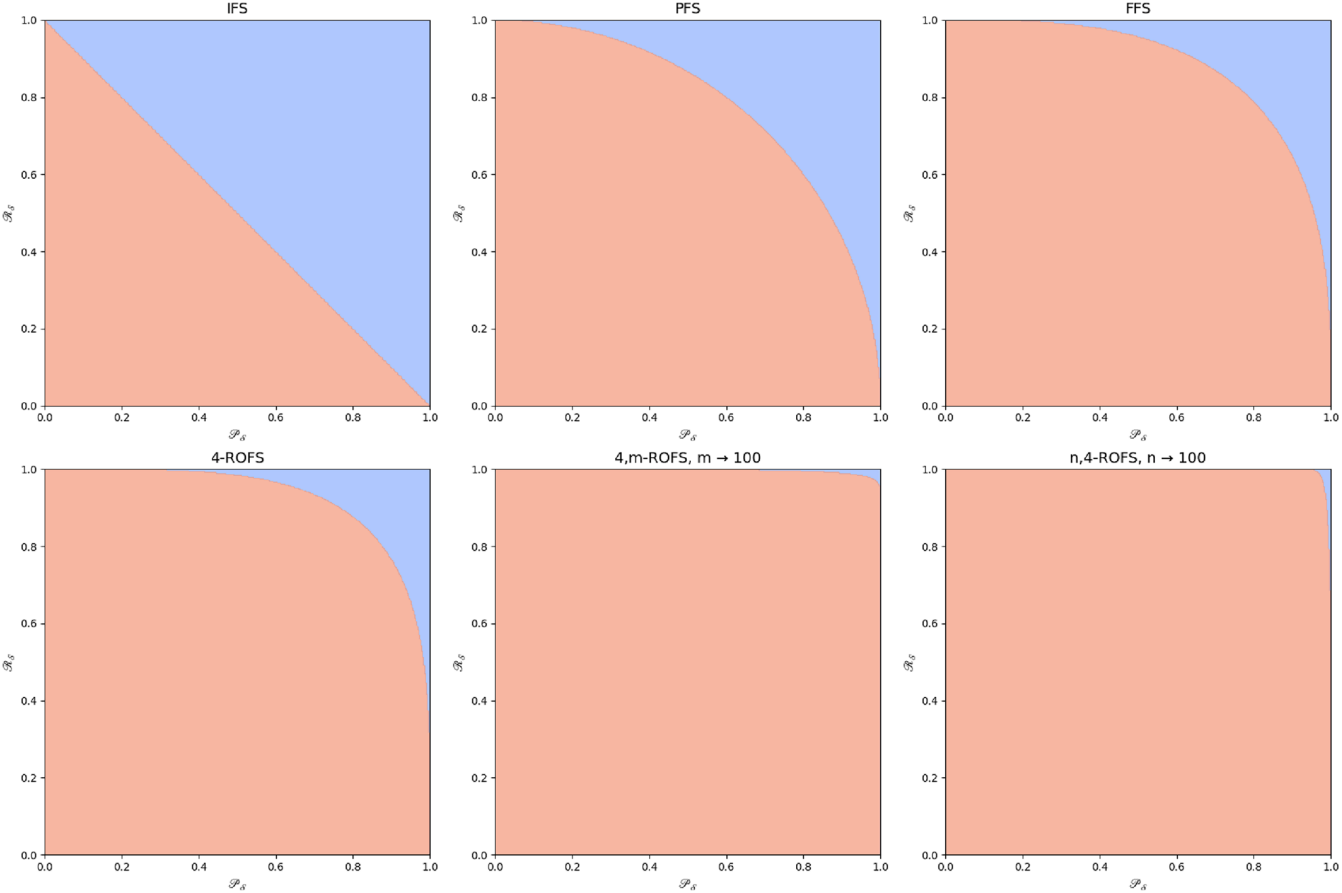
Comparison of fundamental components across different fuzzy set frameworks.

Figure 1 highlights the differences in limitations across various fuzzy set models.

### Definition 2.2

^24^ Let $\mathscr {S}_1\!=\!(\mathscr {A}_{\mathscr {S}_1}\! \cdot\! e^{i.2 \pi \mathscr {D}_{\mathscr {A}_{\mathscr {S}_1}}}\!, \mathscr {B}_{\mathscr {S}_1} \!\cdot\! e^{i \cdot 2 \pi \mathscr {D}_{\mathscr {B}_{\mathscr {S}_1}}}\!)$ and    $\mathscr {S}_2\!=\!(\mathscr {A}_{\mathscr {S}_2}\! \cdot\! e^{i.2 \pi \mathscr {D}_{\mathscr {A}_{\mathscr {S}_2}}}, \mathscr {B}_{\mathscr {S}_2}\! \cdot \!e^{i.2 \pi \mathscr {D}_{\mathscr {B}_{\mathscr {S}_2}}}\!)$ be two Cn,m-ROF numbers (Cn,m-ROFNs) and consider $\mathscr {L}$ as a positive real number. Hence,

1. $\mathscr {S}_1 \subseteq \mathscr {S}_2$ if and only if:
  $$\mathscr {A}_{\mathscr {S}_1} \le \mathscr {A}_{\mathscr {S}_2}, \quad \mathscr {B}_{\mathscr {S}_1} \ge \mathscr {B}_{\mathscr {S}_2}, \quad \mathscr {D}_{\mathscr {A}_{\mathscr {S}_1}} \le \mathscr {D}_{\mathscr {A}_{\mathscr {S}_2}}, \quad \quad \text {and} \quad \quad \mathscr {D}_{\mathscr {B}_{\mathscr {S}_1}} \ge \mathscr {D}_{\mathscr {B}_{\mathscr {S}_2}}.$$
2. $$\mathscr {S}_1 \le \mathscr {S}_2$$ if $$\mathscr {S}_1 \subseteq \mathscr {S}_2$$.
3. $\mathscr {S}_1=\mathscr {S}_2$ if and only if:
  $$\mathscr {A}_{\mathscr {S}_1} = \mathscr {A}_{\mathscr {S}_2}, \quad \mathscr {B}_{\mathscr {S}_1} = \mathscr {B}_{\mathscr {S}_2}, \quad \mathscr {D}_{\mathscr {A}_{\mathscr {S}_1}} = \mathscr {D}_{\mathscr {A}_{\mathscr {S}_2}}, \quad \quad \text {and} \quad \quad \mathscr {D}_{\mathscr {B}_{\mathscr {S}_1}} = \mathscr {D}_{\mathscr {B}_{\mathscr {S}_2}}.$$
4. The complement of $\mathscr {S}_1$ is given by:
  $$\mathscr {S}_1^c=(\mathscr {B}_{\mathscr {S}_1}^{\frac{m}{n}} \cdot e^{i .2 \pi \mathscr {D}_{\mathscr {B}_{\mathscr {S}_1}}^{\frac{m}{n}}}, \mathscr {A}_{\mathscr {S}_1}^{\frac{n}{m}} \cdot e^{i \cdot 2 \pi \mathscr {D}_{\mathscr {A}_{\mathscr {S}_1}}^{\frac{n}{m}}}).$$
5. The addition of $\mathscr {S}_{1}$ and $\mathscr {S}_{2}$ is defined as: $\mathscr {S}_{1} \oplus \mathscr {S}_{2}=((\mathscr {A}_{\mathscr {S}_1}^{n}+\mathscr {A}_{\mathscr {S}_2}^{n}-\mathscr {A}_{\mathscr {S}_1}^{n} \mathscr {A}_{\mathscr {S}_2}^{n})^{\frac{1}{n}} \cdot e^{i \cdot 2 \pi \cdot (\mathscr {D}_{\mathscr {A}_{\mathscr {S}_1}}^{n}+\mathscr {D}_{\mathscr {A}_{\mathscr {S}_2}}^{n}-\mathscr {D}_{\mathscr {A}_{\mathscr {S}_1}}^{n} \mathscr {D}_{\mathscr {A}_{\mathscr {S}_2}}^{n})^{\frac{1}{n}}},$ $(\mathscr {B}_{\mathscr {S}_1} \mathscr {B}_{\mathscr {S}_2}) \cdot e^{i \cdot 2 \pi (\mathscr {D}_{\mathscr {B}_{\mathscr {S}_1}} \mathscr {D}_{\mathscr {B}_{\mathscr {S}_2}})}).$
6. The multiplication of $\mathscr {S}_{1}$ and $\mathscr {S}_{2}$ is defined as: $\mathscr {S}_{1} \otimes \mathscr {S}_{2}=((\mathscr {A}_{\mathscr {S}_1} \mathscr {A}_{\mathscr {S}_2}) \cdot e^{i \cdot 2 \pi (\mathscr {D}_{\mathscr {A}_{\mathscr {S}_1}} \mathscr {D}_{\mathscr {A}_{\mathscr {S}_2}})},$ $(\mathscr {B}_{\mathscr {S}_1}^{m}+\mathscr {B}_{\mathscr {S}_2}^{m}-\mathscr {B}_{\mathscr {S}_1}^{m} \mathscr {B}_{\mathscr {S}_2}^{m})^{\frac{1}{m}} \cdot e^{i \cdot 2 \pi \cdot (\mathscr {D}_{\mathscr {B}_{\mathscr {S}_1}}^{m}+\mathscr {D}_{\mathscr {B}_{\mathscr {S}_2}}^{m}-\mathscr {D}_{\mathscr {B}_{\mathscr {S}_1}}^{m} \mathscr {D}_{\mathscr {B}_{\mathscr {S}_2}}^{m})^{\frac{1}{m}}}).$
7. Scalar multiplication by $\mathscr {L}$ is given as:
  $$\mathscr {L} \mathscr {S}_{j}=((1-(1-\mathscr {A}_{\mathscr {S}_j}^{n})^{\mathscr {L}})^{\frac{1}{n}} \cdot e^{i \cdot 2 \pi (1-(1-\mathscr {D}_{\mathscr {A}_{\mathscr {S}_j}}^{n})^{\mathscr {L}})^{\frac{1}{n}}}, \mathscr {B}_{\mathscr {S}_j}^{\mathscr {L}} \cdot e^{i \cdot 2 \pi \mathscr {D}_{\mathscr {B}_{\mathscr {S}_j}}^{\mathscr {L}}}), \quad j=1,2.$$
8. Exponentiation by by $\mathscr {L}$ is defined as:
  $$\mathscr {S}_{j}^{\mathscr {L}}=(\mathscr {A}_{\mathscr {S}_j}^{\mathscr {L}} \cdot e^{i \cdot 2 \pi \mathscr {D}_{\mathscr {A}_{\mathscr {S}_j}}^{\mathscr {L}}},(1-(1-\mathscr {B}_{\mathscr {S}_j}^{m})^{\mathscr {L}})^{\frac{1}{m}} \cdot e^{i \cdot 2 \pi (1-(1-\mathscr {D}_{\mathscr {B}_{\mathscr {S}_j}}^{m})^{\mathscr {L}})^{\frac{1}{m}}}), \quad j=1,2.$$

### Definition 2.3

^24^ Let $\mathscr {S} = \left( \mathscr {A}_{\mathscr {S}} \cdot e^{i \cdot 2 \pi \mathscr {D}_{\mathscr {A}_{\mathscr {S}}}}, \mathscr {B}_{\mathscr {S}} \cdot e^{i \cdot 2 \pi \mathscr {D}_{\mathscr {B}_{\mathscr {S}}}} \right)$ represent a Cn,m-ROFN. The functions $\dot{s}(\mathscr {S})$ and $\dot{a}(\mathscr {S})$, representing the score and accuracy of $\mathscr {S}$ respectively, are formally introduced as:

$$\begin{aligned} \dot{s}(\mathscr {S})&= \frac{1}{2} \left[ \left( \mathscr {A}_{\mathscr {S}}^n - \mathscr {B}_{\mathscr {S}}^m \right) + \left( \mathscr {D}_{\mathscr {A}_{\mathscr {S}}}^n - \mathscr {D}_{\mathscr {B}_{\mathscr {S}}}^m \right) \right] , \\ \dot{a}(\mathscr {S})&= \frac{1}{2} \left[ \left( \mathscr {A}_{\mathscr {S}}^n + \mathscr {B}_{\mathscr {S}}^m \right) + \left( \mathscr {D}_{\mathscr {A}_{\mathscr {S}}}^n + \mathscr {D}_{\mathscr {B}_{\mathscr {S}}}^m \right) \right] . \end{aligned}$$

### Definition 2.4

^24^ Let $$\mathscr {S}_1=(\mathscr {A}_{\mathscr {S}_1} \cdot e^{i.2 \pi \mathscr {D}_{\mathscr {A}_{\mathscr {S}_1}}}, \mathscr {B}_{\mathscr {S}_1} \cdot e^{i \cdot 2 \pi \mathscr {D}_{\mathscr {B}_{\mathscr {S}_1}}})$$ and

$\mathscr {S}_2=(\mathscr {A}_{\mathscr {S}_2} \cdot e^{i.2 \pi \mathscr {D}_{\mathscr {A}_{\mathscr {S}_2}}}, \mathscr {B}_{\mathscr {S}_2} \cdot e^{i \cdot 2 \pi \mathscr {D}_{\mathscr {B}_{\mathscr {S}_2}}})$ represent two Cn,m-ROFNs. The comparison is formulated as follows:

1. $$\mathscr {S}_{1}\prec \mathscr {S}_{2}$$ if $$\dot{s}(\mathscr {S}_{1})< \dot{s}(\mathscr {S}_{2})$$.
2. $$\mathscr {S}_{1}\succ \mathscr {S}_{2}$$ if $$\dot{s}(\mathscr {S}_{1}) > \dot{s}(\mathscr {S}_{2})$$.
3. When $\dot{s}(\mathscr {S}_{1}) = \dot{s}(\mathscr {S}_{2})$, the comparison is refined using the accuracy function:
  $$\mathscr {S}_1 \; {\left\{ \begin{array}{ll} \prec \mathscr {S}_2, & \text {if } \dot{a}(\mathscr {S}_1) < \dot{a}(\mathscr {S}_2). \\ \succ \mathscr {S}_2, & \text {if } \dot{a}(\mathscr {S}_1) > \dot{a}(\mathscr {S}_2). \\ \approx \mathscr {S}_2, & \text {if } \dot{a}(\mathscr {S}_1) = \dot{a}(\mathscr {S}_2). \end{array}\right. }$$

## **Aczél-Alsina operational laws for Cn,m-ROFSs**

This section introduces the aczel-alsina operational principles specifically tailored for Cn,m-ROFNs, providing the mathematical foundation for developing the proposed aggregation operators and supporting subsequent decision-making analyses.

### Definition 3.1

Let $$\mathscr {S}=\left( \mathscr {A}_{\mathscr {S}} e^{2 \pi i\mathscr {D}_{\mathscr {A}_{\mathscr {S}}}}, \mathscr {B}_{\mathscr {S}} e^{2 \pi i\mathscr {D}_{\mathscr {B}_{\mathscr {S}}}}\right) ,$$

$\mathscr {S}_1=$ $\left( \mathscr {A}_{\mathscr {S}_1} e^{2 \pi i\mathscr {D}_{\mathscr {A}_{\mathscr {S}_1}}}, \mathscr {B}_{\mathscr {S}_1} e^{2 \pi i\mathscr {D}_{\mathscr {B}_{\mathscr {S}_1}}}\right)$ and $\mathscr {S}_2=\left( \mathscr {A}_{\mathscr {S}_2} e^{2 \pi i\mathscr {D}_{\mathscr {A}_{\mathscr {S}_2}}}, \mathscr {B}_{\mathscr {S}_2} e^{2 \pi i\mathscr {D}_{\mathscr {B}_{\mathscr {S}_2}}}\right)$ represent three Cn,m-ROFNs, $\mathscr {I} \ge 1$ and $\mathscr {L}>0$. Then:

1 $$\begin{aligned} \mathscr {S}_1 \boxplus \mathscr {S}_2 =\left( \begin{array}{c} \root n \of {1-\textsf{e}^{-\left( \left( -ln \left( 1-\mathscr {A}_{\mathscr {S}_1}^{n}\right) \right) ^\mathscr {I}+\left( -ln \left( 1-\mathscr {A}_{\mathscr {S}_2}^{n}\right) \right) ^\mathscr {I}\right) ^{\frac{1}{\mathscr {I}}}}}\\ \textsf{e}^{2 i \pi \root n \of {1-\textsf{e}^{-\left( \left( -ln \left( 1-\mathscr {D}_{\mathscr {A}_{\mathscr {S}_1}}^{n}\right) \right) ^\mathscr {I}+\left( -ln \left( 1-\mathscr {D}_{\mathscr {A}_{\mathscr {S}_2}}^{n}\right) \right) ^\mathscr {I}\right) ^{\frac{1}{\mathscr {I}}}}}},\\ \root m \of {\textsf{e}^{-\left( \left( -ln \mathscr {B}_{\mathscr {S}_1}^{m}\right) ^\mathscr {I}+\left( -ln \mathscr {B}_{\mathscr {S}_2}^{m}\right) ^\mathscr {I}\right) ^{\frac{1}{\mathscr {I}}}}}\\ \textsf{e}^{2 i \pi \root m \of {\textsf{e}^{-\left( \left( -ln \mathscr {D}_{\mathscr {B}_{\mathscr {S}_1}}^{m}\right) ^\mathscr {I}+\left( -ln \mathscr {D}_{\mathscr {B}_{\mathscr {S}_2}}^{m}\right) ^\mathscr {I}\right) ^{\frac{1}{\mathscr {I}}}}}} \end{array}\right) \end{aligned}$$

2 $$\begin{aligned} \mathscr {S}_1\boxtimes \mathscr {S}_2=\left( \begin{array}{c} \root n \of {\textsf{e}^{-\left( \left( -ln \mathscr {A}_{\mathscr {S}_1}^{n}\right) ^\mathscr {I}+\left( -ln \mathscr {A}_{\mathscr {S}_2}^{n}\right) ^\mathscr {I}\right) ^{\frac{1}{\mathscr {I}}}}}\\ \textsf{e}^{2 i \pi \root n \of {\textsf{e}^{-\left( \left( -ln \mathscr {D}_{\mathscr {A}_{\mathscr {S}_1}}^{n}\right) ^\mathscr {I}+\left( -ln \mathscr {D}_{\mathscr {A}_{\mathscr {S}_2}}^{n}\right) ^\mathscr {I}\right) ^{\frac{1}{\mathscr {I}}}}}},\\ \root m \of {1-\textsf{e}^{-\left( \left( -ln \left( 1-\mathscr {B}_{\mathscr {S}_1}^{m}\right) \right) ^\mathscr {I}+\left( -ln \left( 1-\mathscr {B}_{\mathscr {S}_2}^{m}\right) \right) ^\mathscr {I}\right) ^{\frac{1}{\mathscr {I}}}}}\\ \textsf{e}^{2 i \pi \root m \of {1-\textsf{e}^{-\left( \left( -ln \left( 1-\mathscr {D}_{\mathscr {B}_{\mathscr {S}_1}}^{m}\right) \right) ^\mathscr {I}+\left( -ln \left( 1-\mathscr {D}_{\mathscr {B}_{\mathscr {S}_2}}^{m}\right) \right) ^\mathscr {I}\right) ^{\frac{1}{\mathscr {I}}}}}} \end{array}\right) \end{aligned}$$

3 $$\begin{aligned} \mathscr {L}\mathscr {S} =\left( \begin{array}{c} \root n \of {1-\textsf{e}^{-\left( \mathscr {L}\left( -ln \left( 1-\mathscr {A}_{\mathscr {S}}^{n}\right) \right) ^\mathscr {I}\right) ^{\frac{1}{\mathscr {I}}}}}\\ \textsf{e}^{2 i \pi \root n \of {1-\textsf{e}^{-\left( \mathscr {L}\left( -ln \left( 1-\mathscr {D}_{\mathscr {A}_{\mathscr {S}}}^{n}\right) \right) ^\mathscr {I}\right) ^{\frac{1}{\mathscr {I}}}}}},\\ \root m \of {\textsf{e}^{-\left( \mathscr {L}\left( -ln \mathscr {B}_{\mathscr {S}}^{m}\right) ^\mathscr {I}\right) ^{\frac{1}{\mathscr {I}}}}}\\ \textsf{e}^{2 i \pi \root m \of {\textsf{e}^{-\left( \mathscr {L}\left( -ln \mathscr {D}_{\mathscr {B}_{\mathscr {S}}}^{m}\right) ^\mathscr {I}\right) ^{\frac{1}{\mathscr {I}}}}}} \end{array}\right) \end{aligned}$$

4 $$\begin{aligned} \mathscr {S}^{\mathscr {L}} =\left( \begin{array}{c} \root n \of {\textsf{e}^{-\left( \mathscr {L}\left( -ln \mathscr {A}_{\mathscr {S}}^{n}\right) ^\mathscr {I}\right) ^{\frac{1}{\mathscr {I}}}}}\\ \textsf{e}^{2 i \pi \root n \of {\textsf{e}^{-\left( \mathscr {L}\left( -ln \mathscr {D}_{\mathscr {A}_{\mathscr {S}}}^{n}\right) ^\mathscr {I}\right) ^{\frac{1}{\mathscr {I}}}}}},\\ \root m \of {1-\textsf{e}^{-\left( \mathscr {L}\left( -ln \left( 1-\mathscr {B}_{\mathscr {S}}^{m}\right) \right) ^\mathscr {I}\right) ^{\frac{1}{\mathscr {I}}}}}\\ \textsf{e}^{2 i \pi \root m \of {1-\textsf{e}^{-\left( \mathscr {L}\left( -ln \left( 1-\mathscr {D}_{\mathscr {B}_{\mathscr {S}}}^{m}\right) \right) ^\mathscr {I}\right) ^{\frac{1}{\mathscr {I}}}}}} \end{array}\right) \end{aligned}$$

### Example 3.2

Let us define the three C3,2-ROFNs as follows: $\mathscr {S} = \left( 0.73 e^{2\pi i (0.25)}, 0.69 e^{2\pi i (0.21)}\right) ,$ $\mathscr {S}_1 = \left( 0.55 e^{2\pi i (0.66)}, 0.51 e^{2\pi i (0.62)}\right)$, and $\mathscr {S}_2 = \left( 0.73 e^{2\pi i (0.77)}, 0.77 e^{2\pi i (0.73)}\right)$. For $\mathscr {I} = 4$ and $\mathscr {L} = 5$, we derive the following results:

1. $$\mathscr {S}_1 \boxplus \mathscr {S}_2 =\left( \begin{array}{c} \root 3 \of {1-\textsf{e}^{-\left( \left( -ln \left( 1-\mathscr {A}_{\mathscr {S}_1}^{3}\right) \right) ^4+\left( -ln \left( 1-\mathscr {A}_{\mathscr {S}_2}^{3}\right) \right) ^4\right) ^{1 / 4}}}\\ \textsf{e}^{2 i \pi \root 3 \of {1-\textsf{e}^{-\left( \left( -ln \left( 1-\mathscr {D}_{\mathscr {A}_{\mathscr {S}_1}}^{3}\right) \right) ^4+\left( -ln \left( 1-\mathscr {D}_{\mathscr {A}_{\mathscr {S}_2}}^{3}\right) \right) ^4\right) ^{1 / 4}}},}\\ \sqrt{\textsf{e}^{-\left( \left( -ln \mathscr {B}_{\mathscr {S}_1}^{2}\right) ^4+\left( -ln \mathscr {B}_{\mathscr {S}_2}^{2}\right) ^4\right) ^{1 / 4}}}\\ \textsf{e}^{2 i \pi \sqrt{\textsf{e}^{-\left( \left( -ln \mathscr {D}_{\mathscr {B}_{\mathscr {S}_1}}^{2}\right) ^4+\left( -ln \mathscr {D}_{\mathscr {B}_{\mathscr {S}_2}}^{2}\right) ^4\right) ^{1 / 4}}}} \end{array}\right)$$ $$=\left( \begin{array}{c} \root 3 \of {1-\textsf{e}^{-\left( \left( -ln \left( 1-0.55^{3}\right) \right) ^4+\left( -ln \left( 1-0.73^{3}\right) \right) ^4\right) ^{1 / 4}}}\\ \textsf{e}^{2 i \pi \root 3 \of {1-\textsf{e}^{-\left( \left( -ln \left( 1-0.66^{3}\right) \right) ^4+\left( -ln \left( 1-0.77^{3}\right) \right) ^4\right) ^{1 / 4}}},}\\ \sqrt{\textsf{e}^{-\left( \left( -ln 0.51^{2}\right) ^4+\left( -ln 0.77^{2}\right) ^4\right) ^{1 / 4}}}\\ \textsf{e}^{2 i \pi \sqrt{\textsf{e}^{-\left( \left( -ln 0.62^{2}\right) ^4+\left( -ln 0.73^{2}\right) ^4\right) ^{1 / 4}}}} \end{array}\right) \approx \left( 0.7309 e^{2\pi i (0.7742)}, 0.5081 e^{2\pi i (0.6071)}\right)$$
2. $$\mathscr {S}_1\boxtimes \mathscr {S}_2=\left( \begin{array}{c} \root 3 \of {\textsf{e}^{-\left( \left( -ln \mathscr {A}_{\mathscr {S}_1}^{3}\right) ^4+\left( -ln \mathscr {A}_{\mathscr {S}_2}^{3}\right) ^4\right) ^{1 / 4}}}\\ \textsf{e}^{2 i \pi \root 3 \of {\textsf{e}^{-\left( \left( -ln \mathscr {D}_{\mathscr {A}_{\mathscr {S}_1}}^{3}\right) ^4+\left( -ln \mathscr {D}_{\mathscr {A}_{\mathscr {S}_2}}^{3}\right) ^4\right) ^{1 / 4}}}},\\ \sqrt{1-\textsf{e}^{-\left( \left( -ln \left( 1-\mathscr {B}_{\mathscr {S}_1}^{2}\right) \right) ^4+\left( -ln \left( 1-\mathscr {B}_{\mathscr {S}_2}^{2}\right) \right) ^4\right) ^{1 / 4}}}\\ \textsf{e}^{2 i \pi \sqrt{1-\textsf{e}^{-\left( \left( -ln \left( 1-\mathscr {D}_{\mathscr {B}_{\mathscr {S}_1}}^{2}\right) \right) ^4+\left( -ln \left( 1-\mathscr {D}_{\mathscr {B}_{\mathscr {S}_2}}^{2}\right) \right) ^4\right) ^{1 / 4}}}} \end{array}\right)$$ $$=\left( \begin{array}{c} \root 3 \of {\textsf{e}^{-\left( \left( -ln 0.55^{3}\right) ^4+\left( -ln 0.73^{3}\right) ^4\right) ^{1 / 4}}}\\ \textsf{e}^{2 i \pi \root 3 \of {\textsf{e}^{-\left( \left( -ln 0.66^{3}\right) ^4+\left( -ln 0.77^{3}\right) ^4\right) ^{1 / 4}}}},\\ \sqrt{1-\textsf{e}^{-\left( \left( -ln \left( 1-0.51^{2}\right) \right) ^4+\left( -ln \left( 1-0.77^{2}\right) \right) ^4\right) ^{1 / 4}}}\\ \textsf{e}^{2 i \pi \sqrt{1-\textsf{e}^{-\left( \left( -ln \left( 1-0.62^{2}\right) \right) ^4+\left( -ln \left( 1-0.73^{2}\right) \right) ^4\right) ^{1 / 4}}}} \end{array}\right) \approx \left( 0.5439 e^{2\pi i (0.6499)}, 0.7707 e^{2\pi i (0.7393)}\right)$$
3. $$5\mathscr {S} =\left( \begin{array}{c} \root 3 \of {1-\textsf{e}^{-\left( 5\left( \left( -ln \left( 1-\mathscr {A}_{\mathscr {S}}^{3}\right) \right) ^4\right) \right) ^{1 / 4}}}\\ \textsf{e}^{2 i \pi \root 3 \of {1-\textsf{e}^{-\left( 5\left( \left( -ln \left( 1-\mathscr {D}_{\mathscr {A}_{\mathscr {S}}}^{3}\right) \right) ^4\right) \right) ^{1 / 4}}}},\\ \sqrt{\textsf{e}^{-\left( 5\left( -ln \mathscr {B}_{\mathscr {S}}^{2}\right) ^4\right) ^{1 / 4}}}\\ \textsf{e}^{2 i \pi \sqrt{\textsf{e}^{-\left( 5\left( -ln \mathscr {D}_{\mathscr {B}_{\mathscr {S}}}^{2}\right) ^4\right) ^{1 / 4}}}} \end{array}\right)$$ $$=\left( \begin{array}{c} \root 3 \of {1-\textsf{e}^{-\left( 5\left( \left( -ln \left( 1-0.73^{3}\right) \right) ^4\right) \right) ^{1 / 4}}}\\ \textsf{e}^{2 i \pi \root 3 \of {1-\textsf{e}^{-\left( 5\left( \left( -ln \left( 1-0.25^{3}\right) \right) ^4\right) \right) ^{1 / 4}}}},\\ \sqrt{\textsf{e}^{-\left( 5\left( -ln 0.69^{2}\right) ^4\right) ^{1 / 4}}}\\ \textsf{e}^{2 i \pi \sqrt{\textsf{e}^{-\left( 5\left( -ln 0.21^{2}\right) ^4\right) ^{1 / 4}}}} \end{array}\right) \approx \left( 0.8048 e^{2\pi i (0.2855)}, 0.5741 e^{2\pi i (0.0969)}\right)$$
4. $$\mathscr {S}^{5} =\left( \begin{array}{c} \root 3 \of {\textsf{e}^{-\left( 5\left( -ln \mathscr {A}_{\mathscr {S}}^{3}\right) ^4\right) ^{1 / 4}}}\\ \textsf{e}^{2 i \pi \root 3 \of {\textsf{e}^{-\left( 5\left( -ln \mathscr {D}_{\mathscr {A}_{\mathscr {S}}}^{3}\right) ^4\right) ^{1 / 4}}}},\\ \sqrt{1-\textsf{e}^{-\left( 5\left( \left( -ln \left( 1-\mathscr {B}_{\mathscr {S}}^{2}\right) \right) ^4\right) \right) ^{1 / 4}}}\\ \textsf{e}^{2 i \pi \sqrt{1-\textsf{e}^{-\left( 5\left( \left( -ln \left( 1-\mathscr {D}_{\mathscr {B}_{\mathscr {S}}}^{2}\right) \right) ^4\right) \right) ^{1 / 4}}}} \end{array}\right)$$ $$=\left( \begin{array}{c} \root 3 \of {\textsf{e}^{-\left( 5\left( -ln 0.73^{3}\right) ^4\right) ^{1 / 4}}}\\ \textsf{e}^{2 i \pi \root 3 \of {\textsf{e}^{-\left( 5\left( -ln 0.25^{3}\right) ^4\right) ^{1 / 4}}}},\\ \sqrt{1-\textsf{e}^{-\left( 5\left( \left( -ln \left( 1-0.69^{2}\right) \right) ^4\right) \right) ^{1 / 4}}}\\ \textsf{e}^{2 i \pi \sqrt{1-\textsf{e}^{-\left( 5\left( \left( -ln \left( 1-0.21^{2}\right) \right) ^4\right) \right) ^{1 / 4}}}} \end{array}\right) \approx \left( 0.6246 e^{2\pi i (0.1258)}, 0.7872 e^{2\pi i (0.2554)}\right)$$

### Theorem 3.3

Let $\mathscr {S}_1\!=\!(\mathscr {A}_{\mathscr {S}_1} \!\cdot\! e^{i.2 \pi \mathscr {D}_{\mathscr {A}_{\mathscr {S}_1}}}, \mathscr {B}_{\mathscr {S}_1} \!\cdot\! e^{i \cdot 2 \pi \mathscr {D}_{\mathscr {B}_{\mathscr {S}_1}}})$ and $\mathscr {S}_2\!=\!(\mathscr {A}_{\mathscr {S}_2}\! \cdot\! e^{i.2 \pi \mathscr {D}_{\mathscr {A}_{\mathscr {S}_2}}}, \mathscr {B}_{\mathscr {S}_2}\! \cdot\! e^{i \cdot 2 \pi \mathscr {D}_{\mathscr {B}_{\mathscr {S}_2}}})$ be two Cn,m-ROFNs. Then, their addition $\mathscr {S}_1 \boxplus \mathscr {S}_2$ and multiplication $\mathscr {S}_1 \boxtimes \mathscr {S}_2$ also result in Cn,m-ROFNs.

### Proof

The following identities are satisfied by the Cn,m-ROFNs $\mathscr {S}_1$ and $\mathscr {S}_2$:

$$\begin{aligned} 0 \le \mathscr {A}_{\mathscr {S}_1}^n+\mathscr {B}_{\mathscr {S}_1}^m \le 1, 0 \le \mathscr {D}_{\mathscr {A}_{\mathscr {S}_1}}^n+\mathscr {D}_{\mathscr {B}_{\mathscr {S}_1}}^m \le 1 , \end{aligned}$$

$$\begin{aligned} 0 \le \mathscr {A}_{\mathscr {S}_2}^n+\mathscr {B}_{\mathscr {S}_2}^m \le 1, \text {and} 0 \le \mathscr {D}_{\mathscr {A}_{\mathscr {S}_2}}^n+\mathscr {D}_{\mathscr {B}_{\mathscr {S}_2}}^m \le 1 . \end{aligned}$$

Then, we obtain:

$$\begin{aligned} 0\le 1-\mathscr {A}_{\mathscr {S}_1}^{n}, 0\le 1-\mathscr {A}_{\mathscr {S}_2}^{n}, 0\le \mathscr {B}_{\mathscr {S}_1}^{m} \text {and} 0\le \mathscr {B}_{\mathscr {S}_2}^{m} \end{aligned}$$

which implies that

$$\begin{aligned} 0\le -ln(1-\mathscr {A}_{\mathscr {S}_1}^{n}), 0\le -ln(1-\mathscr {A}_{\mathscr {S}_2}^{n}), 0\le -ln(\mathscr {B}_{\mathscr {S}_1}^{m}) \text {and } 0\le -ln(\mathscr {B}_{\mathscr {S}_2}^{m}), \end{aligned}$$

hence

$$\begin{aligned} \left( -ln \left( 1-\mathscr {A}_{\mathscr {S}_1}^{n}\right) \right) ^\mathscr {I}+\left( -ln \left( 1-\mathscr {A}_{\mathscr {S}_2}^{n}\right) \right) ^\mathscr {I}\ge 0 \end{aligned}$$

and

$$\begin{aligned} \left( -ln \left( \mathscr {B}_{\mathscr {S}_1}^{m}\right) \right) ^\mathscr {I}+\left( -ln \left( \mathscr {B}_{\mathscr {S}_2}^{m}\right) \right) ^\mathscr {I}\ge 0, \end{aligned}$$

therefore

$$\begin{aligned} -\left( \left( -ln \left( 1-\mathscr {A}_{\mathscr {S}_1}^{n}\right) \right) ^\mathscr {I}+\left( -ln \left( 1-\mathscr {A}_{\mathscr {S}_2}^{n}\right) \right) ^\mathscr {I}\right) ^{\frac{1}{\mathscr {I}}}\le 0 \end{aligned}$$

and

$$\begin{aligned} -\left( \left( -ln \left( \mathscr {B}_{\mathscr {S}_1}^{m}\right) \right) ^\mathscr {I}+\left( -ln \left( \mathscr {B}_{\mathscr {S}_2}^{m}\right) \right) ^\mathscr {I}\right) ^{\frac{1}{\mathscr {I}}}\le 0 \end{aligned}$$

which illustrates that

$$\begin{aligned} 1-\textsf{e}^{-\left( \left( -ln \left( 1-\mathscr {A}_{\mathscr {S}_1}^{n}\right) \right) ^\mathscr {I}+\left( -ln \left( 1-\mathscr {A}_{\mathscr {S}_2}^{n}\right) \right) ^\mathscr {I}\right) ^{\frac{1}{\mathscr {I}}}}\ge 0 \end{aligned}$$

implies

$$\begin{aligned} \root n \of {1-\textsf{e}^{-\left( \left( -ln \left( 1-\mathscr {A}_{\mathscr {S}_1}^{n}\right) \right) ^\mathscr {I}+\left( -ln \left( 1-\mathscr {A}_{\mathscr {S}_2}^{n}\right) \right) ^\mathscr {I}\right) ^{\frac{1}{\mathscr {I}}}}}\ge 0 \end{aligned}$$

and

$$\begin{aligned} \textsf{e}^{-\left( \left( -ln \mathscr {B}_{\mathscr {S}_1}^{m}\right) ^\mathscr {I}+\left( -ln \mathscr {B}_{\mathscr {S}_2}^{m}\right) ^\mathscr {I}\right) ^{\frac{1}{\mathscr {I}}}}\ge 0 \end{aligned}$$

implies

$$\begin{aligned} \root m \of {\textsf{e}^{-\left( \left( -ln \mathscr {B}_{\mathscr {S}_1}^{m}\right) ^\mathscr {I}+\left( -ln \mathscr {B}_{\mathscr {S}_2}^{m}\right) ^\mathscr {I}\right) ^{\frac{1}{\mathscr {I}}}}}\ge 0. \end{aligned}$$

Clearly,

$$\begin{aligned} \root n \of {1-\textsf{e}^{-\left( \left( -ln \left( 1-\mathscr {A}_{\mathscr {S}_1}^{n}\right) \right) ^\mathscr {I}+\left( -ln \left( 1-\mathscr {A}_{\mathscr {S}_2}^{n}\right) \right) ^\mathscr {I}\right) ^{\frac{1}{\mathscr {I}}}}} \le 1 \end{aligned}$$

and

$$\begin{aligned} \root m \of {\textsf{e}^{-\left( \left( -ln \mathscr {B}_{\mathscr {S}_1}^{m}\right) ^\mathscr {I}+\left( -ln \mathscr {B}_{\mathscr {S}_2}^{m}\right) ^\mathscr {I}\right) ^{\frac{1}{\mathscr {I}}}}}\le 1. \end{aligned}$$

Now, given that

$$\begin{aligned} 0\le \mathscr {B}_{\mathscr {S}_1}^{m}\le 1- \mathscr {A}_{\mathscr {S}_1}^{n} and 0\le \mathscr {B}_{\mathscr {S}_2}^{m}\le 1- \mathscr {A}_{\mathscr {S}_2}^{n}, \end{aligned}$$

afterward, we might acquire

$$\begin{aligned} (\root n \of {1-\textsf{e}^{-\left( \left( -ln \left( 1-\mathscr {A}_{\mathscr {S}_1}^{n}\right) \right) ^\mathscr {I}+\left( -ln \left( 1-\mathscr {A}_{\mathscr {S}_2}^{n}\right) \right) ^\mathscr {I}\right) ^{\frac{1}{\mathscr {I}}}}})^{n} + (\root m \of {\textsf{e}^{-\left( \left( -ln \mathscr {B}_{\mathscr {S}_1}^{m}\right) ^\mathscr {I}+\left( -ln \mathscr {B}_{\mathscr {S}_2}^{m}\right) ^\mathscr {I}\right) ^{\frac{1}{\mathscr {I}}}}})^{m} \end{aligned}$$

$$\begin{aligned} =1-\textsf{e}^{-\left( \left( -ln \left( 1-\mathscr {A}_{\mathscr {S}_1}^{n}\right) \right) ^\mathscr {I}+\left( -ln \left( 1-\mathscr {A}_{\mathscr {S}_2}^{n}\right) \right) ^\mathscr {I}\right) ^{\frac{1}{\mathscr {I}}}} + \textsf{e}^{-\left( \left( -ln \mathscr {B}_{\mathscr {S}_1}^{m}\right) ^\mathscr {I}+\left( -ln \mathscr {B}_{\mathscr {S}_2}^{m}\right) ^\mathscr {I}\right) ^{\frac{1}{\mathscr {I}}}} \end{aligned}$$

$$\begin{aligned} =1-\frac{1}{\textsf{e}^{\left( \left( -ln \left( 1-\mathscr {A}_{\mathscr {S}_1}^{n}\right) \right) ^\mathscr {I}+\left( -ln \left( 1-\mathscr {A}_{\mathscr {S}_2}^{n}\right) \right) ^\mathscr {I}\right) ^{\frac{1}{\mathscr {I}}}}} + \frac{1}{\textsf{e}^{\left( \left( -ln \mathscr {B}_{\mathscr {S}_1}^{m}\right) ^\mathscr {I}+\left( -ln \mathscr {B}_{\mathscr {S}_2}^{m}\right) ^\mathscr {I}\right) ^{\frac{1}{\mathscr {I}}}}} \end{aligned}$$

$$\begin{aligned} =1-(\frac{1}{\textsf{e}^{\left( -ln \left( 1-\mathscr {A}_{\mathscr {S}_1}^{n}\right) \right) ^\mathscr {I}} \cdot \textsf{e}^{\left( -ln \left( 1-\mathscr {A}_{\mathscr {S}_2}^{n}\right) \right) ^\mathscr {I}}})^{\frac{1}{\mathscr {I}}} + (\frac{1}{\textsf{e}^{\left( -ln \mathscr {B}_{\mathscr {S}_1}^{m}\right) ^{\mathscr {I}}} \cdot \textsf{e}^{\left( -ln \mathscr {B}_{\mathscr {S}_2}^{m}\right) ^\mathscr {I}}})^{\frac{1}{\mathscr {I}}} \end{aligned}$$

$$\begin{aligned} =1-\textsf{e}^{ln \left( 1-\mathscr {A}_{\mathscr {S}_1}^{n}\right) + ln \left( 1-\mathscr {A}_{\mathscr {S}_2}^{n}\right) } + \textsf{e}^{ln \mathscr {B}_{\mathscr {S}_1}^{m} + ln \mathscr {B}_{\mathscr {S}_2}^{m}} \end{aligned}$$

$$\begin{aligned} =1-\textsf{e}^{ln \left( \left( 1-\mathscr {A}_{\mathscr {S}_1}^{n}\right) \left( 1-\mathscr {A}_{\mathscr {S}_2}^{n}\right) \right) } + \textsf{e}^{ln \left( (\mathscr {B}_{\mathscr {S}_1}^{m})(\mathscr {B}_{\mathscr {S}_2}^{m})\right) } \end{aligned}$$

$$\begin{aligned} =1-\left( 1-\mathscr {A}_{\mathscr {S}_1}^{n}\right) \left( 1-\mathscr {A}_{\mathscr {S}_2}^{n}\right) + (\mathscr {B}_{\mathscr {S}_1}^{m})(\mathscr {B}_{\mathscr {S}_2}^{m}) \end{aligned}$$

$$\begin{aligned} \le 1-\mathscr {B}_{\mathscr {S}_1}^{m}\left( 1-\mathscr {A}_{\mathscr {S}_2}^{n}\right) + (\mathscr {B}_{\mathscr {S}_1}^{m})(\mathscr {B}_{\mathscr {S}_2}^{m}) \end{aligned}$$

$$\begin{aligned} \le 1-(\mathscr {B}_{\mathscr {S}_1}^{m})(\mathscr {B}_{\mathscr {S}_2}^{m}) + (\mathscr {B}_{\mathscr {S}_1}^{m})(\mathscr {B}_{\mathscr {S}_2}^{m}) = 1. \end{aligned}$$

Thus,

$$\begin{aligned} 0\le \root n \of {1-\textsf{e}^{-\left( \left( -ln \left( 1-\mathscr {A}_{\mathscr {S}_1}^{n}\right) \right) ^\mathscr {I}+\left( -ln \left( 1-\mathscr {A}_{\mathscr {S}_2}^{n}\right) \right) ^\mathscr {I}\right) ^{\frac{1}{\mathscr {I}}}}}\le 1, \end{aligned}$$

$$\begin{aligned} 0\le \root m \of {\textsf{e}^{-\left( \left( -ln \mathscr {B}_{\mathscr {S}_1}^{m}\right) ^\mathscr {I}+\left( -ln \mathscr {B}_{\mathscr {S}_2}^{m}\right) ^\mathscr {I}\right) ^{\frac{1}{\mathscr {I}}}}}\le 1 \end{aligned}$$

and

$$\begin{aligned} 0\le (\root n \of {1-\textsf{e}^{-\left( \left( -ln \left( 1-\mathscr {A}_{\mathscr {S}_1}^{n}\right) \right) ^\mathscr {I}+\left( -ln \left( 1-\mathscr {A}_{\mathscr {S}_2}^{n}\right) \right) ^\mathscr {I}\right) ^{\frac{1}{\mathscr {I}}}}})^{n} + (\root m \of {\textsf{e}^{-\left( \left( -ln \mathscr {B}_{\mathscr {S}_1}^{m}\right) ^\mathscr {I}+\left( -ln \mathscr {B}_{\mathscr {S}_2}^{m}\right) ^\mathscr {I}\right) ^{\frac{1}{\mathscr {I}}}}})^{m}\le 1. \end{aligned}$$

Similarly, we obtain

1. $$0\le \root n \of {1-\textsf{e}^{-\left( \left( -ln \left( 1-\mathscr {D}_{\mathscr {A}_{\mathscr {S}_1}}^{n}\right) \right) ^\mathscr {I}+\left( -ln \left( 1-\mathscr {D}_{\mathscr {A}_{\mathscr {S}_2}}^{n}\right) \right) ^\mathscr {I}\right) ^{\frac{1}{\mathscr {I}}}}}\le 1$$, $$0\le \root m \of {\textsf{e}^{-\left( \left( -ln \mathscr {D}_{\mathscr {B}_{\mathscr {S}_1}}^{m}\right) ^\mathscr {I}+\left( -ln \mathscr {D}_{\mathscr {B}_{\mathscr {S}_2}}^{m}\right) ^\mathscr {I}\right) ^{\frac{1}{\mathscr {I}}}}}\le 1$$ and
  $$\begin{aligned} 0\le (\root n \of {1-\textsf{e}^{-\left( \left( -ln \left( 1-\mathscr {D}_{\mathscr {A}_{\mathscr {S}_1}}^{n}\right) \right) ^\mathscr {I}+\left( -ln \left( 1-\mathscr {D}_{\mathscr {A}_{\mathscr {S}_2}}^{n}\right) \right) ^\mathscr {I}\right) ^{\frac{1}{\mathscr {I}}}}})^{n} + (\root m \of {\textsf{e}^{-\left( \left( -ln \mathscr {D}_{\mathscr {B}_{\mathscr {S}_1}}^{m}\right) ^\mathscr {I}+\left( -ln \mathscr {D}_{\mathscr {B}_{\mathscr {S}_2}}^{m}\right) ^\mathscr {I}\right) ^{\frac{1}{\mathscr {I}}}}})^{m}\le 1. \end{aligned}$$
2. $$0\le \root n \of {\textsf{e}^{-\left( \left( -ln \mathscr {A}_{\mathscr {S}_1}^{n}\right) ^\mathscr {I}+\left( -ln \mathscr {A}_{\mathscr {S}_2}^{n}\right) ^\mathscr {I}\right) ^{\frac{1}{\mathscr {I}}}}}\le 1$$, $$0\le \root m \of {1-\textsf{e}^{-\left( \left( -ln \left( 1-\mathscr {B}_{\mathscr {S}_1}^{m}\right) \right) ^\mathscr {I}+\left( -ln \left( 1-\mathscr {B}_{\mathscr {S}_2}^{m}\right) \right) ^\mathscr {I}\right) ^{\frac{1}{\mathscr {I}}}}}\le 1$$ and
  $$\begin{aligned} 0\le (\root n \of {\textsf{e}^{-\left( \left( -ln \mathscr {A}_{\mathscr {S}_1}^{n}\right) ^\mathscr {I}+\left( -ln \mathscr {A}_{\mathscr {S}_2}^{n}\right) ^\mathscr {I}\right) ^{\frac{1}{\mathscr {I}}}}})^{n} + (\root m \of {1-\textsf{e}^{-\left( \left( -ln \left( 1-\mathscr {B}_{\mathscr {S}_1}^{m}\right) \right) ^\mathscr {I}+\left( -ln \left( 1-\mathscr {B}_{\mathscr {S}_2}^{m}\right) \right) ^\mathscr {I}\right) ^{\frac{1}{\mathscr {I}}}}})^{m}\le 1. \end{aligned}$$
3. $$0\le \root n \of {\textsf{e}^{-\left( \left( -ln \mathscr {D}_{\mathscr {A}_{\mathscr {S}_1}}^{n}\right) ^\mathscr {I}+\left( -ln \mathscr {D}_{\mathscr {A}_{\mathscr {S}_2}}^{n}\right) ^\mathscr {I}\right) ^{\frac{1}{\mathscr {I}}}}}\le 1$$, $$0\le \root m \of {1-\textsf{e}^{-\left( \left( -ln \left( 1-\mathscr {D}_{\mathscr {B}_{\mathscr {S}_1}}^{m}\right) \right) ^\mathscr {I}+\left( -ln \left( 1-\mathscr {D}_{\mathscr {B}_{\mathscr {S}_2}}^{m}\right) \right) ^\mathscr {I}\right) ^{\frac{1}{\mathscr {I}}}}}\le 1$$ and
  $$\begin{aligned} 0\le (\root n \of {\textsf{e}^{-\left( \left( -ln \mathscr {D}_{\mathscr {A}_{\mathscr {S}_1}}^{n}\right) ^\mathscr {I}+\left( -ln \mathscr {D}_{\mathscr {A}_{\mathscr {S}_2}}^{n}\right) ^\mathscr {I}\right) ^{\frac{1}{\mathscr {I}}}}})^{n} + (\root m \of {1-\textsf{e}^{-\left( \left( -ln \left( 1-\mathscr {D}_{\mathscr {B}_{\mathscr {S}_1}}^{m}\right) \right) ^\mathscr {I}+\left( -ln \left( 1-\mathscr {D}_{\mathscr {B}_{\mathscr {S}_2}}^{m}\right) \right) ^\mathscr {I}\right) ^{\frac{1}{\mathscr {I}}}}})^{m}\le 1. \end{aligned}$$

This shows that both $\mathscr {S}_1 \boxplus \mathscr {S}_2$ and $\mathscr {S}_1 \boxtimes \mathscr {S}_2$ are Cn,m-ROFNs. $\square$

### Theorem 3.4

Let $\mathscr {S} =(\mathscr {A}_{\mathscr {S}} \cdot e^{i.2 \pi \mathscr {D}_{\mathscr {A}_{\mathscr {S}}}}, \mathscr {B}_{\mathscr {S}} \cdot e^{i \cdot 2 \pi \mathscr {D}_{\mathscr {B}_{\mathscr {S}}}})$ be a Cn,m-ROFN, and let $\mathscr {L} > 0$. Then, both $\mathscr {L} \mathscr {S}$ and $\mathscr {S}^{\mathscr {L}}$ are also Cn,m-ROFNs.

### Proof

The inequalities below are valid:

$$\begin{aligned} 0 \le \mathscr {A}_{\mathscr {S}}^n+\mathscr {B}_{\mathscr {S}}^m \le 1 \text {and} 0 \le \mathscr {D}_{\mathscr {A}_{\mathscr {S}}}^n+\mathscr {D}_{\mathscr {B}_{\mathscr {S}}}^m \le 1 , \end{aligned}$$

from which we can derive:

$$\begin{aligned} 0\le 1-\mathscr {A}_{\mathscr {S}}^{n} \text {and} 0\le \mathscr {B}_{\mathscr {S}}^{m} \end{aligned}$$

this implies that:

$$\begin{aligned} 0\le -ln(1-\mathscr {A}_{\mathscr {S}}^{n}) \text {and} 0\le -ln(\mathscr {B}_{\mathscr {S}}^{m}) . \end{aligned}$$

Hence, we have:

$$\begin{aligned} \mathscr {L}\left( -ln \left( 1-\mathscr {A}_{\mathscr {S}}^{n}\right) \right) ^\mathscr {I}\ge 0 \end{aligned}$$

and

$$\begin{aligned} \mathscr {L}\left( -ln \left( \mathscr {B}_{\mathscr {S}}^{m}\right) \right) ^\mathscr {I}\ge 0, \end{aligned}$$

therefore

$$\begin{aligned} -\left( \mathscr {L}\left( -ln \left( 1-\mathscr {A}_{\mathscr {S}}^{n}\right) \right) ^\mathscr {I}\right) ^{\frac{1}{\mathscr {I}}}\le 0 \end{aligned}$$

and

$$\begin{aligned} -\left( \mathscr {L}\left( -ln \left( \mathscr {B}_{\mathscr {S}}^{m}\right) \right) ^\mathscr {I}\right) ^{\frac{1}{\mathscr {I}}}\le 0 \end{aligned}$$

this demonstrates that:

$$\begin{aligned} 1-\textsf{e}^{-\left( \mathscr {L}\left( -ln \left( 1-\mathscr {A}_{\mathscr {S}}^{n}\right) \right) ^\mathscr {I}\right) ^{\frac{1}{\mathscr {I}}}}\ge 0 \end{aligned}$$

implies

$$\begin{aligned} \root n \of {1-\textsf{e}^{-\left( \mathscr {L}\left( -ln \left( 1-\mathscr {A}_{\mathscr {S}}^{n}\right) \right) ^\mathscr {I}\right) ^{\frac{1}{\mathscr {I}}}}}\ge 0 . \end{aligned}$$

Similarly, we also have:

$$\begin{aligned} \textsf{e}^{-\left( \mathscr {L}\left( -ln \left( \mathscr {B}_{\mathscr {S}}^{m}\right) \right) ^\mathscr {I}\right) ^{\frac{1}{\mathscr {I}}}}\ge 0 \end{aligned}$$

implies

$$\begin{aligned} \root m \of {\textsf{e}^{-\left( \mathscr {L}\left( -ln \left( \mathscr {B}_{\mathscr {S}}^{m}\right) \right) ^\mathscr {I}\right) ^{\frac{1}{\mathscr {I}}}}}\ge 0 . \end{aligned}$$

It is clear that

$$\begin{aligned} \root n \of {1-\textsf{e}^{-\left( \mathscr {L}\left( -ln \left( 1-\mathscr {A}_{\mathscr {S}}^{n}\right) \right) ^\mathscr {I}\right) ^{\frac{1}{\mathscr {I}}}}} \le 1 \end{aligned}$$

and

$$\begin{aligned} \root m \of {\textsf{e}^{-\left( \mathscr {L}\left( -ln \left( \mathscr {B}_{\mathscr {S}}^{m}\right) \right) ^\mathscr {I}\right) ^{\frac{1}{\mathscr {I}}}}}\le 1 . \end{aligned}$$

Next, we know that:

$$\begin{aligned} 0\le \mathscr {B}_{\mathscr {S}}^{m}\le 1- \mathscr {A}_{\mathscr {S}}^{n} \end{aligned}$$

which implies:

$$\begin{aligned} (\mathscr {B}_{\mathscr {S}}^{m})^{\mathscr {L}^{\frac{1}{\mathscr {I}}}}\le (1- \mathscr {A}_{\mathscr {S}}^{n})^{\mathscr {L}^{\frac{1}{\mathscr {I}}}}. \end{aligned}$$

Thus, we can obtain:

$$\begin{aligned} (\root n \of {1-\textsf{e}^{-\left( \mathscr {L}\left( -ln \left( 1-\mathscr {A}_{\mathscr {S}}^{n}\right) \right) ^\mathscr {I}\right) ^{\frac{1}{\mathscr {I}}}}})^{n} + (\root m \of {\textsf{e}^{-\left( \mathscr {L}\left( -ln \left( \mathscr {B}_{\mathscr {S}}^{m}\right) \right) ^\mathscr {I}\right) ^{\frac{1}{\mathscr {I}}}}})^{m} \end{aligned}$$

$$\begin{aligned} =1-\textsf{e}^{-\left( \mathscr {L}\left( -ln \left( 1-\mathscr {A}_{\mathscr {S}}^{n}\right) \right) ^\mathscr {I}\right) ^{\frac{1}{\mathscr {I}}}} + \textsf{e}^{-\left( \mathscr {L}\left( -ln \left( \mathscr {B}_{\mathscr {S}}^{m}\right) \right) ^\mathscr {I}\right) ^{\frac{1}{\mathscr {I}}}} \end{aligned}$$

$$\begin{aligned} =1-\frac{1}{\textsf{e}^{\left( \mathscr {L}\left( -ln \left( 1-\mathscr {A}_{\mathscr {S}}^{n}\right) \right) ^\mathscr {I}\right) ^{\frac{1}{\mathscr {I}}}}} + \frac{1}{\textsf{e}^{\left( \mathscr {L}\left( -ln \left( \mathscr {B}_{\mathscr {S}}^{m}\right) \right) ^\mathscr {I}\right) ^{\frac{1}{\mathscr {I}}}}} \end{aligned}$$

$$\begin{aligned} =1-\frac{1}{\textsf{e}^{\mathscr {L}^{\frac{1}{\mathscr {I}}}\left( -ln \left( 1-\mathscr {A}_{\mathscr {S}}^{n}\right) \right) }} + \frac{1}{\textsf{e}^{\mathscr {L}^{\frac{1}{\mathscr {I}}}\left( -ln \left( \mathscr {B}_{\mathscr {S}}^{m}\right) \right) }} \end{aligned}$$

$$\begin{aligned} =1-\textsf{e}^{ln \left( 1-\mathscr {A}_{\mathscr {S}}^{n}\right) ^{\mathscr {L}^{\frac{1}{\mathscr {I}}}}} + \textsf{e}^{ln \left( \mathscr {B}_{\mathscr {S}}^{m}\right) ^{\mathscr {L}^{\frac{1}{\mathscr {I}}}}} \end{aligned}$$

$$\begin{aligned} =1-\left( 1-\mathscr {A}_{\mathscr {S}}^{n}\right) ^{\mathscr {L}^{\frac{1}{\mathscr {I}}}} + \left( \mathscr {B}_{\mathscr {S}}^{m}\right) ^{\mathscr {L}^{\frac{1}{\mathscr {I}}}} \end{aligned}$$

$$\begin{aligned} \le 1-\left( \mathscr {B}_{\mathscr {S}}^{m}\right) ^{\mathscr {L}^{\frac{1}{\mathscr {I}}}} + \left( \mathscr {B}_{\mathscr {S}}^{m}\right) ^{\mathscr {L}^{\frac{1}{\mathscr {I}}}}= 1. \end{aligned}$$

Hence, we conclude

$$\begin{aligned} 0\le \root n \of {1-\textsf{e}^{-\left( \mathscr {L}\left( -ln \left( 1-\mathscr {A}_{\mathscr {S}}^{n}\right) \right) ^\mathscr {I}\right) ^{\frac{1}{\mathscr {I}}}}}\le 1, \end{aligned}$$

$$\begin{aligned} 0\le \root m \of {\textsf{e}^{-\left( \mathscr {L}\left( -ln \left( \mathscr {B}_{\mathscr {S}}^{m}\right) \right) ^\mathscr {I}\right) ^{\frac{1}{\mathscr {I}}}}}\le 1 \end{aligned}$$

and

$$\begin{aligned} 0\le (\root n \of {1-\textsf{e}^{-\left( \mathscr {L}\left( -ln \left( 1-\mathscr {A}_{\mathscr {S}}^{n}\right) \right) ^\mathscr {I}\right) ^{\frac{1}{\mathscr {I}}}}})^{n} + (\root m \of {\textsf{e}^{-\left( \mathscr {L}\left( -ln \left( \mathscr {B}_{\mathscr {S}}^{m}\right) \right) ^\mathscr {I}\right) ^{\frac{1}{\mathscr {I}}}}})^{m}\le 1. \end{aligned}$$

Similarly, we can also derive the following results:

1. $$0\le \root n \of {1-\textsf{e}^{-\left( \mathscr {L}\left( -ln \left( 1-\mathscr {D}_{\mathscr {A}_{\mathscr {S}}}^{n}\right) \right) ^\mathscr {I}\right) ^{\frac{1}{\mathscr {I}}}}}\le 1$$, $$0\le \root m \of {\textsf{e}^{-\left( \mathscr {L}\left( -ln \mathscr {D}_{\mathscr {B}_{\mathscr {S}}}^{m}\right) ^\mathscr {I}\right) ^{\frac{1}{\mathscr {I}}}}}\le 1$$ and
  $$\begin{aligned} 0\le (\root n \of {1-\textsf{e}^{-\left( \mathscr {L}\left( -ln \left( 1-\mathscr {D}_{\mathscr {A}_{\mathscr {S}}}^{n}\right) \right) ^\mathscr {I}\right) ^{\frac{1}{\mathscr {I}}}}})^{n} + (\root m \of {\textsf{e}^{-\left( \mathscr {L}\left( -ln \mathscr {D}_{\mathscr {B}_{\mathscr {S}}}^{m}\right) ^\mathscr {I}\right) ^{\frac{1}{\mathscr {I}}}}})^{m}\le 1 . \end{aligned}$$
2. $$0\le \root n \of {\textsf{e}^{-\left( \mathscr {L}\left( -ln \mathscr {A}_{\mathscr {S}}^{n}\right) ^\mathscr {I}\right) ^{\frac{1}{\mathscr {I}}}}}\le 1$$, $$0\le \root m \of {1-\textsf{e}^{-\left( \mathscr {L}\left( -ln \left( 1-\mathscr {B}_{\mathscr {S}}^{m}\right) \right) ^\mathscr {I}\right) ^{\frac{1}{\mathscr {I}}}}}\le 1$$ and
  $$\begin{aligned} 0\le (\root n \of {\textsf{e}^{-\left( \mathscr {L}\left( -ln \mathscr {A}_{\mathscr {S}}^{n}\right) ^\mathscr {I}\right) ^{\frac{1}{\mathscr {I}}}}})^{n} + (\root m \of {1-\textsf{e}^{-\left( \mathscr {L}\left( -ln \left( 1-\mathscr {B}_{\mathscr {S}}^{m}\right) \right) ^\mathscr {I}\right) ^{\frac{1}{\mathscr {I}}}}})^{m}\le 1. \end{aligned}$$
3. $$0\le \root n \of {\textsf{e}^{-\left( \mathscr {L}\left( -ln \mathscr {D}_{\mathscr {A}_{\mathscr {S}}}^{n}\right) ^\mathscr {I}\right) ^{\frac{1}{\mathscr {I}}}}}\le 1$$, $$0\le \root m \of {1-\textsf{e}^{-\left( \mathscr {L}\left( -ln \left( 1-\mathscr {D}_{\mathscr {B}_{\mathscr {S}}}^{m}\right) \right) ^\mathscr {I}\right) ^{\frac{1}{\mathscr {I}}}}}\le 1$$ and
  $$\begin{aligned} 0\le (\root n \of {\textsf{e}^{-\left( \mathscr {L}\left( -ln \mathscr {D}_{\mathscr {A}_{\mathscr {S}}}^{n}\right) ^\mathscr {I}\right) ^{\frac{1}{\mathscr {I}}}}})^{n} + (\root m \of {1-\textsf{e}^{-\left( \mathscr {L}\left( -ln \left( 1-\mathscr {D}_{\mathscr {B}_{\mathscr {S}}}^{m}\right) \right) ^\mathscr {I}\right) ^{\frac{1}{\mathscr {I}}}}})^{m}\le 1. \end{aligned}$$

Thus, $\mathscr {L} \mathscr {S}$ and $\mathscr {S}^{\mathscr {L}}$ are Cn,m-ROFNs. $\square$

### Theorem 3.5

Let $$\mathscr {S}_1=(\mathscr {A}_{\mathscr {S}_1} \cdot e^{i.2 \pi \mathscr {D}_{\mathscr {A}_{\mathscr {S}_1}}}, \mathscr {B}_{\mathscr {S}_1} \cdot e^{i \cdot 2 \pi \mathscr {D}_{\mathscr {B}_{\mathscr {S}_1}}})$$ and

$\mathscr {S}_2=(\mathscr {A}_{\mathscr {S}_2} \cdot e^{i.2 \pi \mathscr {D}_{\mathscr {A}_{\mathscr {S}_2}}}, \mathscr {B}_{\mathscr {S}_2} \cdot e^{i \cdot 2 \pi \mathscr {D}_{\mathscr {B}_{\mathscr {S}_2}}})$ be two Cn,m-ROFNs. Then,

1. $$\mathscr {S}_{1} \boxplus \mathscr {S}_{2}=\mathscr {S}_{2} \boxplus \mathscr {S}_{1}$$.
2. $$\mathscr {S}_{1} \boxtimes \mathscr {S}_{2}=\mathscr {S}_{2} \boxtimes \mathscr {S}_{1}$$.

### Proof

1. $$\mathscr {S}_1 \boxplus \mathscr {S}_2 =\left( \begin{array}{c} \root n \of {1-\textsf{e}^{-\left( \left( -ln \left( 1-\mathscr {A}_{\mathscr {S}_1}^{n}\right) \right) ^\mathscr {I}+\left( -ln \left( 1-\mathscr {A}_{\mathscr {S}_2}^{n}\right) \right) ^\mathscr {I}\right) ^{\frac{1}{\mathscr {I}}}}}\\ \textsf{e}^{2 i \pi \root n \of {1-\textsf{e}^{-\left( \left( -ln \left( 1-\mathscr {D}_{\mathscr {A}_{\mathscr {S}_1}}^{n}\right) \right) ^\mathscr {I}+\left( -ln \left( 1-\mathscr {D}_{\mathscr {A}_{\mathscr {S}_2}}^{n}\right) \right) ^\mathscr {I}\right) ^{\frac{1}{\mathscr {I}}}}}},\\ \root m \of {\textsf{e}^{-\left( \left( -ln \mathscr {B}_{\mathscr {S}_1}^{m}\right) ^\mathscr {I}+\left( -ln \mathscr {B}_{\mathscr {S}_2}^{m}\right) ^\mathscr {I}\right) ^{\frac{1}{\mathscr {I}}}}}\\ \textsf{e}^{2 i \pi \root m \of {\textsf{e}^{-\left( \left( -ln \mathscr {D}_{\mathscr {B}_{\mathscr {S}_1}}^{m}\right) ^\mathscr {I}+\left( -ln \mathscr {D}_{\mathscr {B}_{\mathscr {S}_2}}^{m}\right) ^\mathscr {I}\right) ^{\frac{1}{\mathscr {I}}}}}} \end{array}\right)$$
  $$=\left( \begin{array}{c} \root n \of {1-\textsf{e}^{-\left( \left( -ln \left( 1-\mathscr {A}_{\mathscr {S}_2}^{n}\right) \right) ^\mathscr {I}+\left( -ln \left( 1-\mathscr {A}_{\mathscr {S}_1}^{n}\right) \right) ^\mathscr {I}\right) ^{\frac{1}{\mathscr {I}}}}}\\ \textsf{e}^{2 i \pi \root n \of {1-\textsf{e}^{-\left( \left( -ln \left( 1-\mathscr {D}_{\mathscr {A}_{\mathscr {S}_2}}^{n}\right) \right) ^\mathscr {I}+\left( -ln \left( 1-\mathscr {D}_{\mathscr {A}_{\mathscr {S}_1}}^{n}\right) \right) ^\mathscr {I}\right) ^{\frac{1}{\mathscr {I}}}}}},\\ \root m \of {\textsf{e}^{-\left( \left( -ln \mathscr {B}_{\mathscr {S}_2}^{m}\right) ^\mathscr {I}+\left( -ln \mathscr {B}_{\mathscr {S}_1}^{m}\right) ^\mathscr {I}\right) ^{\frac{1}{\mathscr {I}}}}}\\ \textsf{e}^{2 i \pi \root m \of {\textsf{e}^{-\left( \left( -ln \mathscr {D}_{\mathscr {B}_{\mathscr {S}_2}}^{m}\right) ^\mathscr {I}+\left( -ln \mathscr {D}_{\mathscr {B}_{\mathscr {S}_1}}^{m}\right) ^\mathscr {I}\right) ^{\frac{1}{\mathscr {I}}}}}} \end{array}\right) = \mathscr {S}_2 \boxplus \mathscr {S}_1$$
2. $$\mathscr {S}_1\boxtimes \mathscr {S}_2=\left( \begin{array}{c} \root n \of {\textsf{e}^{-\left( \left( -ln \mathscr {A}_{\mathscr {S}_1}^{n}\right) ^\mathscr {I}+\left( -ln \mathscr {A}_{\mathscr {S}_2}^{n}\right) ^\mathscr {I}\right) ^{\frac{1}{\mathscr {I}}}}}\\ \textsf{e}^{2 i \pi \root n \of {\textsf{e}^{-\left( \left( -ln \mathscr {D}_{\mathscr {A}_{\mathscr {S}_1}}^{n}\right) ^\mathscr {I}+\left( -ln \mathscr {D}_{\mathscr {A}_{\mathscr {S}_2}}^{n}\right) ^\mathscr {I}\right) ^{\frac{1}{\mathscr {I}}}}}},\\ \root m \of {1-\textsf{e}^{-\left( \left( -ln \left( 1-\mathscr {B}_{\mathscr {S}_1}^{m}\right) \right) ^\mathscr {I}+\left( -ln \left( 1-\mathscr {B}_{\mathscr {S}_2}^{m}\right) \right) ^\mathscr {I}\right) ^{\frac{1}{\mathscr {I}}}}}\\ \textsf{e}^{2 i \pi \root m \of {1-\textsf{e}^{-\left( \left( -ln \left( 1-\mathscr {D}_{\mathscr {B}_{\mathscr {S}_1}}^{m}\right) \right) ^\mathscr {I}+\left( -ln \left( 1-\mathscr {D}_{\mathscr {B}_{\mathscr {S}_2}}^{m}\right) \right) ^\mathscr {I}\right) ^{\frac{1}{\mathscr {I}}}}}} \end{array}\right)$$
  $$=\left( \begin{array}{c} \root n \of {\textsf{e}^{-\left( \left( -ln \mathscr {A}_{\mathscr {S}_2}^{n}\right) ^\mathscr {I}+\left( -ln \mathscr {A}_{\mathscr {S}_1}^{n}\right) ^\mathscr {I}\right) ^{\frac{1}{\mathscr {I}}}}}\\ \textsf{e}^{2 i \pi \root n \of {\textsf{e}^{-\left( \left( -ln \mathscr {D}_{\mathscr {A}_{\mathscr {S}_2}}^{n}\right) ^\mathscr {I}+\left( -ln \mathscr {D}_{\mathscr {A}_{\mathscr {S}_1}}^{n}\right) ^\mathscr {I}\right) ^{\frac{1}{\mathscr {I}}}}}},\\ \root m \of {1-\textsf{e}^{-\left( \left( -ln \left( 1-\mathscr {B}_{\mathscr {S}_2}^{m}\right) \right) ^\mathscr {I}+\left( -ln \left( 1-\mathscr {B}_{\mathscr {S}_1}^{m}\right) \right) ^\mathscr {I}\right) ^{\frac{1}{\mathscr {I}}}}}\\ \textsf{e}^{2 i \pi \root m \of {1-\textsf{e}^{-\left( \left( -ln \left( 1-\mathscr {D}_{\mathscr {B}_{\mathscr {S}_2}}^{m}\right) \right) ^\mathscr {I}+\left( -ln \left( 1-\mathscr {D}_{\mathscr {B}_{\mathscr {S}_1}}^{m}\right) \right) ^\mathscr {I}\right) ^{\frac{1}{\mathscr {I}}}}}} \end{array}\right) =\mathscr {S}_2\boxtimes \mathscr {S}_1$$

$$\square$$

### Theorem 3.6

Let $\mathscr {S}_1\!=\!(\mathscr {A}_{\mathscr {S}_1}\! \cdot\! e^{i.2 \pi \mathscr {D}_{\mathscr {A}_{\mathscr {S}_1}}}, \mathscr {B}_{\mathscr {S}_1}\!\cdot \!e^{i \cdot 2 \pi \mathscr {D}_{\mathscr {B}_{\mathscr {S}_1}}})$ and $\mathscr {S}_2\!=\!(\mathscr {A}_{\mathscr {S}_2}\! \cdot\! e^{i.2 \pi \mathscr {D}_{\mathscr {A}_{\mathscr {S}_2}}}, \mathscr {B}_{\mathscr {S}_2} \!\cdot \!e^{i \cdot 2 \pi \mathscr {D}_{\mathscr {B}_{\mathscr {S}_2}}})$ be two Cn,m-ROFNs, and $\mathscr {L}>0$. Then,

1. $$\mathscr {L}(\mathscr {S}_{1} \boxplus \mathscr {S}_{2})=\mathscr {L} \mathscr {S}_{1} \boxplus \mathscr {L} \mathscr {S}_{2}$$
2. $$(\mathscr {S}_{1} \boxtimes \mathscr {S}_{2})^{\mathscr {L}}=\mathscr {S}_{1}^{\mathscr {L}} \boxtimes \mathscr {S}_{2}^{\mathscr {L}}$$

### Proof

1. $$\mathscr {L}(\mathscr {S}_1 \boxplus \mathscr {S}_2) = \mathscr {L}\left( \begin{array}{c} \root n \of {1-\textsf{e}^{-\left( \left( -ln \left( 1-\mathscr {A}_{\mathscr {S}_1}^{n}\right) \right) ^\mathscr {I}+\left( -ln \left( 1-\mathscr {A}_{\mathscr {S}_2}^{n}\right) \right) ^\mathscr {I}\right) ^{\frac{1}{\mathscr {I}}}}}\\ \textsf{e}^{2 i \pi \root n \of {1-\textsf{e}^{-\left( \left( -ln \left( 1-\mathscr {D}_{\mathscr {A}_{\mathscr {S}_1}}^{n}\right) \right) ^\mathscr {I}+\left( -ln \left( 1-\mathscr {D}_{\mathscr {A}_{\mathscr {S}_2}}^{n}\right) \right) ^\mathscr {I}\right) ^{\frac{1}{\mathscr {I}}}}}},\\ \root m \of {\textsf{e}^{-\left( \left( -ln \mathscr {B}_{\mathscr {S}_1}^{m}\right) ^\mathscr {I}+\left( -ln \mathscr {B}_{\mathscr {S}_2}^{m}\right) ^\mathscr {I}\right) ^{\frac{1}{\mathscr {I}}}}}\\ \textsf{e}^{2 i \pi \root m \of {\textsf{e}^{-\left( \left( -ln \mathscr {D}_{\mathscr {B}_{\mathscr {S}_1}}^{m}\right) ^\mathscr {I}+\left( -ln \mathscr {D}_{\mathscr {B}_{\mathscr {S}_2}}^{m}\right) ^\mathscr {I}\right) ^{\frac{1}{\mathscr {I}}}}}} \end{array}\right)$$ $$= \left( \begin{array}{c} \root n \of {1-\textsf{e}^{-\left( \mathscr {L}\left( -ln \left( 1-\mathscr {A}_{\mathscr {S}_1}^{n}\right) \right) ^\mathscr {I} + \mathscr {L}\left( -ln \left( 1-\mathscr {A}_{\mathscr {S}_2}^{n}\right) \right) ^\mathscr {I}\right) ^{\frac{1}{\mathscr {I}}}}}\\ \textsf{e}^{2 i \pi \root n \of {1-\textsf{e}^{-\left( \mathscr {L}\left( -ln \left( 1-\mathscr {D}_{\mathscr {A}_{\mathscr {S}_1}}^{n}\right) \right) ^\mathscr {I} + \mathscr {L}\left( -ln \left( 1-\mathscr {D}_{\mathscr {A}_{\mathscr {S}_2}}^{n}\right) \right) ^\mathscr {I}\right) ^{\frac{1}{\mathscr {I}}}}}},\\ \root m \of {\textsf{e}^{-\left( \mathscr {L}\left( -ln \mathscr {B}_{\mathscr {S}_1}^{m}\right) ^\mathscr {I}+\mathscr {L}\left( -ln \mathscr {B}_{\mathscr {S}_2}^{m}\right) ^\mathscr {I}\right) ^{\frac{1}{\mathscr {I}}}}}\\ \textsf{e}^{2 i \pi \root m \of {\textsf{e}^{-\left( \mathscr {L}\left( -ln \mathscr {D}_{\mathscr {B}_{\mathscr {S}_1}}^{m}\right) ^\mathscr {I}+\mathscr {L}\left( -ln \mathscr {D}_{\mathscr {B}_{\mathscr {S}_2}}^{m}\right) ^\mathscr {I}\right) ^{\frac{1}{\mathscr {I}}}}}} \end{array}\right)$$ $$= \left( \begin{array}{c} \root n \of {1-\textsf{e}^{-\left( \mathscr {L}\left( -ln \left( 1-\mathscr {A}_{\mathscr {S}_1}^{n}\right) \right) ^\mathscr {I}\right) ^{\frac{1}{\mathscr {I}}}}}\\ \textsf{e}^{2 i \pi \root n \of {1-\textsf{e}^{-\left( \mathscr {L}\left( -ln \left( 1-\mathscr {D}_{\mathscr {A}_{\mathscr {S}_1}}^{n}\right) \right) ^\mathscr {I}\right) ^{\frac{1}{\mathscr {I}}}}}},\\ \root m \of {\textsf{e}^{-\left( \mathscr {L}\left( -ln \mathscr {B}_{\mathscr {S}_1}^{m}\right) ^\mathscr {I}\right) ^{\frac{1}{\mathscr {I}}}}}\\ \textsf{e}^{2 i \pi \root m \of {\textsf{e}^{-\left( \mathscr {L}\left( -ln \mathscr {D}_{\mathscr {B}_{\mathscr {S}_1}}^{m}\right) ^\mathscr {I}\right) ^{\frac{1}{\mathscr {I}}}}}} \end{array}\right) \boxplus \left( \begin{array}{c} \root n \of {1-\textsf{e}^{-\left( \mathscr {L}\left( -ln \left( 1-\mathscr {A}_{\mathscr {S}_2}^{n}\right) \right) ^\mathscr {I}\right) ^{\frac{1}{\mathscr {I}}}}}\\ \textsf{e}^{2 i \pi \root n \of {1-\textsf{e}^{-\left( \mathscr {L}\left( -ln \left( 1-\mathscr {D}_{\mathscr {A}_{\mathscr {S}_2}}^{n}\right) \right) ^\mathscr {I}\right) ^{\frac{1}{\mathscr {I}}}}}},\\ \root m \of {\textsf{e}^{-\left( \mathscr {L}\left( -ln \mathscr {B}_{\mathscr {S}_2}^{m}\right) ^\mathscr {I}\right) ^{\frac{1}{\mathscr {I}}}}}\\ \textsf{e}^{2 i \pi \root m \of {\textsf{e}^{-\left( \mathscr {L}\left( -ln \mathscr {D}_{\mathscr {B}_{\mathscr {S}_2}}^{m}\right) ^\mathscr {I}\right) ^{\frac{1}{\mathscr {I}}}}}} \end{array}\right) =\mathscr {L} \mathscr {S}_1 \boxplus \mathscr {L} \mathscr {S}_2.$$
2. $$(\mathscr {S}_1 \boxtimes \mathscr {S}_2)^{\mathscr {L}}= \left( \begin{array}{c} \root n \of {\textsf{e}^{-\left( \left( -ln \mathscr {A}_{\mathscr {S}_1}^{n}\right) ^\mathscr {I}+\left( -ln \mathscr {A}_{\mathscr {S}_2}^{n}\right) ^\mathscr {I}\right) ^{\frac{1}{\mathscr {I}}}}}\\ \textsf{e}^{2 i \pi \root n \of {\textsf{e}^{-\left( \left( -ln \mathscr {D}_{\mathscr {A}_{\mathscr {S}_1}}^{n}\right) ^\mathscr {I}+\left( -ln \mathscr {D}_{\mathscr {A}_{\mathscr {S}_2}}^{n}\right) ^\mathscr {I}\right) ^{\frac{1}{\mathscr {I}}}}}},\\ \root m \of {1-\textsf{e}^{-\left( \left( -ln \left( 1-\mathscr {B}_{\mathscr {S}_1}^{m}\right) \right) ^\mathscr {I}+\left( -ln \left( 1-\mathscr {B}_{\mathscr {S}_2}^{m}\right) \right) ^\mathscr {I}\right) ^{\frac{1}{\mathscr {I}}}}}\\ \textsf{e}^{2 i \pi \root m \of {1-\textsf{e}^{-\left( \left( -ln \left( 1-\mathscr {D}_{\mathscr {B}_{\mathscr {S}_1}}^{m}\right) \right) ^\mathscr {I}+\left( -ln \left( 1-\mathscr {D}_{\mathscr {B}_{\mathscr {S}_2}}^{m}\right) \right) ^\mathscr {I}\right) ^{\frac{1}{\mathscr {I}}}}}} \end{array}\right) ^{\mathscr {L}}=$$
$$\left( \begin{array}{c} \root n \of {\textsf{e}^{-\left( {\mathscr {L}}\left( -ln \mathscr {A}_{\mathscr {S}_1}^{n}\right) ^\mathscr {I}+{\mathscr {L}}\left( -ln \mathscr {A}_{\mathscr {S}_2}^{n}\right) ^\mathscr {I}\right) ^{\frac{1}{\mathscr {I}}}}}\\ \textsf{e}^{2 i \pi \root n \of {\textsf{e}^{-\left( {\mathscr {L}}\left( -ln \mathscr {D}_{\mathscr {A}_{\mathscr {S}_1}}^{n}\right) ^\mathscr {I}+{\mathscr {L}}\left( -ln \mathscr {D}_{\mathscr {A}_{\mathscr {S}_2}}^{n}\right) ^\mathscr {I}\right) ^{\frac{1}{\mathscr {I}}}}}},\\ \root m \of {1-\textsf{e}^{-\left( {\mathscr {L}}\left( -ln \left( 1-\mathscr {B}_{\mathscr {S}_1}^{m}\right) \right) ^\mathscr {I}+{\mathscr {L}}\left( -ln \left( 1-\mathscr {B}_{\mathscr {S}_2}^{m}\right) \right) ^\mathscr {I}\right) ^{\frac{1}{\mathscr {I}}}}}\\ \textsf{e}^{2 i \pi \root m \of {1-\textsf{e}^{-\left( {\mathscr {L}}\left( -ln \left( 1-\mathscr {D}_{\mathscr {B}_{\mathscr {S}_1}}^{m}\right) \right) ^\mathscr {I}+{\mathscr {L}}\left( -ln \left( 1-\mathscr {D}_{\mathscr {B}_{\mathscr {S}_2}}^{m}\right) \right) ^\mathscr {I}\right) ^{\frac{1}{\mathscr {I}}}}}} \end{array}\right) =$$
$$\left( \begin{array}{c} \root n \of {\textsf{e}^{-\left( \mathscr {L}\left( -ln \mathscr {A}_{\mathscr {S}_1}^{n}\right) ^\mathscr {I}\right) ^{\frac{1}{\mathscr {I}}}}}\\ \textsf{e}^{2 i \pi \root n \of {\textsf{e}^{-\left( \mathscr {L}\left( -ln \mathscr {D}_{\mathscr {A}_{\mathscr {S}_1}}^{n}\right) ^\mathscr {I}\right) ^{\frac{1}{\mathscr {I}}}}}},\\ \root m \of {1-\textsf{e}^{-\left( \mathscr {L}\left( -ln \left( 1-\mathscr {B}_{\mathscr {S}_1}^{m}\right) \right) ^\mathscr {I}\right) ^{\frac{1}{\mathscr {I}}}}}\\ \textsf{e}^{2 i \pi \root m \of {1-\textsf{e}^{-\left( \mathscr {L}\left( -ln \left( 1-\mathscr {D}_{\mathscr {B}_{\mathscr {S}_1}}^{m}\right) \right) ^\mathscr {I}\right) ^{\frac{1}{\mathscr {I}}}}}} \end{array}\right) \boxtimes \left( \begin{array}{c} \root n \of {\textsf{e}^{-\left( \mathscr {L}\left( -ln \mathscr {A}_{\mathscr {S}_2}^{n}\right) ^\mathscr {I}\right) ^{\frac{1}{\mathscr {I}}}}}\\ \textsf{e}^{2 i \pi \root n \of {\textsf{e}^{-\left( \mathscr {L}\left( -ln \mathscr {D}_{\mathscr {A}_{\mathscr {S}_2}}^{n}\right) ^\mathscr {I}\right) ^{\frac{1}{\mathscr {I}}}}}},\\ \root m \of {1-\textsf{e}^{-\left( \mathscr {L}\left( -ln \left( 1-\mathscr {B}_{\mathscr {S}_2}^{m}\right) \right) ^\mathscr {I}\right) ^{\frac{1}{\mathscr {I}}}}}\\ \textsf{e}^{2 i \pi \root m \of {1-\textsf{e}^{-\left( \mathscr {L}\left( -ln \left( 1-\mathscr {D}_{\mathscr {B}_{\mathscr {S}_2}}^{m}\right) \right) ^\mathscr {I}\right) ^{\frac{1}{\mathscr {I}}}}}} \end{array}\right) = \mathscr {S}_1^{\mathscr {L}} \boxtimes \mathscr {S}_2^{\mathscr {L}}$$

$$\square$$

### Theorem 3.7

Let $\mathscr {S} =(\mathscr {A}_{\mathscr {S}} \cdot e^{i.2 \pi \mathscr {D}_{\mathscr {A}_{\mathscr {S}}}}, \mathscr {B}_{\mathscr {S}} \cdot e^{i \cdot 2 \pi \mathscr {D}_{\mathscr {B}_{\mathscr {S}}}})$ be a Cn,m-ROFN, and $\mathscr {L}, \mathscr {L}_{1}, \mathscr {L}_{2}>0$, Then,

1. $$(\mathscr {L}_{1}+\mathscr {L}_{2}) \mathscr {S}=\mathscr {L}_{1} \mathscr {S} \boxplus \mathscr {L}_{2} \mathscr {S}$$
2. $$\mathscr {S}^{\mathscr {L}_{1}+\mathscr {L}_{2}}=\mathscr {S}^{\mathscr {L}_{1}} \boxtimes \mathscr {S}^{\mathscr {L}_{2}}$$
3. $$\mathscr {L}(\mathscr {S}^{c})=(\mathscr {S}^{\mathscr {L}})^{c}$$
4. $$(\mathscr {S}^{c})^{\mathscr {L}}=(\mathscr {L} \mathscr {S})^{c}$$

### Proof

1. $$(\mathscr {L}_{1}+\mathscr {L}_{2}) \mathscr {S}= \left( \begin{array}{c} \root n \of {1-\textsf{e}^{-\left( (\mathscr {L}_{1}+\mathscr {L}_{2})\left( -ln \left( 1-\mathscr {A}_{\mathscr {S}}^{n}\right) \right) ^\mathscr {I}\right) ^{\frac{1}{\mathscr {I}}}}}\\ \textsf{e}^{2 i \pi \root n \of {1-\textsf{e}^{-\left( (\mathscr {L}_{1}+\mathscr {L}_{2})\left( -ln \left( 1-\mathscr {D}_{\mathscr {A}_{\mathscr {S}}}^{n}\right) \right) ^\mathscr {I}\right) ^{\frac{1}{\mathscr {I}}}}}},\\ \root m \of {\textsf{e}^{-\left( (\mathscr {L}_{1}+\mathscr {L}_{2})\left( -ln \mathscr {B}_{\mathscr {S}}^{m}\right) ^\mathscr {I}\right) ^{\frac{1}{\mathscr {I}}}}}\\ \textsf{e}^{2 i \pi \root m \of {\textsf{e}^{-\left( (\mathscr {L}_{1}+\mathscr {L}_{2})\left( -ln \mathscr {D}_{\mathscr {B}_{\mathscr {S}}}^{m}\right) ^\mathscr {I}\right) ^{\frac{1}{\mathscr {I}}}}}} \end{array}\right) =$$
$$\left( \begin{array}{c} \root n \of {1-\textsf{e}^{-\left( \mathscr {L}_{1}\left( -ln \left( 1-\mathscr {A}_{\mathscr {S}}^{n}\right) \right) ^\mathscr {I}\right) ^{\frac{1}{\mathscr {I}}}}}\\ \textsf{e}^{2 i \pi \root n \of {1-\textsf{e}^{-\left( \mathscr {L}_{1}\left( -ln \left( 1-\mathscr {D}_{\mathscr {A}_{\mathscr {S}}}^{n}\right) \right) ^\mathscr {I}\right) ^{\frac{1}{\mathscr {I}}}}}},\\ \root m \of {\textsf{e}^{-\left( \mathscr {L}_{1}\left( -ln \mathscr {B}_{\mathscr {S}}^{m}\right) ^\mathscr {I}\right) ^{\frac{1}{\mathscr {I}}}}}\\ \textsf{e}^{2 i \pi \root m \of {\textsf{e}^{-\left( \mathscr {L}_{1}\left( -ln \mathscr {D}_{\mathscr {B}_{\mathscr {S}}}^{m}\right) ^\mathscr {I}\right) ^{\frac{1}{\mathscr {I}}}}}} \end{array}\right) \boxplus \left( \begin{array}{c} \root n \of {1-\textsf{e}^{-\left( \mathscr {L}_{2}\left( -ln \left( 1-\mathscr {A}_{\mathscr {S}}^{n}\right) \right) ^\mathscr {I}\right) ^{\frac{1}{\mathscr {I}}}}}\\ \textsf{e}^{2 i \pi \root n \of {1-\textsf{e}^{-\left( \mathscr {L}_{2}\left( -ln \left( 1-\mathscr {D}_{\mathscr {A}_{\mathscr {S}}}^{n}\right) \right) ^\mathscr {I}\right) ^{\frac{1}{\mathscr {I}}}}}},\\ \root m \of {\textsf{e}^{-\left( \mathscr {L}_{2}\left( -ln \mathscr {B}_{\mathscr {S}}^{m}\right) ^\mathscr {I}\right) ^{\frac{1}{\mathscr {I}}}}}\\ \textsf{e}^{2 i \pi \root m \of {\textsf{e}^{-\left( \mathscr {L}_{2}\left( -ln \mathscr {D}_{\mathscr {B}_{\mathscr {S}}}^{m}\right) ^\mathscr {I}\right) ^{\frac{1}{\mathscr {I}}}}}} \end{array}\right) =\mathscr {L}_{1} \mathscr {S} \boxplus \mathscr {L}_{2} \mathscr {S}$$
2. $$\mathscr {S}^{\mathscr {L}_{1}} \boxtimes \mathscr {S}^{\mathscr {L}_{2}} =\left( \begin{array}{c} \root n \of {\textsf{e}^{-\left( \mathscr {L}_{1}\left( -ln \mathscr {A}_{\mathscr {S}}^{n}\right) ^\mathscr {I}\right) ^{\frac{1}{\mathscr {I}}}}}\\ \textsf{e}^{2 i \pi \root n \of {\textsf{e}^{-\left( \mathscr {L}_{1}\left( -ln \mathscr {D}_{\mathscr {A}_{\mathscr {S}}}^{n}\right) ^\mathscr {I}\right) ^{\frac{1}{\mathscr {I}}}}}},\\ \root m \of {1-\textsf{e}^{-\left( \mathscr {L}_{1}\left( -ln \left( 1-\mathscr {B}_{\mathscr {S}}^{m}\right) \right) ^\mathscr {I}\right) ^{\frac{1}{\mathscr {I}}}}}\\ \textsf{e}^{2 i \pi \root m \of {1-\textsf{e}^{-\left( \mathscr {L}_{1}\left( -ln \left( 1-\mathscr {D}_{\mathscr {B}_{\mathscr {S}}}^{m}\right) \right) ^\mathscr {I}\right) ^{\frac{1}{\mathscr {I}}}}}} \end{array}\right) \boxtimes \left( \begin{array}{c} \root n \of {\textsf{e}^{-\left( \mathscr {L}_{2}\left( -ln \mathscr {A}_{\mathscr {S}}^{n}\right) ^\mathscr {I}\right) ^{\frac{1}{\mathscr {I}}}}}\\ \textsf{e}^{2 i \pi \root n \of {\textsf{e}^{-\left( \mathscr {L}_{2}\left( -ln \mathscr {D}_{\mathscr {A}_{\mathscr {S}}}^{n}\right) ^\mathscr {I}\right) ^{\frac{1}{\mathscr {I}}}}}},\\ \root m \of {1-\textsf{e}^{-\left( \mathscr {L}_{2}\left( -ln \left( 1-\mathscr {B}_{\mathscr {S}}^{m}\right) \right) ^\mathscr {I}\right) ^{\frac{1}{\mathscr {I}}}}}\\ \textsf{e}^{2 i \pi \root m \of {1-\textsf{e}^{-\left( \mathscr {L}_{2}\left( -ln \left( 1-\mathscr {D}_{\mathscr {B}_{\mathscr {S}}}^{m}\right) \right) ^\mathscr {I}\right) ^{\frac{1}{\mathscr {I}}}}}} \end{array}\right)=$$
$$\left( \begin{array}{c} \root n \of {\textsf{e}^{-\left( ({\mathscr {L}_{1}+\mathscr {L}_{2}})\left( -ln \mathscr {A}_{\mathscr {S}}^{n}\right) ^\mathscr {I}\right) ^{\frac{1}{\mathscr {I}}}}}\\ \textsf{e}^{2 i \pi \root n \of {\textsf{e}^{-\left( ({\mathscr {L}_{1}+\mathscr {L}_{2}})\left( -ln \mathscr {D}_{\mathscr {A}_{\mathscr {S}}}^{n}\right) ^\mathscr {I}\right) ^{\frac{1}{\mathscr {I}}}}}},\\ \root m \of {1-\textsf{e}^{-\left( ({\mathscr {L}_{1}+\mathscr {L}_{2}})\left( -ln \left( 1-\mathscr {B}_{\mathscr {S}}^{m}\right) \right) ^\mathscr {I}\right) ^{\frac{1}{\mathscr {I}}}}}\\ \textsf{e}^{2 i \pi \root m \of {1-\textsf{e}^{-\left( ({\mathscr {L}_{1}+\mathscr {L}_{2}})\left( -ln \left( 1-\mathscr {D}_{\mathscr {B}_{\mathscr {S}}}^{m}\right) \right) ^\mathscr {I}\right) ^{\frac{1}{\mathscr {I}}}}}} \end{array}\right) =\mathscr {S}^{\mathscr {L}_{1}+\mathscr {L}_{2}}$$
3. $$\mathscr {L}(\mathscr {S}^{c})=\mathscr {L}(\mathscr {B}_{\mathscr {S}_1}^{\frac{m}{n}} \cdot e^{i .2 \pi \mathscr {D}_{\mathscr {B}_{\mathscr {S}_1}}^{\frac{m}{n}}}, \mathscr {A}_{\mathscr {S}_1}^{\frac{n}{m}} \cdot e^{i \cdot 2 \pi \mathscr {D}_{\mathscr {A}_{\mathscr {S}_1}}^{\frac{n}{m}}}) =\left( \begin{array}{c} \root n \of {1-\textsf{e}^{-\left( \mathscr {L}\left( -ln \left( 1-\mathscr {B}_{\mathscr {S}_1}^{m}\right) \right) ^\mathscr {I}\right) ^{\frac{1}{\mathscr {I}}}}}\\ \textsf{e}^{2 i \pi \root n \of {1-\textsf{e}^{-\left( \mathscr {L}\left( -ln \left( 1-\mathscr {D}_{\mathscr {B}_{\mathscr {S}_1}}^{m}\right) \right) ^\mathscr {I}\right) ^{\frac{1}{\mathscr {I}}}}}},\\ \root m \of {\textsf{e}^{-\left( \mathscr {L}\left( -ln \mathscr {A}_{\mathscr {S}_1}^{n}\right) ^\mathscr {I}\right) ^{\frac{1}{\mathscr {I}}}}}\\ \textsf{e}^{2 i \pi \root m \of {\textsf{e}^{-\left( \mathscr {L}\left( -ln \mathscr {D}_{\mathscr {A}_{\mathscr {S}_1}}^{n}\right) ^\mathscr {I}\right) ^{\frac{1}{\mathscr {I}}}}}} \end{array}\right)=$$
$$\left( \begin{array}{c} \root n \of {\textsf{e}^{-\left( \mathscr {L}\left( -ln \mathscr {A}_{\mathscr {S}}^{n}\right) ^\mathscr {I}\right) ^{\frac{1}{\mathscr {I}}}}}\\ \textsf{e}^{2 i \pi \root n \of {\textsf{e}^{-\left( \mathscr {L}\left( -ln \mathscr {D}_{\mathscr {A}_{\mathscr {S}}}^{n}\right) ^\mathscr {I}\right) ^{\frac{1}{\mathscr {I}}}}}},\\ \root m \of {1-\textsf{e}^{-\left( \mathscr {L}\left( -ln \left( 1-\mathscr {B}_{\mathscr {S}}^{m}\right) \right) ^\mathscr {I}\right) ^{\frac{1}{\mathscr {I}}}}}\\ \textsf{e}^{2 i \pi \root m \of {1-\textsf{e}^{-\left( \mathscr {L}\left( -ln \left( 1-\mathscr {D}_{\mathscr {B}_{\mathscr {S}}}^{m}\right) \right) ^\mathscr {I}\right) ^{\frac{1}{\mathscr {I}}}}}} \end{array}\right) ^{c} =(\mathscr {S}^{\mathscr {L}})^{c}$$
4. $$(\mathscr {S}^{c})^{\mathscr {L}} =(\mathscr {B}_{\mathscr {S}_1}^{\frac{m}{n}} \cdot e^{i .2 \pi \mathscr {D}_{\mathscr {B}_{\mathscr {S}_1}}^{\frac{m}{n}}}, \mathscr {A}_{\mathscr {S}_1}^{\frac{n}{m}} \cdot e^{i \cdot 2 \pi \mathscr {D}_{\mathscr {A}_{\mathscr {S}_1}}^{\frac{n}{m}}})^{\mathscr {L}} =$$
$$\left( \begin{array}{c} \root n \of {\textsf{e}^{-\left( \mathscr {L}\left( -ln \mathscr {B}_{\mathscr {S}_1}^{m}\right) ^\mathscr {I}\right) ^{\frac{1}{\mathscr {I}}}}}\\ \textsf{e}^{2 i \pi \root n \of {\textsf{e}^{-\left( \mathscr {L}\left( -ln \mathscr {D}_{\mathscr {B}_{\mathscr {S}_1}}^{m}\right) ^\mathscr {I}\right) ^{\frac{1}{\mathscr {I}}}}}},\\ \root m \of {1-\textsf{e}^{-\left( \mathscr {L}\left( -ln \left( 1-dot{A}_{\mathscr {S}_1}^{n}\right) \right) ^\mathscr {I}\right) ^{\frac{1}{\mathscr {I}}}}}\\ \textsf{e}^{2 i \pi \root m \of {1-\textsf{e}^{-\left( \mathscr {L}\left( -ln \left( 1-\mathscr {D}_{\mathscr {A}_{\mathscr {S}_1}}^{n}\right) \right) ^\mathscr {I}\right) ^{\frac{1}{\mathscr {I}}}}}} \end{array}\right) =$$
$$\left( \begin{array}{c} \root n \of {1-\textsf{e}^{-\left( \mathscr {L}\left( -ln \left( 1-\mathscr {A}_{\mathscr {S}}^{n}\right) \right) ^\mathscr {I}\right) ^{\frac{1}{\mathscr {I}}}}}\\ \textsf{e}^{2 i \pi \root n \of {1-\textsf{e}^{-\left( \mathscr {L}\left( -ln \left( 1-\mathscr {D}_{\mathscr {A}_{\mathscr {S}}}^{n}\right) \right) ^\mathscr {I}\right) ^{\frac{1}{\mathscr {I}}}}}},\\ \root m \of {\textsf{e}^{-\left( \mathscr {L}\left( -ln \mathscr {B}_{\mathscr {S}}^{m}\right) ^\mathscr {I}\right) ^{\frac{1}{\mathscr {I}}}}}\\ \textsf{e}^{2 i \pi \root m \of {\textsf{e}^{-\left( \mathscr {L}\left( -ln \mathscr {D}_{\mathscr {B}_{\mathscr {S}}}^{m}\right) ^\mathscr {I}\right) ^{\frac{1}{\mathscr {I}}}}}} \end{array}\right) ^{c} =(\mathscr {L} \mathscr {S})^{c}$$

$$\square$$

### Theorem 3.8

Let $\mathscr {S}_1\!=\!(\mathscr {A}_{\mathscr {S}_1}\! \cdot\! e^{i.2 \pi \mathscr {D}_{\mathscr {A}_{\mathscr {S}_1}}}, \mathscr {B}_{\mathscr {S}_1} \!\cdot \!e^{i \cdot 2 \pi \mathscr {D}_{\mathscr {B}_{\mathscr {S}_1}}})$ and $\mathscr {S}_2\!=\!(\mathscr {A}_{\mathscr {S}_2}\! \cdot\! e^{i.2 \pi \mathscr {D}_{\mathscr {A}_{\mathscr {S}_2}}}, \mathscr {B}_{\mathscr {S}_2}\! \cdot\! e^{i \cdot 2 \pi \mathscr {D}_{\mathscr {B}_{\mathscr {S}_2}}})$ be two Cn,m-ROFNs. Then,

1. $$(\mathscr {S}_{1} \boxtimes \mathscr {S}_{2})^{c}=\mathscr {S}_{1}^{c} \boxplus \mathscr {S}_{2}^{c}$$
2. $$(\mathscr {S}_{1} \boxplus \mathscr {S}_{2})^{c}=\mathscr {S}_{1}^{c} \boxtimes \mathscr {S}_{2}^{c}$$

### Proof

1. $$(\mathscr {S}_{1} \boxtimes \mathscr {S}_{2})^{c}= \left( \begin{array}{c} \root n \of {\textsf{e}^{-\left( \left( -ln \mathscr {A}_{\mathscr {S}_1}^{n}\right) ^\mathscr {I}+\left( -ln \mathscr {A}_{\mathscr {S}_2}^{n}\right) ^\mathscr {I}\right) ^{\frac{1}{\mathscr {I}}}}}\\ \textsf{e}^{2 i \pi \root n \of {\textsf{e}^{-\left( \left( -ln \mathscr {D}_{\mathscr {A}_{\mathscr {S}_1}}^{n}\right) ^\mathscr {I}+\left( -ln \mathscr {D}_{\mathscr {A}_{\mathscr {S}_2}}^{n}\right) ^\mathscr {I}\right) ^{\frac{1}{\mathscr {I}}}}}},\\ \root m \of {1-\textsf{e}^{-\left( \left( -ln \left( 1-\mathscr {B}_{\mathscr {S}_1}^{m}\right) \right) ^\mathscr {I}+\left( -ln \left( 1-\mathscr {B}_{\mathscr {S}_2}^{m}\right) \right) ^\mathscr {I}\right) ^{\frac{1}{\mathscr {I}}}}}\\ \textsf{e}^{2 i \pi \root m \of {1-\textsf{e}^{-\left( \left( -ln \left( 1-\mathscr {D}_{\mathscr {B}_{\mathscr {S}_1}}^{m}\right) \right) ^\mathscr {I}+\left( -ln \left( 1-\mathscr {D}_{\mathscr {B}_{\mathscr {S}_2}}^{m}\right) \right) ^\mathscr {I}\right) ^{\frac{1}{\mathscr {I}}}}}} \end{array}\right) ^{c} =$$
$$\left( \begin{array}{c} \root n \of {1-\textsf{e}^{-\left( \left( -ln \left( 1-\mathscr {B}_{\mathscr {S}_1}^{m}\right) \right) ^\mathscr {I}+\left( -ln \left( 1-\mathscr {B}_{\mathscr {S}_2}^{m}\right) \right) ^\mathscr {I}\right) ^{\frac{1}{\mathscr {I}}}}}\\ \textsf{e}^{2 i \pi \root n \of {1-\textsf{e}^{-\left( \left( -ln \left( 1-\mathscr {D}_{\mathscr {B}_{\mathscr {S}_1}}^{m}\right) \right) ^\mathscr {I}+\left( -ln \left( 1-\mathscr {D}_{\mathscr {B}_{\mathscr {S}_2}}^{m}\right) \right) ^\mathscr {I}\right) ^{\frac{1}{\mathscr {I}}}}}},\\ \root m \of {\textsf{e}^{-\left( \left( -ln \mathscr {A}_{\mathscr {S}_1}^{n}\right) ^\mathscr {I}+\left( -ln \mathscr {A}_{\mathscr {S}_2}^{n}\right) ^\mathscr {I}\right) ^{\frac{1}{\mathscr {I}}}}}\\ \textsf{e}^{2 i \pi \root m \of {\textsf{e}^{-\left( \left( -ln \mathscr {D}_{\mathscr {A}_{\mathscr {S}_1}}^{n}\right) ^\mathscr {I}+\left( -ln \mathscr {D}_{\mathscr {A}_{\mathscr {S}_2}}^{n}\right) ^\mathscr {I}\right) ^{\frac{1}{\mathscr {I}}}}}} \end{array}\right) =$$
$$(\mathscr {B}_{\mathscr {S}_1}^{\frac{m}{n}} \cdot e^{i .2 \pi \mathscr {D}_{\mathscr {B}_{\mathscr {S}_1}}^{\frac{m}{n}}}, \mathscr {A}_{\mathscr {S}_1}^{\frac{n}{m}} \cdot e^{i \cdot 2 \pi \mathscr {D}_{\mathscr {A}_{\mathscr {S}_1}}^{\frac{n}{m}}}) \boxplus$$
$$(\mathscr {B}_{\mathscr {S}_2}^{\frac{m}{n}} \cdot e^{i .2 \pi \mathscr {D}_{\mathscr {B}_{\mathscr {S}_2}}^{\frac{m}{n}}}, \mathscr {A}_{\mathscr {S}_2}^{\frac{n}{m}} \cdot e^{i \cdot 2 \pi \mathscr {D}_{\mathscr {A}_{\mathscr {S}_2}}^{\frac{n}{m}}}) =\mathscr {S}_{1}^{c} \boxplus \mathscr {S}_{2}^{c}.$$
2. $$(\mathscr {S}_{1} \boxplus \mathscr {S}_{2})^{c} = \left( \begin{array}{c} \root n \of {1-\textsf{e}^{-\left( \left( -ln \left( 1-\mathscr {A}_{\mathscr {S}_1}^{n}\right) \right) ^\mathscr {I}+\left( -ln \left( 1-\mathscr {A}_{\mathscr {S}_2}^{n}\right) \right) ^\mathscr {I}\right) ^{\frac{1}{\mathscr {I}}}}}\\ \textsf{e}^{2 i \pi \root n \of {1-\textsf{e}^{-\left( \left( -ln \left( 1-\mathscr {D}_{\mathscr {A}_{\mathscr {S}_1}}^{n}\right) \right) ^\mathscr {I}+\left( -ln \left( 1-\mathscr {D}_{\mathscr {A}_{\mathscr {S}_2}}^{n}\right) \right) ^\mathscr {I}\right) ^{\frac{1}{\mathscr {I}}}}}},\\ \root m \of {\textsf{e}^{-\left( \left( -ln \mathscr {B}_{\mathscr {S}_1}^{m}\right) ^\mathscr {I}+\left( -ln \mathscr {B}_{\mathscr {S}_2}^{m}\right) ^\mathscr {I}\right) ^{\frac{1}{\mathscr {I}}}}}\\ \textsf{e}^{2 i \pi \root m \of {\textsf{e}^{-\left( \left( -ln \mathscr {D}_{\mathscr {B}_{\mathscr {S}_1}}^{m}\right) ^\mathscr {I}+\left( -ln \mathscr {D}_{\mathscr {B}_{\mathscr {S}_2}}^{m}\right) ^\mathscr {I}\right) ^{\frac{1}{\mathscr {I}}}}}} \end{array}\right) ^{c} =$$
$$\left( \begin{array}{c} \root n \of {\textsf{e}^{-\left( \left( -ln \mathscr {B}_{\mathscr {S}_1}^{m}\right) ^\mathscr {I}+\left( -ln \mathscr {B}_{\mathscr {S}_2}^{m}\right) ^\mathscr {I}\right) ^{\frac{1}{\mathscr {I}}}}}\\ \textsf{e}^{2 i \pi \root n \of {\textsf{e}^{-\left( \left( -ln \mathscr {D}_{\mathscr {B}_{\mathscr {S}_1}}^{m}\right) ^\mathscr {I}+\left( -ln \mathscr {D}_{\mathscr {B}_{\mathscr {S}_2}}^{m}\right) ^\mathscr {I}\right) ^{\frac{1}{\mathscr {I}}}}}},\\ \root m \of {1-\textsf{e}^{-\left( \left( -ln \left( 1-\mathscr {A}_{\mathscr {S}_1}^{n}\right) \right) ^\mathscr {I}+\left( -ln \left( 1-\mathscr {A}_{\mathscr {S}_2}^{n}\right) \right) ^\mathscr {I}\right) ^{\frac{1}{\mathscr {I}}}}}\\ \textsf{e}^{2 i \pi \root m \of {1-\textsf{e}^{-\left( \left( -ln \left( 1-\mathscr {D}_{\mathscr {A}_{\mathscr {S}_1}}^{n}\right) \right) ^\mathscr {I}+\left( -ln \left( 1-\mathscr {D}_{\mathscr {A}_{\mathscr {S}_2}}^{n}\right) \right) ^\mathscr {I}\right) ^{\frac{1}{\mathscr {I}}}}}} \end{array}\right) =$$
$$(\mathscr {B}_{\mathscr {S}_1}^{\frac{m}{n}} \cdot e^{i .2 \pi \mathscr {D}_{\mathscr {B}_{\mathscr {S}_1}}^{\frac{m}{n}}}, \mathscr {A}_{\mathscr {S}_1}^{\frac{n}{m}} \cdot e^{i \cdot 2 \pi \mathscr {D}_{\mathscr {A}_{\mathscr {S}_1}}^{\frac{n}{m}}}) \boxtimes (\mathscr {B}_{\mathscr {S}_1}^{\frac{m}{n}} \cdot e^{i .2 \pi \mathscr {D}_{\mathscr {B}_{\mathscr {S}_1}}^{\frac{m}{n}}}, \mathscr {A}_{\mathscr {S}_1}^{\frac{n}{m}} \cdot e^{i \cdot 2 \pi \mathscr {D}_{\mathscr {A}_{\mathscr {S}_1}}^{\frac{n}{m}}}) =\mathscr {S}_{1}^{c} \boxtimes \mathscr {S}_{2}^{c}.$$

$$\square$$

## Cn,m-ROF aczel-alsina weighted average and geometric aggregation operators

This section focuses on the application of aczel-alsina-based weighted average and geometric aggregation operators for processing data within the Cn,m-ROFS framework. The methodology is presented in detail, highlighting both the mathematical formulations and their practical significance.

### Definition 4.1

Let $\mathscr {K}=\left( \mathscr {K}_{1}, \mathscr {K}_{2}, \ldots , \mathscr {K}_{\rho }\right) ^{T}$ represent a vector of weights, where each weight satisfies $\mathscr {K}_{j}>0$ for all *j* and the condition ${\sum }_{j=1}^{\rho } \mathscr {K}_{j}=1$. Additionally, let $\mathscr {S}_{j}=(\mathscr {A}_{\mathscr {S}_j} \cdot e^{i.2 \pi \mathscr {D}_{\mathscr {A}_{\mathscr {S}_j}}}, \mathscr {B}_{\mathscr {S}_j} \cdot e^{i \cdot 2 \pi \mathscr {D}_{\mathscr {B}_{\mathscr {S}_j}}})$ denote a Cn,m-ROFNs for each $j=1, \ldots , \rho$ Then:

1. The Cn,m-ROFAAWA mapping is defined as Cn,m-ROFAAWA$: \mathscr {S}^{\rho } \rightarrow \mathscr {S}$, and is given by: 5
2. The Cn,m-ROFAAWG mapping is defined as Cn,m-ROFAAWG$: \mathscr {S}^{\rho } \rightarrow \mathscr {S}$, and is given by: 6

### Theorem 4.2

Let $\mathscr {K}=\left( \mathscr {K}_{1}, \mathscr {K}_{2}, \ldots , \mathscr {K}_{\rho }\right) ^{T}$ represent a vector of weights, where each weight satisfies $\mathscr {K}_{j}>0$ for all *j* and the requirement ${\sum }_{j=1}^{\rho } \mathscr {K}_{j}=1$. Additionally, let $\mathscr {S}_{j}=(\mathscr {A}_{\mathscr {S}_j} \cdot e^{i.2 \pi \mathscr {D}_{\mathscr {A}_{\mathscr {S}_j}}}, \mathscr {B}_{\mathscr {S}_j} \cdot e^{i \cdot 2 \pi \mathscr {D}_{\mathscr {B}_{\mathscr {S}_j}}})$ denote a Cn,m-ROFNs for every $j=1, \ldots , \rho$. Then, alternative formulations of the Cn,m-ROFAAWA and Cn,m-ROFAAWG methods are given as follows:

1. The Cn,m-ROFAAWA operator is expressed as:
  7 $$\begin{aligned} \text{Cn,m-ROFAAWA}\left( \mathscr {S}_{1}, \ldots , \mathscr {S}_{\rho }\right) =\left( \begin{array}{c} \root n \of {1-\textsf{e}^{-\left( \sum _{j=1}^{\rho } \mathscr {K}_{j}\left( -ln \left( 1-\mathscr {A}_{\mathscr {S}_j}^{n}\right) \right) ^\mathscr {I}\right) ^{\frac{1}{\mathscr {I}}}}}\\ \textsf{e}^{2 i \pi \root n \of {1-\textsf{e}^{-\left( \sum _{j=1}^{\rho } \mathscr {K}_{j}\left( -ln \left( 1-\mathscr {D}_{\mathscr {A}_{\mathscr {S}_j}}^{n}\right) \right) ^\mathscr {I}\right) ^{\frac{1}{\mathscr {I}}}}}},\\ \root m \of {\textsf{e}^{-\left( \sum _{j=1}^{\rho } \mathscr {K}_{j}\left( -ln \mathscr {B}_{\mathscr {S}_j}^{m}\right) ^\mathscr {I}\right) ^{\frac{1}{\mathscr {I}}}}}\\ \textsf{e}^{2 i \pi \root m \of {\textsf{e}^{-\left( \sum _{j=1}^{\rho } \mathscr {K}_{j}\left( -ln \mathscr {D}_{\mathscr {B}_{\mathscr {S}_j}}^{m}\right) ^\mathscr {I}\right) ^{\frac{1}{\mathscr {I}}}}}} \end{array}\right) \end{aligned}$$
2. The Cn,m-ROFAAWG operator is expressed as:
  8 $$\begin{aligned} \operatorname {Cn,m-ROFAAWG}\left( \mathscr {S}_{1}, \ldots , \mathscr {S}_{\rho }\right) =\left( \begin{array}{c} \root n \of {\textsf{e}^{-\left( \sum _{j=1}^{\rho } \mathscr {K}_{j}\left( -ln \mathscr {A}_{\mathscr {S}_j}^{n}\right) ^\mathscr {I}\right) ^{\frac{1}{\mathscr {I}}}}}\\ \textsf{e}^{2 i \pi \root n \of {\textsf{e}^{-\left( \sum _{j=1}^{\rho } \mathscr {K}_{j}\left( -ln \mathscr {D}_{\mathscr {A}_{\mathscr {S}_j}}^{n}\right) ^\mathscr {I}\right) ^{\frac{1}{\mathscr {I}}}}}},\\ \root m \of {1-\textsf{e}^{-\left( \sum _{j=1}^{\rho } \mathscr {K}_{j}\left( -ln \left( 1-\mathscr {B}_{\mathscr {S}_j}^{m}\right) \right) ^\mathscr {I}\right) ^{\frac{1}{\mathscr {I}}}}}\\ \textsf{e}^{2 i \pi \root m \of {1-\textsf{e}^{-\left( \sum _{j=1}^{\rho } \mathscr {K}_{j}\left( -ln \left( 1-\mathscr {D}_{\mathscr {B}_{\mathscr {S}_j}}^{m}\right) \right) ^\mathscr {I}\right) ^{\frac{1}{\mathscr {I}}}}}} \end{array}\right) \end{aligned}$$

### Proof

1. To validate the formula using mathematical induction, we first consider the base case $$\rho = 2,$$ for which the expression simplifies to: $$\text{Cn,m-ROFAAWA}\left( \mathscr {S}_{1}, \mathscr {S}_{2}\right) =\mathscr {K}_{1} \mathscr {S}_{1} \boxplus \mathscr {K}_{2} \mathscr {S}_{2}$$ $$= \left( \begin{array}{c} \root n \of {1-\textsf{e}^{-\left( \mathscr {K}_{1}\left( -ln \left( 1-\mathscr {A}_{\mathscr {S}_1}^{n}\right) \right) ^\mathscr {I}\right) ^{\frac{1}{\mathscr {I}}}}}\\ \textsf{e}^{2 i \pi \root n \of {1-\textsf{e}^{-\left( \mathscr {K}_{1}\left( -ln \left( 1-\mathscr {D}_{\mathscr {A}_{\mathscr {S}_1}}^{n}\right) \right) ^\mathscr {I}\right) ^{\frac{1}{\mathscr {I}}}}}},\\ \root m \of {\textsf{e}^{-\left( \mathscr {K}_{1}\left( -ln \mathscr {B}_{\mathscr {S}_1}^{m}\right) ^\mathscr {I}\right) ^{\frac{1}{\mathscr {I}}}}}\\ \textsf{e}^{2 i \pi \root m \of {\textsf{e}^{-\left( \mathscr {K}_{1}\left( -ln \mathscr {D}_{\mathscr {B}_{\mathscr {S}_1}}^{m}\right) ^\mathscr {I}\right) ^{\frac{1}{\mathscr {I}}}}}} \end{array}\right) \boxplus \left( \begin{array}{c} \root n \of {1-\textsf{e}^{-\left( \mathscr {K}_{2}\left( -ln \left( 1-\mathscr {A}_{\mathscr {S}_2}^{n}\right) \right) ^\mathscr {I}\right) ^{\frac{1}{\mathscr {I}}}}}\\ \textsf{e}^{2 i \pi \root n \of {1-\textsf{e}^{-\left( \mathscr {K}_{2}\left( -ln \left( 1-\mathscr {D}_{\mathscr {A}_{\mathscr {S}_2}}^{n}\right) \right) ^\mathscr {I}\right) ^{\frac{1}{\mathscr {I}}}}}},\\ \root m \of {\textsf{e}^{-\left( \mathscr {K}_{2}\left( -ln \mathscr {B}_{\mathscr {S}_2}^{m}\right) ^\mathscr {I}\right) ^{\frac{1}{\mathscr {I}}}}}\\ \textsf{e}^{2 i \pi \root m \of {\textsf{e}^{-\left( \mathscr {K}_{2}\left( -ln \mathscr {D}_{\mathscr {B}_{\mathscr {S}_2}}^{m}\right) ^\mathscr {I}\right) ^{\frac{1}{\mathscr {I}}}}}} \end{array}\right)$$ $$=\left( \begin{array}{c} \root n \of {1-\textsf{e}^{-\left( \mathscr {K}_{1}\left( -ln \left( 1-\mathscr {A}_{\mathscr {S}_1}^{n}\right) \right) ^\mathscr {I} + \mathscr {K}_{2}\left( -ln \left( 1-\mathscr {A}_{\mathscr {S}_2}^{n}\right) \right) ^\mathscr {I}\right) ^{\frac{1}{\mathscr {I}}}}}\\ \textsf{e}^{2 i \pi \root n \of {1-\textsf{e}^{-\left( \mathscr {K}_{1}\left( -ln \left( 1-\mathscr {D}_{\mathscr {A}_{\mathscr {S}_1}}^{n}\right) \right) ^\mathscr {I} + \mathscr {K}_{2}\left( -ln \left( 1-\mathscr {D}_{\mathscr {A}_{\mathscr {S}_2}}^{n}\right) \right) ^\mathscr {I}\right) ^{\frac{1}{\mathscr {I}}}}}},\\ \root m \of {\textsf{e}^{-\left( \mathscr {K}_{1}\left( -ln \mathscr {B}_{\mathscr {S}_1}^{m}\right) ^\mathscr {I} + \mathscr {K}_{2}\left( -ln \mathscr {B}_{\mathscr {S}_2}^{m}\right) ^\mathscr {I}\right) ^{\frac{1}{\mathscr {I}}}}}\\ \textsf{e}^{2 i \pi \root m \of {\textsf{e}^{-\left( \mathscr {K}_{1}\left( -ln \mathscr {D}_{\mathscr {B}_{\mathscr {S}_1}}^{m}\right) ^\mathscr {I} + \mathscr {K}_{2}\left( -ln \mathscr {D}_{\mathscr {B}_{\mathscr {S}_2}}^{m}\right) ^\mathscr {I}\right) ^{\frac{1}{\mathscr {I}}}}}} \end{array}\right) = \left( \begin{array}{c} \root n \of {1-\textsf{e}^{-\left( \sum _{j=1}^{2} \mathscr {K}_{j}\left( -ln \left( 1-\mathscr {A}_{\mathscr {S}_j}^{n}\right) \right) ^\mathscr {I}\right) ^{\frac{1}{\mathscr {I}}}}}\\ \textsf{e}^{2 i \pi \root n \of {1-\textsf{e}^{-\left( \sum _{j=1}^{2} \mathscr {K}_{j}\left( -ln \left( 1-\mathscr {D}_{\mathscr {A}_{\mathscr {S}_j}}^{n}\right) \right) ^\mathscr {I}\right) ^{\frac{1}{\mathscr {I}}}}}},\\ \root m \of {\textsf{e}^{-\left( \sum _{j=1}^{2} \mathscr {K}_{j}\left( -ln \mathscr {B}_{\mathscr {S}_j}^{m}\right) ^\mathscr {I}\right) ^{\frac{1}{\mathscr {I}}}}}\\ \textsf{e}^{2 i \pi \root m \of {\textsf{e}^{-\left( \sum _{j=1}^{2} \mathscr {K}_{j}\left( -ln \mathscr {D}_{\mathscr {B}_{\mathscr {S}_j}}^{m}\right) ^\mathscr {I}\right) ^{\frac{1}{\mathscr {I}}}}}} \end{array}\right).$$ Assuming the statement is true for $$\rho =z$$, this means: $$\text{Cn,m-ROFAAWA}\left( \mathscr {S}_{1}, \ldots , \mathscr {S}_{z}\right) = {\mathscr {K}}_{1} \mathscr {S}_{1} \boxplus \mathscr {K}_{2} \mathscr {S}_{2} \boxplus \ldots \boxplus \mathscr {K}_{z} \mathscr {S}_{z}= \left( \begin{array}{c} \root n \of {1-\textsf{e}^{-\left( \sum _{j=1}^{z} \mathscr {K}_{j}\left( -ln \left( 1-\mathscr {A}_{\mathscr {S}_j}^{n}\right) \right) ^\mathscr {I}\right) ^{\frac{1}{\mathscr {I}}}}}\\ \textsf{e}^{2 i \pi \root n \of {1-\textsf{e}^{-\left( \sum _{j=1}^{z} \mathscr {K}_{j}\left( -ln \left( 1-\mathscr {D}_{\mathscr {A}_{\mathscr {S}_j}}^{n}\right) \right) ^\mathscr {I}\right) ^{\frac{1}{\mathscr {I}}}}}},\\ \root m \of {\textsf{e}^{-\left( \sum _{j=1}^{z} \mathscr {K}_{j}\left( -ln \mathscr {B}_{\mathscr {S}_j}^{m}\right) ^\mathscr {I}\right) ^{\frac{1}{\mathscr {I}}}}}\\ \textsf{e}^{2 i \pi \root m \of {\textsf{e}^{-\left( \sum _{j=1}^{z} \mathscr {K}_{j}\left( -ln \mathscr {D}_{\mathscr {B}_{\mathscr {S}_j}}^{m}\right) ^\mathscr {I}\right) ^{\frac{1}{\mathscr {I}}}}}} \end{array}\right).$$ To demonstrate that $$\rho =z+1$$ is true, consider the inductive hypothesis: For $$\rho =z+1$$, the result is: $$\text{Cn,m-ROFAAWA}\left( \mathscr {S}_{1}, \ldots , \mathscr {S}_{z+1}\right) = {\mathscr {K}}_{1} \mathscr {S}_{1} \boxplus \mathscr {K}_{2} \mathscr {S}_{2} \boxplus \ldots \boxplus \mathscr {K}_{z+1} \mathscr {S}_{z+1}=$$$$\left( \begin{array}{c} \root n \of {1-\textsf{e}^{-\left( \sum _{j=1}^{z} \mathscr {K}_{j}\left( -ln \left( 1-\mathscr {A}_{\mathscr {S}_j}^{n}\right) \right) ^\mathscr {I}\right) ^{\frac{1}{\mathscr {I}}}}}\\ \textsf{e}^{2 i \pi \root n \of {1-\textsf{e}^{-\left( \sum _{j=1}^{z} \mathscr {K}_{j}\left( -ln \left( 1-\mathscr {D}_{\mathscr {A}_{\mathscr {S}_j}}^{n}\right) \right) ^\mathscr {I}\right) ^{\frac{1}{\mathscr {I}}}}}},\\ \root m \of {\textsf{e}^{-\left( \sum _{j=1}^{z} \mathscr {K}_{j}\left( -ln \mathscr {B}_{\mathscr {S}_j}^{m}\right) ^\mathscr {I}\right) ^{\frac{1}{\mathscr {I}}}}}\\ \textsf{e}^{2 i \pi \root m \of {\textsf{e}^{-\left( \sum _{j=1}^{z} \mathscr {K}_{j}\left( -ln \mathscr {D}_{\mathscr {B}_{\mathscr {S}_j}}^{m}\right) ^\mathscr {I}\right) ^{\frac{1}{\mathscr {I}}}}}} \end{array}\right) \boxplus$$$$\left( \begin{array}{c} \root n \of {1-\textsf{e}^{-\left( \mathscr {K}_{{z+1}}\left( -ln \left( 1-\mathscr {A}_{\mathscr {S}_{z+1}}^{n}\right) \right) ^\mathscr {I}\right) ^{\frac{1}{\mathscr {I}}}}}\\ \textsf{e}^{2 i \pi \root n \of {1-\textsf{e}^{-\left( \mathscr {K}_{{z+1}}\left( -ln \left( 1-\mathscr {D}_{\mathscr {A}_{\mathscr {S}_{z+1}}}^{n}\right) \right) ^\mathscr {I}\right) ^{\frac{1}{\mathscr {I}}}}}},\\ \root m \of {\textsf{e}^{-\left( \mathscr {K}_{{z+1}}\left( -ln \mathscr {B}_{\mathscr {S}_{z+1}}^{m}\right) ^\mathscr {I}\right) ^{\frac{1}{\mathscr {I}}}}}\\ \textsf{e}^{2 i \pi \root m \of {\textsf{e}^{-\left( \mathscr {K}_{{z+1}}\left( -ln \mathscr {D}_{\mathscr {B}_{\mathscr {S}_{z+1}}}^{m}\right) ^\mathscr {I}\right) ^{\frac{1}{\mathscr {I}}}}}} \end{array}\right)$$ $$= \left( \begin{array}{c} \root n \of {1-\textsf{e}^{-\left( \sum _{j=1}^{z+1} \mathscr {K}_{j}\left( -ln \left( 1-\mathscr {A}_{\mathscr {S}_j}^{n}\right) \right) ^\mathscr {I}\right) ^{\frac{1}{\mathscr {I}}}}}\\ \textsf{e}^{2 i \pi \root n \of {1-\textsf{e}^{-\left( \sum _{j=1}^{z+1} \mathscr {K}_{j}\left( -ln \left( 1-\mathscr {D}_{\mathscr {A}_{\mathscr {S}_j}}^{n}\right) \right) ^\mathscr {I}\right) ^{\frac{1}{\mathscr {I}}}}}},\\ \root m \of {\textsf{e}^{-\left( \sum _{j=1}^{z+1} \mathscr {K}_{j}\left( -ln \mathscr {B}_{\mathscr {S}_j}^{m}\right) ^\mathscr {I}\right) ^{\frac{1}{\mathscr {I}}}}}\\ \textsf{e}^{2 i \pi \root m \of {\textsf{e}^{-\left( \sum _{j=1}^{z+1} \mathscr {K}_{j}\left( -ln \mathscr {D}_{\mathscr {B}_{\mathscr {S}_j}}^{m}\right) ^\mathscr {I}\right) ^{\frac{1}{\mathscr {I}}}}}} \end{array}\right)$$. This expression coincides with the formula for $$\rho = z+1$$, thereby completing the proof by induction.
2. The proof proceeds using a strategy analogous to that applied in establishing (1).

$$\square$$

### Theorem 4.3

The aggregation results of $\text{Cn,m-ROFAAWA}\left( \mathscr {S}_{1}, \ldots , \mathscr {S}_{\rho }\right)$ and $\operatorname {Cn,m-ROFAAWG}\left( \mathscr {S}_{1}, \ldots , \mathscr {S}_{\rho }\right)$ are also Cn,m-ROFNs.

### Proof

Theorems 3.3 and 3.4 guarantee that the outputs produced by the $\text{Cn,m-ROFAAWA}$ and $\operatorname {Cn,m-ROFAAWG}$ operators are valid Cn,m-ROFNs. $\square$

### Example 4.4

Given $\mathscr {S}_{1} = (0.27 \cdot e^{i.2 \pi (0.32)}, 0.31 \cdot e^{i.2 \pi (0.24)})$, $\mathscr {S}_{2} = (0.12 \cdot e^{i.2 \pi (0.42)}, 0.36 \cdot e^{i.2 \pi (0.43)})$, and $\mathscr {S}_{3} = (0.46 \cdot e^{i.2 \pi (0.37)}, 0.29 \cdot e^{i.2 \pi (0.11)})$, employing the weight vector $\mathscr {K} = (0.33,0.36,0.31)^{T}$ as listed. Then: 1- $$\text{Cn,m-ROFAAWA}\left( \mathscr {S}_{1}, \mathscr {S}_{2},\mathscr {S}_{3}\right) = \left( \begin{array}{c} \root n \of {1-\textsf{e}^{-\left( \sum _{j=1}^{3} \mathscr {K}_{j}\left( -ln \left( 1-\mathscr {A}_{\mathscr {S}_j}^{n}\right) \right) ^\mathscr {I}\right) ^{\frac{1}{\mathscr {I}}}}}\\ \textsf{e}^{2 i \pi \root n \of {1-\textsf{e}^{-\left( \sum _{j=1}^{3} \mathscr {K}_{j}\left( -ln \left( 1-\mathscr {D}_{\mathscr {A}_{\mathscr {S}_j}}^{n}\right) \right) ^\mathscr {I}\right) ^{\frac{1}{\mathscr {I}}}}}},\\ \root m \of {\textsf{e}^{-\left( \sum _{j=1}^{3} \mathscr {K}_{j}\left( -ln \mathscr {B}_{\mathscr {S}_j}^{m}\right) ^\mathscr {I}\right) ^{\frac{1}{\mathscr {I}}}}}\\ \textsf{e}^{2 i \pi \root m \of {\textsf{e}^{-\left( \sum _{j=1}^{3} \mathscr {K}_{j}\left( -ln \mathscr {D}_{\mathscr {B}_{\mathscr {S}_j}}^{m}\right) ^\mathscr {I}\right) ^{\frac{1}{\mathscr {I}}}}}} \end{array}\right)$$

$$\approx \left\{ \begin{array}{llllllllll} (0.3141 \cdot e^{i .2 \pi (0.3745)}, 0.3205 \cdot e^{i .2 \pi (0.2325)}) & \\ \hbox { for}\, n= 2, \mathscr {I} = 1 \, \hbox {and} \, m= 3 ,\\ (0.4037 \cdot e^{i .2 \pi (0.3875)}, 0.3171 \cdot e^{i .2 \pi (0.1777)}) & \\ \hbox { for} n= 2, \mathscr {I} = 4 \hbox {and} m= 3 ,\\ (0.4257 \cdot e^{i .2 \pi (0.3960)}, 0.3181 \cdot e^{i .2 \pi (0.1916)}) & \\ \hbox { for } \, n= 5, \mathscr {I} = 3 \, \hbox { and}\, m= 9 ,\\ (0.3991 \cdot e^{i .2 \pi (0.3863)}, 0.3193 \cdot e^{i .2 \pi (0.2098)}) & \\ \hbox { for}\, n= 4, \mathscr {I} = 2 \, \hbox {and} \, m= 3 . \end{array} \right.$$

2- $$\operatorname {Cn,m-ROFAAWG}\left( \mathscr {S}_{1}, \mathscr {S}_{2},\mathscr {S}_{3}\right) = \left( \begin{array}{c} \root n \of {\textsf{e}^{-\left( \sum _{j=1}^{3} \mathscr {K}_{j}\left( -ln \mathscr {A}_{\mathscr {S}_j}^{n}\right) ^\mathscr {I}\right) ^{\frac{1}{\mathscr {I}}}}}\\ \textsf{e}^{2 i \pi \root n \of {\textsf{e}^{-\left( \sum _{j=1}^{3} \mathscr {K}_{j}\left( -ln \mathscr {D}_{\mathscr {A}_{\mathscr {S}_j}}^{n}\right) ^\mathscr {I}\right) ^{\frac{1}{\mathscr {I}}}}}},\\ \root m \of {1-\textsf{e}^{-\left( \sum _{j=1}^{3} \mathscr {K}_{j}\left( -ln \left( 1-\mathscr {B}_{\mathscr {S}_j}^{m}\right) \right) ^\mathscr {I}\right) ^{\frac{1}{\mathscr {I}}}}}\\ \textsf{e}^{2 i \pi \root m \of {1-\textsf{e}^{-\left( \sum _{j=1}^{3} \mathscr {K}_{j}\left( -ln \left( 1-\mathscr {D}_{\mathscr {B}_{\mathscr {S}_j}}^{m}\right) \right) ^\mathscr {I}\right) ^{\frac{1}{\mathscr {I}}}}}} \end{array}\right)$$

$$\approx \left\{ \begin{array}{llllllllll} (0.2379 \cdot e^{i .2 \pi (0.3692)}, 0.3247 \cdot e^{i .2 \pi (0.3247)}) & \\ \hbox { for}\, n= 2, \mathscr {I} = 1 \, \hbox {and} \, m= 3,\\ (0.1826 \cdot e^{i .2 \pi (0.3623)}, 0.3364 \cdot e^{i .2 \pi (0.3961)}) & \\ \hbox { for}\, n= 2, \mathscr {I} = 4 \, \hbox {and} \, m= 3,\\ (0.1963 \cdot e^{i .2 \pi (0.3645)}, 0.3469 \cdot e^{i .2 \pi (0.4140)}) & \\ \hbox { for}\, n= 5, \mathscr {I} = 3 \, \hbox {and} \, m= 9,\\ (0.2144 \cdot e^{i .2 \pi (0.3668)}, 0.3289 \cdot e^{i .2 \pi (0.3662)}) & \\ \hbox { for}\, n= 4, \mathscr {I} = 2\, \hbox {and}\, m= 3. \end{array} \right.$$

### Theorem 4.5

(Monotonicity) Let $\left\{ \mathscr {S}_{j}=\left( \mathscr {A}_{\mathscr {S}_j} \cdot e^{i.2 \pi \mathscr {D}_{\mathscr {A}_{\mathscr {S}_j}}}, \mathscr {B}_{\mathscr {S}_j} \cdot e^{i \cdot 2 \pi \mathscr {D}_{\mathscr {B}_{\mathscr {S}_j}}}\right) \right\} _{j=1, \ldots , \rho }$ and

$\left\{ \widetilde{\mathscr {S}_{j}}=\left( \mathscr {A}_{\widetilde{\mathscr {S}_{j}}} \cdot e^{i.2 \pi \mathscr {D}_{\mathscr {A}_{\widetilde{\mathscr {S}_{j}}}}}, \mathscr {B}_{\widetilde{\mathscr {S}_{j}}} \cdot e^{i \cdot 2 \pi \mathscr {D}_{\mathscr {B}_{\widetilde{\mathscr {S}_{j}}}}}\right) \right\} _{j=1, \ldots , \rho }$ be two sets of $\rho$ Cn,m-ROFNs. If $\mathscr {S}_{j}\subseteq \widetilde{\mathscr {S}}_{j}$ for every *j*, then:

1. $$\text{Cn,m-ROFAAWA}\left( \mathscr {S}_{1}, \ldots , \mathscr {S}_{\rho }\right) \le \text{Cn,m-ROFAAWA}\left( \widetilde{\mathscr {S}}_{1}, \ldots , \widetilde{\mathscr {S}}_{\rho }\right)$$
2. Cn,m-ROFAAWG$$\left( \mathscr {S}_{1}, \ldots , \mathscr {S}_{\rho }\right) \le \operatorname {Cn,m-ROFAAWG}\left( \widetilde{\mathscr {S}}_{1}, \ldots , \widetilde{\mathscr {S}}_{\rho }\right)$$

### Proof

It suffices to demonstrate the first part, as the second part can be established using a similar argument. Since for all *j* we have the following relations: $\mathscr {A}_{\mathscr {S}_j}\le \mathscr {A}_{\widetilde{\mathscr {S}_{j}}}, \mathscr {B}_{\mathscr {S}_j}\ge \mathscr {B}_{\widetilde{\mathscr {S}_{j}}}$, $\mathscr {D}_{\mathscr {A}_{\mathscr {S}_j}} \le \mathscr {D}_{\mathscr {A}_{\widetilde{\mathscr {S}_{j}}}}$, and $\mathscr {D}_{\mathscr {B}_{\mathscr {S}_j}} \ge \mathscr {D}_{\mathscr {B}_{\widetilde{\mathscr {S}_{j}}}}$, we can establish the following inequalities:

$$\begin{aligned} 1-\mathscr {A}_{\mathscr {S}_j}^{n}\ge 1-\mathscr {A}_{\widetilde{\mathscr {S}_{j}}}^{n}, \end{aligned}$$

$$\begin{aligned} 1-\mathscr {D}_{\mathscr {A}_{\mathscr {S}_j}}^{n} \ge 1- \mathscr {D}_{\mathscr {A}_{\widetilde{\mathscr {S}_{j}}}}^{n} \end{aligned}$$

$$\begin{aligned} \mathscr {B}_{\mathscr {S}_j}^{m}\ge \mathscr {B}_{\widetilde{\mathscr {S}_{j}}}^{m}, \end{aligned}$$

and

$$\begin{aligned} \mathscr {D}_{\mathscr {B}_{\mathscr {S}_j}}^{m} \ge \mathscr {D}_{\mathscr {B}_{\widetilde{\mathscr {S}_{j}}}}^{m}. \end{aligned}$$

Thus, we can also infer that:

$$\begin{aligned} -ln \left( 1-\mathscr {A}_{\mathscr {S}_j}^{n}\right) \le -ln \left( 1-\mathscr {A}_{\widetilde{\mathscr {S}_{j}}}^{n}\right) , \end{aligned}$$

$$\begin{aligned} -ln \left( 1-\mathscr {D}_{\mathscr {A}_{\mathscr {S}_j}}^{n}\right) \le -ln \left( 1- \mathscr {D}_{\mathscr {A}_{\widetilde{\mathscr {S}_{j}}}}^{n}\right) \end{aligned}$$

$$\begin{aligned} -ln \left( \mathscr {B}_{\mathscr {S}_j}^{m}\right) \le -ln \left( \mathscr {B}_{\widetilde{\mathscr {S}_{j}}}^{m}\right) , \end{aligned}$$

and

$$\begin{aligned} -ln \left( \mathscr {D}_{\mathscr {B}_{\mathscr {S}_j}}^{m}\right) \le -ln \left( \mathscr {D}_{\mathscr {B}_{\widetilde{\mathscr {S}_{j}}}}^{m}\right) . \end{aligned}$$

These inequalities lead to the following:

$$\begin{aligned} \root n \of {1-\textsf{e}^{-\left( \sum _{j=1}^{\rho } \mathscr {K}_{j}\left( -ln \left( 1-\mathscr {A}_{\mathscr {S}_j}^{n}\right) \right) ^\mathscr {I}\right) ^{\frac{1}{\mathscr {I}}}}}\le \root n \of {1-\textsf{e}^{-\left( \sum _{j=1}^{\rho } \mathscr {K}_{j}\left( -ln \left( 1-\mathscr {A}_{\widetilde{\mathscr {S}_{j}}}^{n}\right) \right) ^\mathscr {I}\right) ^{\frac{1}{\mathscr {I}}}}}, \end{aligned}$$

$$\begin{aligned} \root n \of {1-\textsf{e}^{-\left( \sum _{j=1}^{\rho } \mathscr {K}_{j}\left( -ln \left( 1-\mathscr {D}_{\mathscr {A}_{\mathscr {S}_j}}^{n}\right) \right) ^\mathscr {I}\right) ^{\frac{1}{\mathscr {I}}}}}\le \root n \of {1-\textsf{e}^{-\left( \sum _{j=1}^{\rho } \mathscr {K}_{j}\left( -ln \left( 1-\mathscr {D}_{\mathscr {A}_{\widetilde{\mathscr {S}_{j}}}}^{n}\right) \right) ^\mathscr {I}\right) ^{\frac{1}{\mathscr {I}}}}}, \end{aligned}$$

$$\begin{aligned} \root m \of {\textsf{e}^{-\left( \sum _{j=1}^{\rho } \mathscr {K}_{j}\left( -ln \mathscr {B}_{\widetilde{\mathscr {S}_{j}}}^{m}\right) ^\mathscr {I}\right) ^{\frac{1}{\mathscr {I}}}}}\le \root m \of {\textsf{e}^{-\left( \sum _{j=1}^{\rho } \mathscr {K}_{j}\left( -ln \mathscr {B}_{\mathscr {S}_j}^{m}\right) ^\mathscr {I}\right) ^{\frac{1}{\mathscr {I}}}}}, \end{aligned}$$

and

$$\begin{aligned} \root m \of {\textsf{e}^{-\left( \sum _{j=1}^{\rho } \mathscr {K}_{j}\left( -ln \mathscr {D}_{\mathscr {B}_{\widetilde{\mathscr {S}_{j}}}}^{m}\right) ^\mathscr {I}\right) ^{\frac{1}{\mathscr {I}}}}}\le \root m \of {\textsf{e}^{-\left( \sum _{j=1}^{\rho } \mathscr {K}_{j}\left( -ln \mathscr {D}_{\mathscr {B}_{\mathscr {S}_j}}^{m}\right) ^\mathscr {I}\right) ^{\frac{1}{\mathscr {I}}}}}. \end{aligned}$$

Therefore, we can conclude that:$$\text{Cn,m-ROFAAWA}(\mathscr {S}_1, \mathscr {S}_2, ..., \mathscr {S}_{\rho })=$$$$\left( \begin{array}{c} \root n \of {1-\textsf{e}^{-\left( \sum _{j=1}^{\rho } \mathscr {K}_{j}\left( -ln \left( 1-\mathscr {A}_{\mathscr {S}_j}^{n}\right) \right) ^\mathscr {I}\right) ^{\frac{1}{\mathscr {I}}}}}\\ \textsf{e}^{2 i \pi \root n \of {1-\textsf{e}^{-\left( \sum _{j=1}^{\rho } \mathscr {K}_{j}\left( -ln \left( 1-\mathscr {D}_{\mathscr {A}_{\mathscr {S}_j}}^{n}\right) \right) ^\mathscr {I}\right) ^{\frac{1}{\mathscr {I}}}}}},\\ \root m \of {\textsf{e}^{-\left( \sum _{j=1}^{\rho } \mathscr {K}_{j}\left( -ln \mathscr {B}_{\mathscr {S}_j}^{m}\right) ^\mathscr {I}\right) ^{\frac{1}{\mathscr {I}}}}}\\ \textsf{e}^{2 i \pi \root m \of {\textsf{e}^{-\left( \sum _{j=1}^{\rho } \mathscr {K}_{j}\left( -ln \mathscr {D}_{\mathscr {B}_{\mathscr {S}_j}}^{m}\right) ^\mathscr {I}\right) ^{\frac{1}{\mathscr {I}}}}}} \end{array}\right) \le \left( \begin{array}{c} \root n \of {1-\textsf{e}^{-\left( \sum _{j=1}^{\rho } \mathscr {K}_{j}\left( -ln \left( 1-\mathscr {A}_{\widetilde{\mathscr {S}_{j}}}^{n}\right) \right) ^\mathscr {I}\right) ^{\frac{1}{\mathscr {I}}}}}\\ \textsf{e}^{2 i \pi \root n \of {1-\textsf{e}^{-\left( \sum _{j=1}^{\rho } \mathscr {K}_{j}\left( -ln \left( 1-\mathscr {D}_{\mathscr {A}_{\widetilde{\mathscr {S}_{j}}}}^{n}\right) \right) ^\mathscr {I}\right) ^{\frac{1}{\mathscr {I}}}}}},\\ \root m \of {\textsf{e}^{-\left( \sum _{j=1}^{\rho } \mathscr {K}_{j}\left( -ln \mathscr {B}_{\widetilde{\mathscr {S}_{j}}}^{m}\right) ^\mathscr {I}\right) ^{\frac{1}{\mathscr {I}}}}}\\ \textsf{e}^{2 i \pi \root m \of {\textsf{e}^{-\left( \sum _{j=1}^{\rho } \mathscr {K}_{j}\left( -ln \mathscr {D}_{\mathscr {B}_{\widetilde{\mathscr {S}_{j}}}}^{m}\right) ^\mathscr {I}\right) ^{\frac{1}{\mathscr {I}}}}}} \end{array}\right) =$$ $$\text{Cn,m-ROFAAWA}(\widetilde{\mathscr {S}}_1, \widetilde{\mathscr {S}}_2, ..., \widetilde{\mathscr {S}}_{\rho })$$. $$\square$$

### Theorem 4.6

(Boundedness) Let $\left\{ \mathscr {S}_{j}=\left( \mathscr {A}_{\mathscr {S}_j} \cdot e^{i.2 \pi \mathscr {D}_{\mathscr {A}_{\mathscr {S}_j}}}, \mathscr {B}_{\mathscr {S}_j} \cdot e^{i \cdot 2 \pi \mathscr {D}_{\mathscr {B}_{\mathscr {S}_j}}}\right) \right\} _{j=1, \ldots , \rho }$ represent a set of $\rho$ Cn,m-ROFNs. Let $\underline{\mathscr {S}}$ and $\overline{\mathscr {S}}$ be two Cn,m-ROFNs defined as:

$$\begin{aligned} \underline{\mathscr {S}}=({\mathscr {A}_{\underline{\mathscr {S}}}}^{-} \cdot e^{i .2 \pi {\mathscr {D}_{\mathscr {A}_{\underline{\mathscr {S}}}}}^{-}}, {\mathscr {B}_{\underline{\mathscr {S}}}}^{+} \cdot e^{i \cdot 2 \pi {\mathscr {D}_{\mathscr {B}_{\underline{\mathscr {S}}}}}^{+}})= \left( \min \left( \mathscr {A}_{\mathscr {S}_j}\right) \cdot e^{i .2 \pi \min \left( \mathscr {D}_{\mathscr {A}_{\mathscr {S}_j}}\right) }, \max \left( \mathscr {B}_{\mathscr {S}_j}\right) \cdot e^{i .2 \pi \max \left( \mathscr {D}_{\mathscr {B}_{\mathscr {S}_j}}\right) }\right) \end{aligned}$$

and

$$\begin{aligned} \overline{\mathscr {S}}=({\mathscr {A}_{\overline{\mathscr {S}}}}^{+} \cdot e^{i .2 \pi {\mathscr {D}_{\mathscr {A}_{\overline{\mathscr {S}}}}}^{+}}, {\mathscr {B}_{\overline{\mathscr {S}}}}^{-} \cdot e^{i \cdot 2 \pi {\mathscr {D}_{\mathscr {B}_{\overline{\mathscr {S}}}}}^{-}})= \left( \max \left( \mathscr {A}_{\mathscr {S}_j}\right) \cdot e^{i .2 \pi \max \left( \mathscr {D}_{\mathscr {A}_{\mathscr {S}_j}}\right) }, \min \left( \mathscr {B}_{\mathscr {S}_j}\right) \cdot e^{i .2 \pi \min \left( \mathscr {D}_{\mathscr {B}_{\mathscr {S}_j}}\right) }\right) . \end{aligned}$$

Then:

1. $$\underline{\mathscr {S}} \le \text{Cn,m-ROFAAWA}\left( \mathscr {S}_{1}, \ldots , \mathscr {S}_{\rho }\right) \le \overline{\mathscr {S}}$$
2. $$\underline{\mathscr {S}} \le \operatorname {Cn,m-ROFAAWG}\left( \mathscr {S}_{1}, \ldots , \mathscr {S}_{\rho }\right) \le \overline{\mathscr {S}}$$

### Proof

We focus on proving the first result, since the second can be established using a similar approach. For the first part, it suffices to demonstrate that

$$\begin{aligned} {\mathscr {A}_{\underline{\mathscr {S}}}}^{-}\le \root n \of {1-\textsf{e}^{-\left( \sum _{j=1}^{\rho } \mathscr {K}_{j}\left( -ln \left( 1-\mathscr {A}_{\mathscr {S}_j}^{n}\right) \right) ^\mathscr {I}\right) ^{\frac{1}{\mathscr {I}}}}} \le {\mathscr {A}_{\overline{\mathscr {S}}}}^{+}, \end{aligned}$$

$$\begin{aligned} {\mathscr {D}_{\mathscr {A}_{\underline{\mathscr {S}}}}}^{-}\le \root n \of {1-\textsf{e}^{-\left( \sum _{j=1}^{\rho } \mathscr {K}_{j}\left( -ln \left( 1-\mathscr {D}_{\mathscr {A}_{\mathscr {S}_j}}^{n}\right) \right) ^\mathscr {I}\right) ^{\frac{1}{\mathscr {I}}}}} \le {\mathscr {D}_{\mathscr {A}_{\overline{\mathscr {S}}}}}^{+}, \end{aligned}$$

$$\begin{aligned} {\mathscr {B}_{\underline{\mathscr {S}}}}^{+}\ge \root m \of {\textsf{e}^{-\left( \sum _{j=1}^{\rho } \mathscr {K}_{j}\left( -ln \mathscr {B}_{\mathscr {S}_j}^{m}\right) ^\mathscr {I}\right) ^{\frac{1}{\mathscr {I}}}}}\ge {\mathscr {B}_{\overline{\mathscr {S}}}}^{-}, \end{aligned}$$

and

$$\begin{aligned} {\mathscr {D}_{\mathscr {B}_{\underline{\mathscr {S}}}}}^{+}\ge \root m \of {\textsf{e}^{-\left( \sum _{j=1}^{\rho } \mathscr {K}_{j}\left( -ln \mathscr {D}_{\mathscr {B}_{\mathscr {S}_j}}^{m}\right) ^\mathscr {I}\right) ^{\frac{1}{\mathscr {I}}}}} \ge {\mathscr {D}_{\mathscr {B}_{\overline{\mathscr {S}}}}}^{-}. \end{aligned}$$

Since $({\mathscr {A}_{\underline{\mathscr {S}}}}^{-})^{n} \le \mathscr {A}_{\mathscr {S}_j}^{n} \le ({\mathscr {A}_{\overline{\mathscr {S}}}}^{+})^{n}$, we have

$$\begin{aligned} \sum _{j=1}^{\rho } \mathscr {K}_{j}\left( -ln \left( 1-({\mathscr {A}_{\underline{\mathscr {S}}}}^{-})^{n}\right) \right) ^\mathscr {I}&\le \sum _{j=1}^{\rho } \mathscr {K}_{j}\left( -ln \left( 1-\mathscr {A}_{\mathscr {S}_j}^{n}\right) \right) ^\mathscr {I} \le \\ \sum _{j=1}^{\rho } \mathscr {K}_{j}\left( -ln \left( 1-(\mathscr {A}_{\overline{\mathscr {S}}}^{+})^{n}\right) \right) ^\mathscr {I} \end{aligned}$$

and hence

$$\begin{aligned} \left( -ln \left( 1-({\mathscr {A}_{\underline{\mathscr {S}}}}^{-})^{n}\right) \right) ^\mathscr {I} \sum _{j=1}^{\rho } \mathscr {K}_{j}&\le \sum _{j=1}^{\rho } \mathscr {K}_{j}\left( -ln \left( 1-\mathscr {A}_{\mathscr {S}_j}^{n}\right) \right) ^\mathscr {I} \le \\ \left( -ln \left( 1-(\mathscr {A}_{\overline{\mathscr {S}}}^{+})^{n}\right) \right) ^\mathscr {I} \sum _{j=1}^{\rho } \mathscr {K}_{j} \end{aligned}$$

since $$\sum _{j}^{\rho } \mathscr {K}_{j} = 1$$, so

$$\begin{aligned} \left( -ln \left( 1-({\mathscr {A}_{\underline{\mathscr {S}}}}^{-})^{n}\right) \right) ^\mathscr {I}&\le \sum _{j=1}^{\rho } \mathscr {K}_{j}\left( -ln \left( 1-\mathscr {A}_{\mathscr {S}_j}^{n}\right) \right) ^\mathscr {I} \le \left( -ln \left( 1-(\mathscr {A}_{\overline{\mathscr {S}}}^{+})^{n}\right) \right) ^\mathscr {I} \end{aligned}$$

implies that

$$\begin{aligned} ln \left( 1-({\mathscr {A}_{\underline{\mathscr {S}}}}^{-})^{n}\right)&\ge -\left( \sum _{j=1}^{\rho } \mathscr {K}_{j}\left( -ln \left( 1-\mathscr {A}_{\mathscr {S}_j}^{n}\right) \right) ^\mathscr {I}\right) ^{\frac{1}{\mathscr {I}}} \ge ln \left( 1-(\mathscr {A}_{\overline{\mathscr {S}}}^{+})^{n}\right) \end{aligned}$$

and thus

$$\begin{aligned} 1-({\mathscr {A}_{\underline{\mathscr {S}}}}^{-})^{n}&\ge \textsf{e}^{-\left( \sum _{j=1}^{\rho } \mathscr {K}_{j}\left( -ln \left( 1-\mathscr {A}_{\mathscr {S}_j}^{n}\right) \right) ^\mathscr {I}\right) ^{\frac{1}{\mathscr {I}}}} \ge 1-(\mathscr {A}_{\overline{\mathscr {S}}}^{+})^{n} \end{aligned}$$

and therefore

$$\begin{aligned} {\mathscr {A}_{\underline{\mathscr {S}}}}^{-} \le \root n \of {1-\textsf{e}^{-\left( \sum _{j=1}^{\rho } \mathscr {K}_{j}\left( -ln \left( 1-\mathscr {A}_{\mathscr {S}_j}^{n}\right) \right) ^\mathscr {I}\right) ^{\frac{1}{\mathscr {I}}}}} \le {\mathscr {A}_{\overline{\mathscr {S}}}}^{+}. \end{aligned}$$

Similarly, we can illustrate

$$\begin{aligned} {\mathscr {D}_{\mathscr {A}_{\underline{\mathscr {S}}}}}^{-}\le \root n \of {1-\textsf{e}^{-\left( \sum _{j=1}^{\rho } \mathscr {K}_{j}\left( -ln \left( 1-\mathscr {D}_{\mathscr {A}_{\mathscr {S}_j}}^{n}\right) \right) ^\mathscr {I}\right) ^{\frac{1}{\mathscr {I}}}}} \le {\mathscr {D}_{\mathscr {A}_{\overline{\mathscr {S}}}}}^{+}, \end{aligned}$$

$$\begin{aligned} {\mathscr {B}_{\underline{\mathscr {S}}}}^{+}\ge \root m \of {\textsf{e}^{-\left( \sum _{j=1}^{\rho } \mathscr {K}_{j}\left( -ln \mathscr {B}_{\mathscr {S}_j}^{m}\right) ^\mathscr {I}\right) ^{\frac{1}{\mathscr {I}}}}}\ge {\mathscr {B}_{\overline{\mathscr {S}}}}^{-}, \end{aligned}$$

and

$$\begin{aligned} {\mathscr {D}_{\mathscr {B}_{\underline{\mathscr {S}}}}}^{+}\ge \root m \of {\textsf{e}^{-\left( \sum _{j=1}^{\rho } \mathscr {K}_{j}\left( -ln \mathscr {D}_{\mathscr {B}_{\mathscr {S}_j}}^{m}\right) ^\mathscr {I}\right) ^{\frac{1}{\mathscr {I}}}}} \ge {\mathscr {D}_{\mathscr {B}_{\overline{\mathscr {S}}}}}^{-}. \end{aligned}$$

$$\square$$

### Theorem 4.7

(Idempotency) Let $\left\{ \mathscr {S}_{j}=\left( \mathscr {A}_{\mathscr {S}_j} \cdot e^{i.2 \pi \mathscr {D}_{\mathscr {A}_{\mathscr {S}_j}}}, \mathscr {B}_{\mathscr {S}_j} \cdot e^{i \cdot 2 \pi \mathscr {D}_{\mathscr {B}_{\mathscr {S}_j}}}\right) \right\} _{j=1, \ldots , \rho }$ represent a set of $\rho$ Cn,m-ROFNs. If $\mathscr {S}_{j}=\mathscr {S}=(\mathscr {A}_{{\mathscr {S}}} \cdot e^{i.2 \pi \mathscr {D}_{\mathscr {A}_{{\mathscr {S}}}}}, \mathscr {B}_{{\mathscr {S}}} \cdot e^{i \cdot 2 \pi \mathscr {D}_{\mathscr {B}_{{\mathscr {S}}}}})$ is any Cn,m-ROFN and $\mathscr {K}=\left( \mathscr {K}_{1}, \mathscr {K}_{2}, \ldots , \mathscr {K}_{\rho }\right) ^{T}$ is a weight vector such that $\sum _{j=1}^{\rho } \mathscr {K}_{j}=1$ and $\mathscr {K}>0$, then:

1. $$\text{Cn,m-ROFAAWA}\left( \mathscr {S}_{1}, \ldots , \mathscr {S}_{\rho }\right) =\mathscr {S}$$
2. Cn,m-ROFAAWG$$\left( \mathscr {S}_{1}, \ldots , \mathscr {S}_{\rho }\right) =\mathscr {S}$$

### Proof

It is sufficient to prove only the first part, as the second follows by a similar argument. For all *j*, let $$\mathscr {S}_{j} = \mathscr {S}$$. This implies that the following holds: $$\text{Cn,m-ROFAAWA}(\mathscr {S}_1, \mathscr {S}_2, ..., \mathscr {S}_{\rho }) =\left( \begin{array}{c} \root n \of {1-\textsf{e}^{-\left( \sum _{j=1}^{\rho } \mathscr {K}_{j}\left( -ln \left( 1-\mathscr {A}_{\mathscr {S}_j}^{n}\right) \right) ^\mathscr {I}\right) ^{\frac{1}{\mathscr {I}}}}}\\ \textsf{e}^{2 i \pi \root n \of {1-\textsf{e}^{-\left( \sum _{j=1}^{\rho } \mathscr {K}_{j}\left( -ln \left( 1-\mathscr {D}_{\mathscr {A}_{\mathscr {S}_j}}^{n}\right) \right) ^\mathscr {I}\right) ^{\frac{1}{\mathscr {I}}}}}},\\ \root m \of {\textsf{e}^{-\left( \sum _{j=1}^{\rho } \mathscr {K}_{j}\left( -ln \mathscr {B}_{\mathscr {S}_j}^{m}\right) ^\mathscr {I}\right) ^{\frac{1}{\mathscr {I}}}}}\\ \textsf{e}^{2 i \pi \root m \of {\textsf{e}^{-\left( \sum _{j=1}^{\rho } \mathscr {K}_{j}\left( -ln \mathscr {D}_{\mathscr {B}_{\mathscr {S}_j}}^{m}\right) ^\mathscr {I}\right) ^{\frac{1}{\mathscr {I}}}}}} \end{array}\right) =$$$$\left( \begin{array}{c} \root n \of {1-\textsf{e}^{-\left( \left( -ln \left( 1-\mathscr {A}_{\mathscr {S}}^{n}\right) \right) ^\mathscr {I}\right) ^{\frac{1}{\mathscr {I}}}}}\\ \textsf{e}^{2 i \pi \root n \of {1-\textsf{e}^{-\left( \left( -ln \left( 1-\mathscr {D}_{\mathscr {A}_{\mathscr {S}}}^{n}\right) \right) ^\mathscr {I}\right) ^{\frac{1}{\mathscr {I}}}}}},\\ \root m \of {\textsf{e}^{-\left( \left( -ln \mathscr {B}_{\mathscr {S}}^{m}\right) ^\mathscr {I}\right) ^{\frac{1}{\mathscr {I}}}}}\\ \textsf{e}^{2 i \pi \root m \of {\textsf{e}^{-\left( \left( -ln \mathscr {D}_{\mathscr {B}_{\mathscr {S}}}^{m}\right) ^\mathscr {I}\right) ^{\frac{1}{\mathscr {I}}}}}} \end{array}\right) =(\mathscr {A}_{{\mathscr {S}}} \cdot e^{i .2 \pi \mathscr {D}_{\mathscr {A}_{{\mathscr {S}}}}}, \mathscr {B}_{{\mathscr {S}}} \cdot e^{i \cdot 2 \pi \mathscr {D}_{\mathscr {B}_{{\mathscr {S}}}}}) = \mathscr {S}$$. $$\square$$

### Theorem 4.8

Let $\left\{ \mathscr {S}_{j}=(\mathscr {A}_{\mathscr {S}_j} \cdot e^{i.2 \pi \mathscr {D}_{\mathscr {A}_{\mathscr {S}_j}}}, \mathscr {B}_{\mathscr {S}_j} \cdot e^{i \cdot 2 \pi \mathscr {D}_{\mathscr {B}_{\mathscr {S}_j}}})\right\} _{j=1, \ldots , \rho }$ indicate a set of $\rho$ Cn,m-ROFNs, and let $\mathscr {S}=(\mathscr {A}_{{\mathscr {S}}} \cdot e^{i.2 \pi \mathscr {D}_{\mathscr {A}_{{\mathscr {S}}}}}, \mathscr {B}_{{\mathscr {S}}} \cdot e^{i \cdot 2 \pi \mathscr {D}_{\mathscr {B}_{{\mathscr {S}}}}})$ be any Cn,m-ROFN. Suppose the weight vector $\mathscr {K}=\left( \mathscr {K}_{1}, \ldots , \mathscr {K}_{\rho }\right)$ with $\sum _{j=1}^{\rho } \mathscr {K}_{j}=1$, it follows that:

1. $$\text{Cn,m-ROFAAWA}\left( \mathscr {S}_{1} \boxplus \mathscr {S}, \ldots , \mathscr {S}_{\rho } \boxplus \mathscr {S}\right)$$

$$\ge \text{Cn,m-ROFAAWA}\left( \mathscr {S}_{1} \boxtimes \mathscr {S}, \ldots , \mathscr {S}_{\rho } \boxtimes \mathscr {S}\right)$$.

5. $$\operatorname {Cn,m-ROFAAWG}\left( \mathscr {S}_{1} \boxplus \mathscr {S}, \ldots , \mathscr {S}_{\rho } \boxplus \mathscr {S}\right)$$

$$\ge \operatorname {Cn,m-ROFAAWG}\left( \mathscr {S}_{1} \boxtimes \mathscr {S}, \ldots , \mathscr {S}_{\rho } \boxtimes \mathscr {S}\right)$$

### Proof

For any $\mathscr {S}_{j}=(\mathscr {A}_{\mathscr {S}_j} \cdot e^{i.2 \pi \mathscr {D}_{\mathscr {A}_{\mathscr {S}_j}}}, \mathscr {B}_{\mathscr {S}_j} \cdot e^{i \cdot 2 \pi \mathscr {D}_{\mathscr {B}_{\mathscr {S}_j}}})$ and $\mathscr {S}=(\mathscr {A}_{{\mathscr {S}}} \cdot e^{i.2 \pi \mathscr {D}_{\mathscr {A}_{{\mathscr {S}}}}}, \mathscr {B}_{{\mathscr {S}}} \cdot e^{i \cdot 2 \pi \mathscr {D}_{\mathscr {B}_{{\mathscr {S}}}}})$, we have $\mathscr {A}_{\mathscr {S}_j}^{n} \mathscr {A}_{{\mathscr {S}}}^{n}\le \mathscr {A}_{\mathscr {S}_j}^{n}$ and $\mathscr {A}_{\mathscr {S}_j}^{n} \mathscr {A}_{{\mathscr {S}}}^{n} \le \mathscr {A}_{{\mathscr {S}}}^{n}$, then

$$\begin{aligned} \mathscr {A}_{\mathscr {S}_j}^{n} \mathscr {A}_{\mathscr {S}}^{n} + \mathscr {A}_{\mathscr {S}}^{n} \mathscr {A}_{\mathscr {S}_j}^{n}\le \mathscr {A}_{\mathscr {S}}^{n} + \mathscr {A}_{\mathscr {S}_j}^{n} \end{aligned}$$

$$\begin{aligned} \Rightarrow \mathscr {A}_{\mathscr {S}_j}^{n} \mathscr {A}_{\mathscr {S}}^{n}\le \mathscr {A}_{\mathscr {S}}^{n} + \mathscr {A}_{\mathscr {S}_j}^{n} - \mathscr {A}_{\mathscr {S}}^{n} \mathscr {A}_{\mathscr {S}_j}^{n} \end{aligned}$$

$$\begin{aligned} \Rightarrow \mathscr {A}_{\mathscr {S}_j}^{n} \mathscr {A}_{\mathscr {S}}^{n}\le 1- \left( 1 - \mathscr {A}_{\mathscr {S}}^{n} - \mathscr {A}_{\mathscr {S}_j}^{n} + \mathscr {A}_{\mathscr {S}}^{n} \mathscr {A}_{\mathscr {S}_j}^{n}\right) \end{aligned}$$

$$\begin{aligned} \Rightarrow \mathscr {A}_{\mathscr {S}_j}^{n} \mathscr {A}_{\mathscr {S}}^{n}\le 1- \left( 1-\mathscr {A}_{\mathscr {S}_j}^{n}\right) \left( 1-\mathscr {A}_{\mathscr {S}}^{n}\right) \end{aligned}$$

$$\begin{aligned} \Rightarrow \textsf{e}^{ln \mathscr {A}_{\mathscr {S}_j}^{n}} \textsf{e}^{ln \mathscr {A}_{\mathscr {S}}^{n}}\le 1-\textsf{e}^{ln \left( 1-\mathscr {A}_{\mathscr {S}_j}^{n}\right) } \textsf{e}^{ln \left( 1-\mathscr {A}_{\mathscr {S}}^{n}\right) } \end{aligned}$$

$$\begin{aligned} \Rightarrow \frac{1}{\textsf{e}^{\left( -ln \mathscr {A}_{\mathscr {S}_j}^{n}\right) } \textsf{e}^{\left( -ln \mathscr {A}_{\mathscr {S}}^{n}\right) }}\le 1-\frac{1}{\textsf{e}^{\left( -ln \left( 1-\mathscr {A}_{\mathscr {S}_j}^{n}\right) \right) } \textsf{e}^{\left( -ln \left( 1-\mathscr {A}_{\mathscr {S}}^{n}\right) \right) }} \end{aligned}$$

$$\begin{aligned} \Rightarrow \frac{1}{\textsf{e}^{\left( \left( -ln \mathscr {A}_{\mathscr {S}_j}^{n}\right) ^\mathscr {I}\right) ^{\frac{1}{\mathscr {I}}}} \textsf{e}^{\left( \left( -ln \mathscr {A}_{\mathscr {S}}^{n}\right) ^\mathscr {I}\right) ^{\frac{1}{\mathscr {I}}}}}\le 1-\frac{1}{\textsf{e}^{\left( \left( -ln \left( 1-\mathscr {A}_{\mathscr {S}_j}^{n}\right) \right) ^\mathscr {I}\right) ^{\frac{1}{\mathscr {I}}}} \textsf{e}^{\left( \left( -ln \left( 1-\mathscr {A}_{\mathscr {S}}^{n}\right) \right) ^\mathscr {I}\right) ^{\frac{1}{\mathscr {I}}}}} \end{aligned}$$

$$\begin{aligned} \Rightarrow \frac{1}{\textsf{e}^{\left( \left( -ln \mathscr {A}_{\mathscr {S}_j}^{n}\right) ^\mathscr {I}+\left( -ln \mathscr {A}_{\mathscr {S}}^{n}\right) ^\mathscr {I}\right) ^{\frac{1}{\mathscr {I}}}}}\le 1-\frac{1}{\textsf{e}^{\left( \left( -ln \left( 1-\mathscr {A}_{\mathscr {S}_j}^{n}\right) \right) ^\mathscr {I}+\left( -ln \left( 1-\mathscr {A}_{\mathscr {S}}^{n}\right) \right) ^\mathscr {I}\right) ^{\frac{1}{\mathscr {I}}}}} \end{aligned}$$

$$\begin{aligned} \Rightarrow \left( \frac{1}{\textsf{e}^{\left( \left( -ln \mathscr {A}_{\mathscr {S}_j}^{n}\right) ^\mathscr {I}+\left( -ln \mathscr {A}_{\mathscr {S}}^{n}\right) ^\mathscr {I}\right) }}\right) ^{\frac{1}{\mathscr {I}}}\le 1-\left( \frac{1}{\textsf{e}^{\left( \left( -ln \left( 1-\mathscr {A}_{\mathscr {S}_j}^{n}\right) \right) ^\mathscr {I}+\left( -ln \left( 1-\mathscr {A}_{\mathscr {S}}^{n}\right) \right) ^\mathscr {I}\right) }}\right) ^{\frac{1}{\mathscr {I}}} \end{aligned}$$

$$\begin{aligned} \Rightarrow \textsf{e}^{-\left( \left( -ln \mathscr {A}_{\mathscr {S}_j}^{n}\right) ^\mathscr {I}+\left( -ln \mathscr {A}_{\mathscr {S}}^{n}\right) ^\mathscr {I}\right) ^{\frac{1}{\mathscr {I}}}}\le 1-\textsf{e}^{-\left( \left( -ln \left( 1-\mathscr {A}_{\mathscr {S}_j}^{n}\right) \right) ^\mathscr {I}+\left( -ln \left( 1-\mathscr {A}_{\mathscr {S}}^{n}\right) \right) ^\mathscr {I}\right) ^{\frac{1}{\mathscr {I}}}} \end{aligned}$$

and hence,

$$\begin{aligned} \root n \of {\textsf{e}^{-\left( \left( -ln \mathscr {A}_{\mathscr {S}_j}^{n}\right) ^\mathscr {I}+\left( -ln \mathscr {A}_{\mathscr {S}}^{n}\right) ^\mathscr {I}\right) ^{\frac{1}{\mathscr {I}}}}}\le \root n \of {1-\textsf{e}^{-\left( \left( -ln \left( 1-\mathscr {A}_{\mathscr {S}_j}^{n}\right) \right) ^\mathscr {I}+\left( -ln \left( 1-\mathscr {A}_{\mathscr {S}}^{n}\right) \right) ^\mathscr {I}\right) ^{\frac{1}{\mathscr {I}}}}}. \end{aligned}$$

Similarly we have

$$\begin{aligned} \root n \of {\textsf{e}^{-\left( \left( -ln \mathscr {D}_{\mathscr {A}_{\mathscr {S}_j}}^{n}\right) ^\mathscr {I}+\left( -ln \mathscr {D}_{\mathscr {A}_{\mathscr {S}}}^{n}\right) ^\mathscr {I}\right) ^{\frac{1}{\mathscr {I}}}}} \le \root n \of {1-\textsf{e}^{-\left( \left( -ln \left( 1-\mathscr {D}_{\mathscr {A}_{\mathscr {S}_j}}^{n}\right) \right) ^\mathscr {I}+\left( -ln \left( 1-\mathscr {D}_{\mathscr {A}_{\mathscr {S}}}^{n}\right) \right) ^\mathscr {I}\right) ^{\frac{1}{\mathscr {I}}}}} , \end{aligned}$$

$$\begin{aligned} \root m \of {1-\textsf{e}^{-\left( \left( -ln \left( 1-\mathscr {B}_{\mathscr {S}_j}^{m}\right) \right) ^\mathscr {I}+\left( -ln \left( 1-\mathscr {B}_{\mathscr {S}}^{m}\right) \right) ^\mathscr {I}\right) ^{\frac{1}{\mathscr {I}}}}} \ge \root m \of {\textsf{e}^{-\left( \left( -ln \mathscr {B}_{\mathscr {S}_j}^{m}\right) ^\mathscr {I}+\left( -ln \mathscr {B}_{\mathscr {S}}^{m}\right) ^\mathscr {I}\right) ^{\frac{1}{\mathscr {I}}}}}, \end{aligned}$$

and

$$\begin{aligned} \root m \of {1-\textsf{e}^{-\left( \left( -ln \left( 1-\mathscr {D}_{\mathscr {B}_{\mathscr {S}_j}}^{m}\right) \right) ^\mathscr {I}+\left( -ln \left( 1-\mathscr {D}_{\mathscr {B}_{\mathscr {S}}}^{m}\right) \right) ^\mathscr {I}\right) ^{\frac{1}{\mathscr {I}}}}}\ge \root m \of {\textsf{e}^{-\left( \left( -ln \mathscr {D}_{\mathscr {B}_{\mathscr {S}_j}}^{m}\right) ^\mathscr {I}+\left( -ln \mathscr {D}_{\mathscr {B}_{\mathscr {S}}}^{m}\right) ^\mathscr {I}\right) ^{\frac{1}{\mathscr {I}}}}}. \end{aligned}$$

Thus, $$\mathscr {S}_j\boxtimes \mathscr {S}=\left( \begin{array}{c} \root n \of {\textsf{e}^{-\left( \left( -ln \mathscr {A}_{\mathscr {S}_j}^{n}\right) ^\mathscr {I}+\left( -ln \mathscr {A}_{\mathscr {S}}^{n}\right) ^\mathscr {I}\right) ^{\frac{1}{\mathscr {I}}}}}\\ \textsf{e}^{2 i \pi \root n \of {\textsf{e}^{-\left( \left( -ln \mathscr {D}_{\mathscr {A}_{\mathscr {S}_j}}^{n}\right) ^\mathscr {I}+\left( -ln \mathscr {D}_{\mathscr {A}_{\mathscr {S}}}^{n}\right) ^\mathscr {I}\right) ^{\frac{1}{\mathscr {I}}}}}},\\ \root m \of {1-\textsf{e}^{-\left( \left( -ln \left( 1-\mathscr {B}_{\mathscr {S}_j}^{m}\right) \right) ^\mathscr {I}+\left( -ln \left( 1-\mathscr {B}_{\mathscr {S}}^{m}\right) \right) ^\mathscr {I}\right) ^{\frac{1}{\mathscr {I}}}}}\\ \textsf{e}^{2 i \pi \root m \of {1-\textsf{e}^{-\left( \left( -ln \left( 1-\mathscr {D}_{\mathscr {B}_{\mathscr {S}_j}}^{m}\right) \right) ^\mathscr {I}+\left( -ln \left( 1-\mathscr {D}_{\mathscr {B}_{\mathscr {S}}}^{m}\right) \right) ^\mathscr {I}\right) ^{\frac{1}{\mathscr {I}}}}}} \end{array}\right) \subseteq \mathscr {S}_{j} \boxplus \mathscr {S}=$$

$$\left( \begin{array}{c} \root n \of {1-\textsf{e}^{-\left( \left( -ln \left( 1-\mathscr {A}_{\mathscr {S}_j}^{n}\right) \right) ^\mathscr {I}+\left( -ln \left( 1-\mathscr {A}_{\mathscr {S}}^{n}\right) \right) ^\mathscr {I}\right) ^{\frac{1}{\mathscr {I}}}}}\\ \textsf{e}^{2 i \pi \root n \of {1-\textsf{e}^{-\left( \left( -ln \left( 1-\mathscr {D}_{\mathscr {A}_{\mathscr {S}_j}}^{n}\right) \right) ^\mathscr {I}+\left( -ln \left( 1-\mathscr {D}_{\mathscr {A}_{\mathscr {S}}}^{n}\right) \right) ^\mathscr {I}\right) ^{\frac{1}{\mathscr {I}}}}}},\\ \root m \of {\textsf{e}^{-\left( \left( -ln \mathscr {B}_{\mathscr {S}_j}^{m}\right) ^\mathscr {I}+\left( -ln \mathscr {B}_{\mathscr {S}}^{m}\right) ^\mathscr {I}\right) ^{\frac{1}{\mathscr {I}}}}}\\ \textsf{e}^{2 i \pi \root m \of {\textsf{e}^{-\left( \left( -ln \mathscr {D}_{\mathscr {B}_{\mathscr {S}_j}}^{m}\right) ^\mathscr {I}+\left( -ln \mathscr {D}_{\mathscr {B}_{\mathscr {S}}}^{m}\right) ^\mathscr {I}\right) ^{\frac{1}{\mathscr {I}}}}}} \end{array}\right)$$.

Therefore, Theorem 4.5 simplifies the process of proving all the parts. $\square$

### Theorem 4.9

Let $\left\{ \mathscr {S}_{j}=\left( \mathscr {A}_{\mathscr {S}_j} \cdot e^{i.2 \pi \mathscr {D}_{\mathscr {A}_{\mathscr {S}_j}}}, \mathscr {B}_{\mathscr {S}_j} \cdot e^{i \cdot 2 \pi \mathscr {D}_{\mathscr {B}_{\mathscr {S}_j}}}\right) \right\} _{j=1, \ldots , \rho }$ and $\left\{ \widetilde{\mathscr {S}}_{j}=\left( \mathscr {A}_{\widetilde{\mathscr {S}}_{j}} \cdot e^{i.2 \pi \mathscr {D}_{\mathscr {A}_{\widetilde{\mathscr {S}}_{j}}}}, \mathscr {B}_{\widetilde{\mathscr {S}}_{j}} \cdot e^{i \cdot 2 \pi \mathscr {D}_{\mathscr {B}_{\widetilde{\mathscr {S}}_{j}}}}\right) \right\} _{j=1, \ldots , \rho }$ be two collections of $\rho$ Cn,m-ROFNs. Suppose the weight vector $\mathscr {K}=$ $\left( \mathscr {K}_{1}, \ldots , \mathscr {K}_{\rho }\right)$ satisfies $\sum _{j=1}^{\rho } \mathscr {K}_{j}=1$, it follows that:

1. Cn,m-ROFAAWA$$\left( \mathscr {S}_{1} \boxplus \widetilde{\mathscr {S}}_{1}, \ldots , \mathscr {S}_{\rho } \boxplus \widetilde{\mathscr {S}}_{\rho }\right) \ge \text{Cn,m-ROFAAWA}\left( \mathscr {S}_{1} \boxtimes \widetilde{\mathscr {S}}_{1}, \ldots , \mathscr {S}_{\rho } \boxtimes \widetilde{\mathscr {S}}_{\rho }\right)$$
2. Cn,m-ROFAAWG$$\left( \mathscr {S}_{1} \boxplus \widetilde{\mathscr {S}}_{1}, \ldots , \mathscr {S}_{\rho } \boxplus \widetilde{\mathscr {S}}_{\rho }\right) \ge \operatorname {Cn,m-ROFAAWG}\left( \mathscr {S}_{1} \boxtimes \widetilde{\mathscr {S}}_{1}, \ldots , \mathscr {S}_{\rho } \boxtimes \widetilde{\mathscr {S}}_{\rho }\right)$$

### Proof

Given that for any Cn,m-ROFNs $\mathscr {S}_{j}$ and $\widetilde{\mathscr {S}}_{j}$, we have the inclusion

$$\begin{aligned} \mathscr {S}_{j} \boxtimes \widetilde{\mathscr {S}}_{j}\subseteq \mathscr {S}_{j} \boxplus \widetilde{\mathscr {S}}_{j}. \end{aligned}$$

Thus, the proofs of all parts follow easily from Theorem 4.5. $\square$

## Assessing the effectiveness of our methodologies for MADM

This section proposes a multiple-attribute decision-making approach tailored to the Cn,m-ROFS environment. Real-world examples are included to demonstrate the applicability and effectiveness of the method, emphasizing its practical utility in decision-making scenarios.

In many MADM problems, decision makers are faced with two finite collections: a set of *z* alternatives $\{\mathscr{A}\mathscr{L}_1, \mathscr{A}\mathscr{L}_2, \ldots , \mathscr{A}\mathscr{L}_z\}$ and a set of $\rho$ attributes $\{\mathscr{A}\mathscr{T}_1, \mathscr{A}\mathscr{T}_2, \ldots , \mathscr{A}\mathscr{T}_\rho \}$. To address such problems, each alternative is evaluated against the given attributes, and a weight vector $\mathscr {K} = (\mathscr {K}_1, \mathscr {K}_2, \ldots , \mathscr {K}_\rho )^{T}$ is assigned to the attributes, where $\mathscr {K}_i > 0$ and $\sum _{i=1}^\rho \mathscr {K}_i = 1$. This weight vector reflects the relative influence of the attributes in the overall decision-making procedure. The evaluations are organized into a decision matrix $\mathscr{D}\mathscr{M}$, which is defined as:

$$\mathscr{D}\mathscr{M} = \big [\mathscr {S}_{ju}\big ] = \big [\left( \mathscr {A}_{\mathscr {S}_{ju}} \cdot e^{i .2 \pi \mathscr {D}_{\mathscr {A}_{\mathscr {S}_{ju}}}}, \mathscr {B}_{\mathscr {S}_{ju}} \cdot e^{i \cdot 2 \pi \mathscr {D}_{\mathscr {B}_{\mathscr {S}_{ju}}}}\right) \big ]_{z\times \rho }$$

9 $$\begin{aligned} =\left[ \begin{array}{cccc} \mathscr {S}_{11} & \mathscr {S}_{12} & \cdots & \mathscr {S}_{1 \rho } \\ \mathscr {S}_{21} & \mathscr {S}_{22} & \cdots & \mathscr {S}_{2 \rho } \\ \vdots & \vdots & & \ddots \\ \mathscr {S}_{z 1} & \mathscr {S}_{z 2} & \cdots & \mathscr {S}_{z \rho } \end{array}\right] , \end{aligned}$$

where each element $\mathscr {S}_{ju}$ represents the evaluation of alternative $\mathscr{A}\mathscr{L}_j$ ($j=1,2,\ldots ,z$) with respect to attribute $\mathscr{A}\mathscr{T}_u$ ($u=1,2,\ldots ,\rho$). Specifically, $\mathscr {A}_{\mathscr {S}_{ju}} \cdot e^{i.2 \pi \mathscr {D}_{\mathscr {A}_{\mathscr {S}_{ju}}}}$ and $\mathscr {B}_{\mathscr {S}_{ju}} \cdot e^{i \cdot 2 \pi \mathscr {D}_{\mathscr {B}_{\mathscr {S}_{ju}}}}$ are the membership and non-membership grades, respectively, assigned by the decision maker. Here, $\mathscr {A}_{\mathscr {S}_{ju}}$ and $\mathscr {B}_{\mathscr {S}_{ju}}$ represent the amplitude terms of the membership and non-membership grades, while $\mathscr {D}_{\mathscr {A}_{\mathscr {S}_{ju}}}$ and $\mathscr {D}_{\mathscr {B}_{\mathscr {S}_{ju}}}$ are their corresponding phase terms. To effectively analyze this decision matrix and select the most suitable alternatives, we propose the use of Cn,m-ROFAAWA and Cn,m-ROFAAWG operators derived from Aczél-Alsina formulations. The provided evaluation matrix is assessed systematically through a structured set of algorithmic steps, which ensures an accurate and reliable decision-making process.

### Algorithm: decision-making using Cn,m-ROFNs

- **Step 1:** Problem definition and criteria identification: Clearly define the decision-making problem and identify the criteria for evaluating alternatives. Ensure all relevant attributes and alternatives are properly outlined.
- **Step 2:** Formulate the complex decision matrix: Generate the Cn,m-ROF decision matrix $\mathscr{D}\mathscr{M} = \big [\mathscr {S}_{ju}\big ]_{z\times \rho }$ using Cn,m-ROFNs. Each element of the matrix represents the membership and non-membership grades of alternatives in relation to the attributes.
- **Step 3:** Standardize the decision matrix: Normalize the complex decision matrix $\mathscr{D}\mathscr{M}$ to ensure consistency and comparability. The normalization step adjusts the values in the decision matrix to a uniform scale.
- **Step 4:** Apply evaluation operators: Evaluate the alternatives using the Cn,m-ROFAAWA and Cn,m-ROFAAWG methods. Compute the aggregated values for each alternative as follows:

- Compute $$\operatorname {Cn,m-AAWA_{j}} = \text {Cn,m-ROFAAWA}\left( \mathscr {S}_{j1}, \mathscr {S}_{j2}, \ldots , \mathscr {S}_{j\rho }\right)$$:
  10 $$\begin{aligned} \operatorname {Cn,m-AAWA_{j}} = \left( \begin{array}{c} \root n \of {1-\textsf{e}^{-\left( \sum _{j=1}^{\rho } \mathscr {K}_{j}\left( -ln \left( 1-\mathscr {A}_{\mathscr {S}_j}^{n}\right) \right) ^\mathscr {I}\right) ^{\frac{1}{\mathscr {I}}}}}\\ \textsf{e}^{2 i \pi \root n \of {1-\textsf{e}^{-\left( \sum _{j=1}^{\rho } \mathscr {K}_{j}\left( -ln \left( 1-\mathscr {D}_{\mathscr {A}_{\mathscr {S}_j}}^{n}\right) \right) ^\mathscr {I}\right) ^{\frac{1}{\mathscr {I}}}}}},\\ \root m \of {\textsf{e}^{-\left( \sum _{j=1}^{\rho } \mathscr {K}_{j}\left( -ln \mathscr {B}_{\mathscr {S}_j}^{m}\right) ^\mathscr {I}\right) ^{\frac{1}{\mathscr {I}}}}}\\ \textsf{e}^{2 i \pi \root m \of {\textsf{e}^{-\left( \sum _{j=1}^{\rho } \mathscr {K}_{j}\left( -ln \mathscr {D}_{\mathscr {B}_{\mathscr {S}_j}}^{m}\right) ^\mathscr {I}\right) ^{\frac{1}{\mathscr {I}}}}}} \end{array}\right) \end{aligned}$$
- Compute $$\operatorname {Cn,m-AAWG_{j}} = \text {Cn,m-ROFAAWG}\left( \mathscr {S}_{j1}, \mathscr {S}_{j2}, \ldots , \mathscr {S}_{j\rho }\right)$$:
  11 $$\begin{aligned} \operatorname {Cn,m-AAWG_{j}} = \left( \begin{array}{c} \root n \of {\textsf{e}^{-\left( \sum _{j=1}^{\rho } \mathscr {K}_{j}\left( -ln \mathscr {A}_{\mathscr {S}_j}^{n}\right) ^\mathscr {I}\right) ^{\frac{1}{\mathscr {I}}}}}\\ \textsf{e}^{2 i \pi \root n \of {\textsf{e}^{-\left( \sum _{j=1}^{\rho } \mathscr {K}_{j}\left( -ln \mathscr {D}_{\mathscr {A}_{\mathscr {S}_j}}^{n}\right) ^\mathscr {I}\right) ^{\frac{1}{\mathscr {I}}}}}},\\ \root m \of {1-\textsf{e}^{-\left( \sum _{j=1}^{\rho } \mathscr {K}_{j}\left( -ln \left( 1-\mathscr {B}_{\mathscr {S}_j}^{m}\right) \right) ^\mathscr {I}\right) ^{\frac{1}{\mathscr {I}}}}}\\ \textsf{e}^{2 i \pi \root m \of {1-\textsf{e}^{-\left( \sum _{j=1}^{\rho } \mathscr {K}_{j}\left( -ln \left( 1-\mathscr {D}_{\mathscr {B}_{\mathscr {S}_j}}^{m}\right) \right) ^\mathscr {I}\right) ^{\frac{1}{\mathscr {I}}}}}} \end{array}\right) \end{aligned}$$
- **Step 5:** Score computation: For every alternative $j=1,2,\ldots ,z$, calculate the final scores for $\operatorname {Cn,m-AAWA_{j}}$ and $\operatorname {Cn,m-AAWG_{j}}$ obtained from the operators in Step 4.
- **Step 6:** Rank the alternatives: According to the scores obtained in Step 5, rank the alternatives in descending order of preference. This ranking provides the optimal order of alternatives, identifying the most favorable choice.

### MADM problem: comprehensive evaluation for selecting the best renewable energy source

Renewable energy (RE) is a cornerstone of sustainable development, reducing dependency on fossil fuels and mitigating environmental degradation. As part of its commitment to environmental sustainability, a government agency aims to invest in a renewable energy project. However, selecting the most suitable renewable energy source involves a complex MADM process. This selection must account for multiple criteria, including cost, efficiency, environmental impact, reliability, and social acceptance. To facilitate this decision, we apply the proposed algorithm to identify the optimal alternative among various renewable energy sources.

### Alternatives ($z$):

The decision-making process involves evaluating four renewable energy sources (${RE}_1, {RE}_2, {RE}_3, {RE}_4$), each representing a viable investment option:

- **Solar Energy** (${RE}_1$): Harnesses sunlight using photovoltaic cells or solar thermal systems.
- **Wind Energy** (${RE}_2$): Converts kinetic energy from wind into electricity using turbines.
- **Hydropower** (${RE}_3$): Generates electricity from the movement of water, typically using dams.
- **Biomass Energy** (${RE}_4$): Utilizes organic materials such as wood, agricultural residues, or waste for energy production.

### Attributes ($\rho$):

The following critical attributes, labeled ($\mathscr{A}\mathscr{T}_1, \mathscr{A}\mathscr{T}_2, \mathscr{A}\mathscr{T}_3, \mathscr{A}\mathscr{T}_4, \mathscr{A}\mathscr{T}_5$), are used to assess every alternative:

- **Cost **($\mathscr{A}\mathscr{T}_1$) : Includes the initial investment, operational, and maintenance costs. This attribute directly influences the financial feasibility of the project.
- **Energy efficiency** ($\mathscr{A}\mathscr{T}_2$) : Measures how effectively the alternative converts resources into usable energy, reflecting the system’s technical performance.
- **Environmental impact **($\mathscr{A}\mathscr{T}_3$) : Evaluates the ecological footprint, including greenhouse gas emissions, resource depletion, and habitat disruption.
- **Reliability **($\mathscr{A}\mathscr{T}_4$) : Considers the consistency of energy output under varying conditions and the technology’s adaptability to different environments.
- **Social acceptance **($\mathscr{A}\mathscr{T}_5$): Assesses public support, regulatory alignment, and societal benefits of the energy source.

### Decision matrix construction ($\mathscr{D}\mathscr{M}$):

The decision matrix is constructed using Cn,m-ROFNs. Each element in the matrix represents the evaluation of an alternative ${RE}_j$ ($j = 1, 2, \dots , z$) against an attribute $\mathscr{A}\mathscr{T}_u$ ($u = 1, 2, \dots , \rho$). The matrix is denoted as:

$$\mathscr{D}\mathscr{M} = \big [\mathscr {S}_{ju}\big ]_{z \times \rho },$$

where

- $$\mathscr {S}_{ju} = (\mathscr {A}_{\mathscr {S}_{ju}} \cdot e^{i \cdot 2 \pi \mathscr {D}_{\mathscr {A}_{\mathscr {S}_{ju}}}}, \mathscr {B}_{\mathscr {S}_{ju}} \cdot e^{i \cdot 2 \pi \mathscr {D}_{\mathscr {B}_{\mathscr {S}_{ju}}}})$$
- $\mathscr {A}_{\mathscr {S}_{ju}}$ reflects the extent to which an alternative satisfies a given criterion.
- $\mathscr {B}_{\mathscr {S}_{ju}}$ represents the degree of non-satisfaction.
- phase terms ($\mathscr {D}_{\mathscr {A}_{\mathscr {S}_{ju}}}, \mathscr {D}_{\mathscr {B}_{\mathscr {S}_{ju}}}$) are included to capture additional uncertainty or contextual preferences.

### Attribute weight vector ($\mathscr {K}$):

The following weight vector expresses the relative contribution of every attribute:

$$\mathscr {K} = (\mathscr {K}_1, \mathscr {K}_2, \dots , \mathscr {K}_\rho ),$$

where $\mathscr {K}_u > 0$ and $\sum _{u=1}^{\rho } \mathscr {K}_u = 1$. For this problem, the weights might be distributed as follows:

$$\mathscr {K} = (0.24, 0.20, 0.22, 0.21, 0.13).$$

Here, cost is assigned the highest priority ($0.24$), while other criteria are given equal or slightly lower importance based on stakeholder preferences.

### Goal and methodology

The primary goal is to rank the alternatives based on their suitability across all attributes and select the optimal renewable energy source. This involves:

- Constructing and normalizing the decision matrix as shown in Table 1 ($Step~2$ and $Step~3$ in Algorithm).
- Applying the proposed $\operatorname {Cn,m-AAWA_{j} =Cn,m-ROFAAWA}(\mathscr {S}_{j1}, \mathscr {S}_{j2},..., \mathscr {S}_{j5})$, and $\operatorname {Cn,m-AAWG_{j} =Cn,m-ROFAAWG}(\mathscr {S}_{j1}, \mathscr {S}_{j2},..., \mathscr {S}_{j5})$ operators to compute aggregated values for each alternative along with the parameters $n=3$, $m=4$ and $\mathscr {I} = 4, 5$ as shown in Table 2 ($Step~4$).
- Calculating final scores for the alternatives based on the operators’ results as shown in Table 3 ($Step~5$).
- Ranking the alternatives in descending order of scores to identify the most favorable option by utilizing Definition 2.4, as presented in Table 4 ($Step~6$).

### Outcome

The rankings of the options generated by the $\operatorname {C3,4-ROFAAWA}$ operator with the parameter $\mathscr {I}=4, 5$ are as follows:

$$\begin{aligned} {RE}_{2} \succ {RE}_{3} \succ {RE}_{4} \succ {RE}_{1}. \end{aligned}$$

Similarly, the rankings derived from the $\operatorname {C3,4-ROFWG}$ operator for the same parameter $\mathscr {I}=4, 5$ are given as:

$$\begin{aligned} {RE}_{2} \succ {RE}_{1} \succ {RE}_{3} \succ {RE}_{4}. \end{aligned}$$

**Fig. 2:**
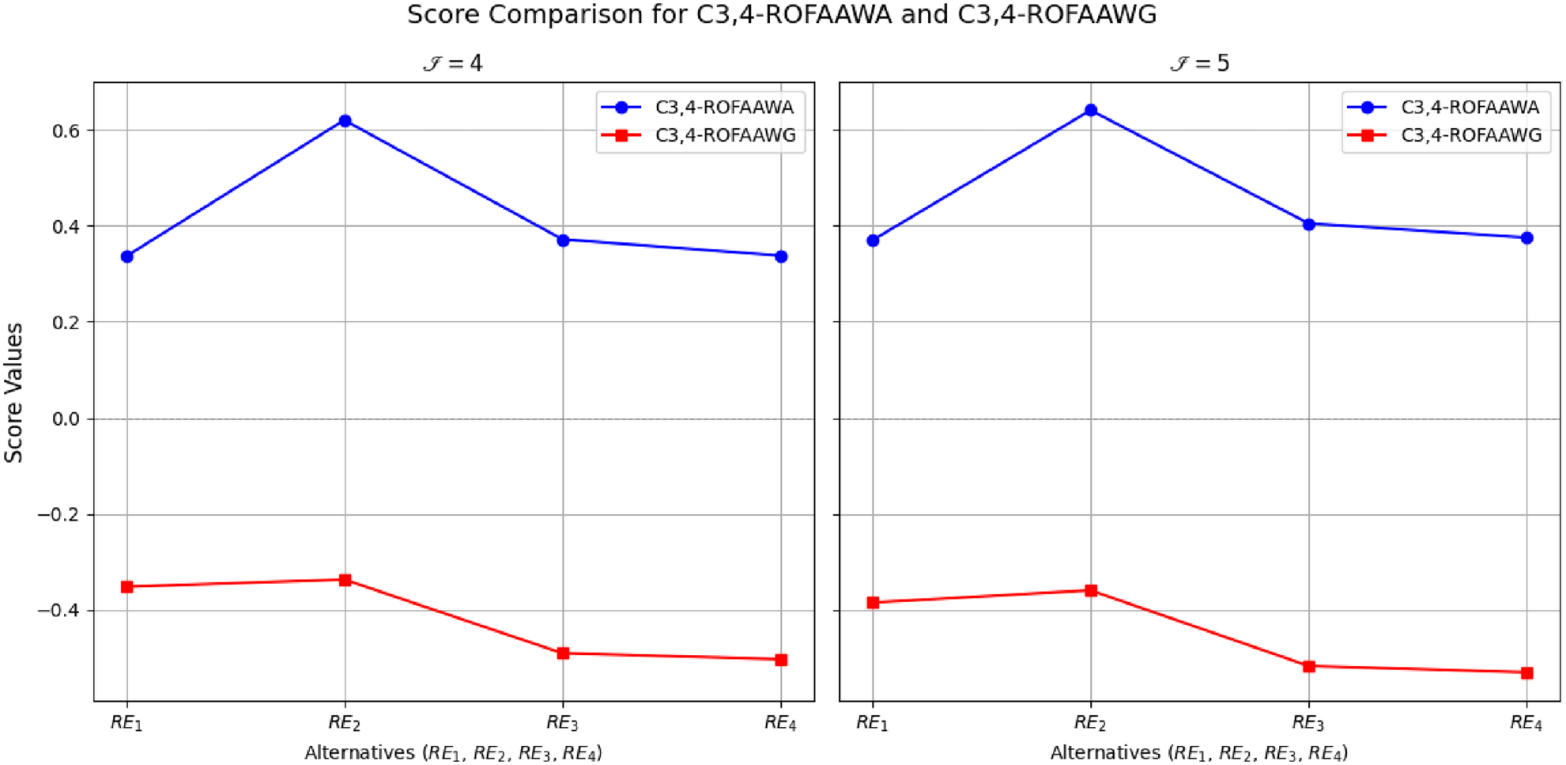
Score values of C3,4-ROFAAWA and C3,4-ROFAAWG operators.

From these results, it is evident that ${RE}_2$ emerges as the top-ranked option across both operators. Additionally, the outcomes generated by the $\operatorname {C3,4-ROFAAWA}$ and $\operatorname {C3,4-ROFAAWG}$ operators are visually represented in Fig. 2.

**Table 1:** C3,4-ROF values.

RE	$\mathscr{A}\mathscr{T}_1$	$\mathscr{A}\mathscr{T}_2$	$\mathscr{A}\mathscr{T}_3$	$\mathscr{A}\mathscr{T}_4$	$\mathscr{A}\mathscr{T}_5$
${RE}_1$	$(0.8e^{i2\pi (0.5)}, 0.8e^{i2\pi (0.4)})$	$(0.9e^{i2\pi (0.3)}, 0.7e^{i2\pi (0.5)})$	$(0.6e^{i2\pi (0.7)}, 0.9e^{i2\pi (0.8)})$	$(0.6e^{i2\pi (0.8)}, 0.9e^{i2\pi (0.5)})$	$(0.8e^{i2\pi (0.4)}, 0.6e^{i2\pi (0.9)})$
${RE}_2$	$(0.2e^{i2\pi (0.6)}, 0.9e^{i2\pi (0.8)})$	$(0.9e^{i2\pi (0.5)}, 0.1e^{i2\pi (0.7)})$	$(0.8e^{i2\pi (0.9)}, 0.8e^{i2\pi (0.2)})$	$(0.1e^{i2\pi (0.9)}, 0.9e^{i2\pi (0.1)})$	$(0.5e^{i2\pi (0.7)}, 0.6e^{i2\pi (0.7)})$
${RE}_3$	$(0.3e^{i2\pi (0.6)}, 0.8e^{i2\pi (0.9)})$	$(0.4e^{i2\pi (0.5)}, 0.9e^{i2\pi (0.4)})$	$(0.9e^{i2\pi (0.1)}, 0.6e^{i2\pi (0.2)})$	$(0.8e^{i2\pi (0.8)}, 0.8e^{i2\pi (0.8)})$	$(0.5e^{i2\pi (0.4)}, 0.7e^{i2\pi (0.8)})$
${RE}_4$	$(0.2e^{i2\pi (0.5)}, 0.7e^{i2\pi (0.8)})$	$(0.8e^{i2\pi (0.9)}, 0.7e^{i2\pi (0.7)})$	$(0.6e^{i2\pi (0.1)}, 0.6e^{i2\pi (0.4)})$	$(0.3e^{i2\pi (0.2)}, 0.9e^{i2\pi (0.9)})$	$(0.6e^{i2\pi (0.7)}, 0.8e^{i2\pi (0.6)})$

**Table 2:** Aggregated C3,4-ROF information matrix.

$\mathscr {I}$	RE	$\operatorname {C3,4-ROFAAWA}$	$\operatorname {C3,4-ROFAAWG}$
	${RE}_1$	$(0.8419 e^{i2\pi (0.7328)}, 0.7147 e^{i2\pi (0.4882)})$	$(0.6591 e^{i2\pi (0.4192)}, 0.8734 e^{i2\pi (0.8329)})$
	${RE}_2$	$(0.8392 e^{i2\pi (0.8676)}, 0.2143 e^{i2\pi (0.1923)})$	$(0.1907 e^{i2\pi (0.6039)}, 0.8747 e^{i2\pi (0.7490)})$
4	${RE}_3$	$(0.8428 e^{i2\pi (0.7281)}, 0.6925 e^{i2\pi (0.3237)})$	$(0.4039 e^{i2\pi (0.2046)}, 0.8498 e^{i2\pi (0.8546)})$
	${RE}_4$	$(0.7261 e^{i2\pi (0.8354)}, 0.6790 e^{i2\pi (0.5273)})$	$(0.3011 e^{i2\pi (0.1895)}, 0.8486 e^{i2\pi (0.8494)})$
	${RE}_1$	$(0.8520 e^{i2\pi (0.7446)}, 0.6996 e^{i2\pi (0.4775)})$	$(0.6488 e^{i2\pi (0.4019)}, 0.8784 e^{i2\pi (0.8457)})$
	${RE}_2$	$(0.8508 e^{i2\pi (0.8742)}, 0.1884 e^{i2\pi (0.1754)})$	$(0.1746 e^{i2\pi (0.5897)}, 0.8795 e^{i2\pi (0.7578)})$
5	${RE}_3$	$(0.8537 e^{i2\pi (0.7422)}, 0.6793 e^{i2\pi (0.3007)})$	$(0.3883 e^{i2\pi (0.1820)}, 0.8585 e^{i2\pi (0.8630)})$
	${RE}_4$	$(0.7404 e^{i2\pi (0.8490)}, 0.6702 e^{i2\pi (0.5054)})$	$(0.2846 e^{i2\pi (0.1733)}, 0.8587 e^{i2\pi (0.8590)})$

**Table 3:** Final score values C3,4-ROFAAWA and C3,4-ROFAAWG operators.

Parameter $\mathscr {I}$	RE	$\dot{s}(\operatorname {C3,4-ROFAAWA})$	$\dot{s}(\operatorname {C3,4-ROFAAWG})$
	${RE}_1$	0.3363	-0.3516
	${RE}_2$	0.6203	-0.3364
4	${RE}_3$	0.3718	-0.4902
	${RE}_4$	0.3379	-0.5025
	${RE}_1$	0.3698	-0.3845
	${RE}_2$	0.6409	-0.3590
5	${RE}_3$	0.4050	-0.5167
	${RE}_4$	0.3755	-0.5300

**Table 4:** Rankings of the outcomes for our application.

Parameter $\mathscr {I}$	Operators	Ranking	Best Renewable Energy
4	$\operatorname {C3,4-ROFAAWA}$	${RE}_{2} \succ {RE}_{3} \succ {RE}_{4} \succ {RE}_{1}$	${RE}_{2}$
	$\operatorname {C3,4-ROFAAWG}$	${RE}_{2} \succ {RE}_{1} \succ {RE}_{3} \succ {RE}_{4}$	${RE}_{2}$
	$\operatorname {C3,4-ROFAAWA}$	${RE}_{2} \succ {RE}_{3} \succ {RE}_{4} \succ {RE}_{1}$	${RE}_{2}$
5			
	$\operatorname {C3,4-ROFAAWG}$	${RE}_{2} \succ {RE}_{1} \succ {RE}_{3} \succ {RE}_{4}$	${RE}_{2}$

### Impact analysis

To analyze the behavior of the proposed methods, we introduce a set of parameters denoted by $\mathscr {I}$, which are utilized to describe the alternatives and assess the effect of varying parameter magnitudes. The outcomes of the selection process, derived from the Cn,m-ROFAAWA and Cn,m-ROFAAWG operators, are summarized in Tables 5 and 6. The results clearly indicate that increasing the parameter $\mathscr {I}$ leads to higher score values for the alternatives; however, the optimal choice remains unchanged. This behavior implies that the suggested methods exhibit isotonicity, allowing decision-makers to select the appropriate parameter value in accordance with their individual preferences. Additionally, the rankings of alternatives remain stable despite changes in $\mathscr {I}$, confirming the robustness and consistency of the proposed operators.

**Fig. 3:**
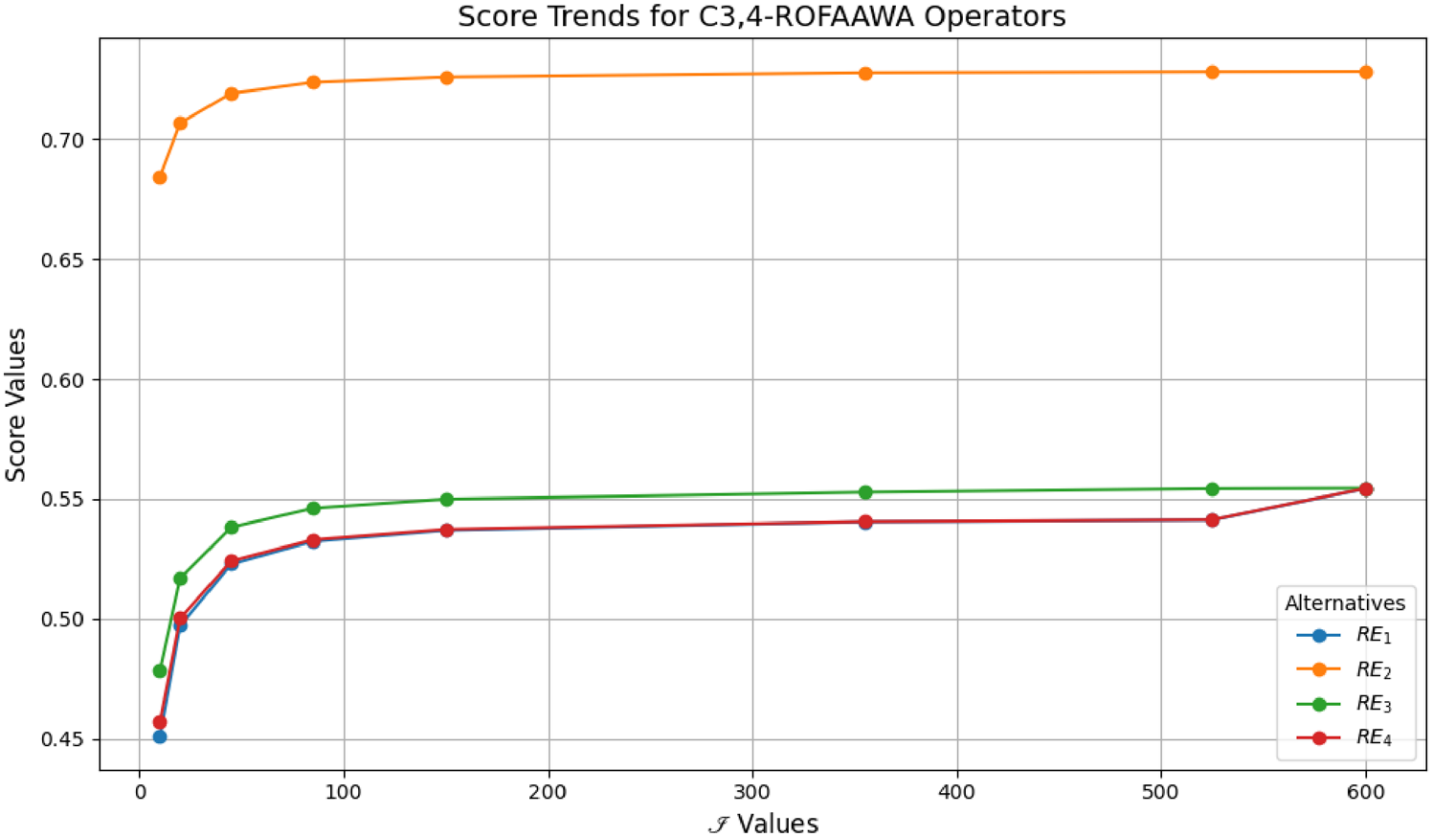
The outcomes of C3,4-ROFAAWA methods for $\mathscr {I}$.

**Fig. 4:**
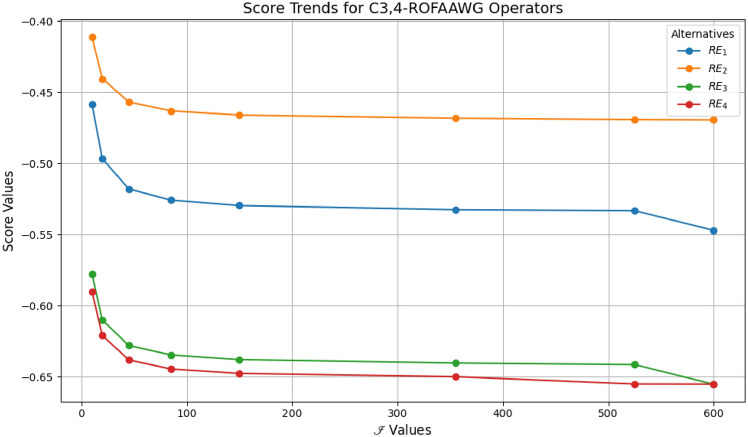
The outcomes of C3,4-ROFAAWG methods for $\mathscr {I}$.

The graphical effects of the Cn,m-ROFAAWA and Cn,m-ROFAAWG operators are also depicted in Figs. 3 and 4, respectively, to visually convey their operational behavior.

**Table 5:** The results ranking for the C3,4-ROFAAWA operators.

$\mathscr {I}$	$\dot{s}({RE}_1)$	$\dot{s}({RE}_2)$	$\dot{s}({RE}_3)$	$\dot{s}({RE}_4)$	Ranking
10	0.4510	0.6845	0.4785	0.4571	${RE}_{2} \succ {RE}_{3} \succ {RE}_{4} \succ {RE}_{1}$
20	0.4972	0.7069	0.5170	0.5003	${RE}_{2} \succ {RE}_{3} \succ {RE}_{4} \succ {RE}_{1}$
45	0.5228	0.7192	0.5382	0.5241	${RE}_{2} \succ {RE}_{3} \succ {RE}_{4} \succ {RE}_{1}$
85	0.5323	0.7238	0.5461	0.5330	${RE}_{2} \succ {RE}_{3} \succ {RE}_{4} \succ {RE}_{1}$
150	0.5369	0.7260	0.5499	0.5373	${RE}_{2} \succ {RE}_{3} \succ {RE}_{4} \succ {RE}_{1}$
355	0.5404	0.7278	0.5528	0.5405	${RE}_{2} \succ {RE}_{3} \succ {RE}_{4} \succ {RE}_{1}$
525	0.5412	0.7282	0.5543	0.5413	${RE}_{2} \succ {RE}_{3} \succ {RE}_{4} \succ {RE}_{1}$
600	0.5543	0.7283	0.5545	0.5544	${RE}_{2} \succ {RE}_{3} \succ {RE}_{4} \succ {RE}_{1}$

**Table 6:** The results ranking for the C3,4-ROFAAWG operators.

$\mathscr {I}$	$\dot{s}({RE}_1)$	$\dot{s}({RE}_2)$	$\dot{s}({RE}_3)$	$\dot{s}({RE}_4)$	Ranking
10	-0.4582	– 0.4111	– 0.5776	– 0.5900	${RE}_{2} \succ {RE}_{1} \succ {RE}_{3} \succ {RE}_{4}$
20	– 0.4967	– 0.4404	– 0.6101	– 0.6210	${RE}_{2} \succ {RE}_{1} \succ {RE}_{3} \succ {RE}_{4}$
45	– 0.5179	– 0.4568	– 0.6280	– 0.6381	${RE}_{2} \succ {RE}_{1} \succ {RE}_{3} \succ {RE}_{4}$
85	– 0.5258	– 0.4630	– 0.6346	– 0.6445	${RE}_{2} \succ {RE}_{1} \succ {RE}_{3} \succ {RE}_{4}$
150	– 0.5296	– 0.4660	– 0.6379	– 0.6476	${RE}_{2} \succ {RE}_{1} \succ {RE}_{3} \succ {RE}_{4}$
355	– 0.5325	– 0.4682	– 0.6403	– 0.6499	${RE}_{2} \succ {RE}_{1} \succ {RE}_{3} \succ {RE}_{4}$
525	– 0.5332	– 0.4692	– 0.6414	– 0.6550	${RE}_{2} \succ {RE}_{1} \succ {RE}_{3} \succ {RE}_{4}$
600	– 0.5470	– 0.4694	– 0.6552	– 0.6551	${RE}_{2} \succ {RE}_{1} \succ {RE}_{4} \succ {RE}_{3}$

## Comparative analysis of methodologies for decision-making

This section presents a detailed comparative analysis of the proposed aggregation operators against well-established methods, using the parameter $\mathscr {I}=7$ for consistent evaluation. The aim is to demonstrate the advantages of our methodology in terms of reliability, differentiation capability, and practical applicability in multiple attribute decision-making.

The aggregation operators considered in the comparison include:

- Cn,m-ROF weighted average (Cn,m-ROFWA) and geometric (Cn,m-ROFWG) operators proposed by Ibrahim^24^, evaluated for $n=2, m=5$ and $n=4, m=3$
- Cq-ROF weighted average (Cq-ROFWA) and geometric (Cq-ROFWG) operators from Liu et al.^22^, considered for $q=2,3,4,5$
- Cq-ROF aczel-alsina weighted average (Cq-ROFAAWA) and geometric (Cq-ROFAAWG) operators presented by Ali and Naeem^35^, for $q=2,3,4,5$
- CFF-based weighted average (CFFWA) and geometric (CFFWG) operators introduced by Chinnadurai et al.^23^, along with their aczel-alsina versions (CFFAAWA and CFFAAWG) by Chen et al.^34^.
- CPyF-based weighted average (CPyFWA) and geometric (CPyFWG) operators proposed by Ullah et al.^21^, together with their aczel-alsina variants (CPyFAAWA and CPyFAAWG) by Jin et al.^33^.
- CIF-based weighted average (CIFWA) and geometric (CIFWG) operators developed by Alkouri and Salleh^20^, including their aczel-alsina forms (CIFAAWA and CIFAAWG) from Mahmood et al.^32^.

Table 7 summarizes the numerical outcomes of these operators. Our proposed Cn,m-ROFAAWA and Cn,m-ROFAAWG operators demonstrate several key advantages:

1. **Consistent identification of the top-ranked alternative:** The proposed operators reliably identify the most suitable option (Wind Energy) across all parameter settings, confirming robustness.
2. **Enhanced differentiation among alternatives:** The operators provide higher discrimination between competing options compared to existing methods, enabling more precise decision-making.
3. **Operational success where other methods fail:** Several existing operators, especially under specific parameter settings, are unable to generate valid rankings, whereas the proposed operators maintain successful operation.
4. **Parameter stability:** Rankings remain stable under variations of the parameter $\mathscr {I}$, confirming isotonicity and applicability in practical decision-making contexts.

**Fig. 5:**
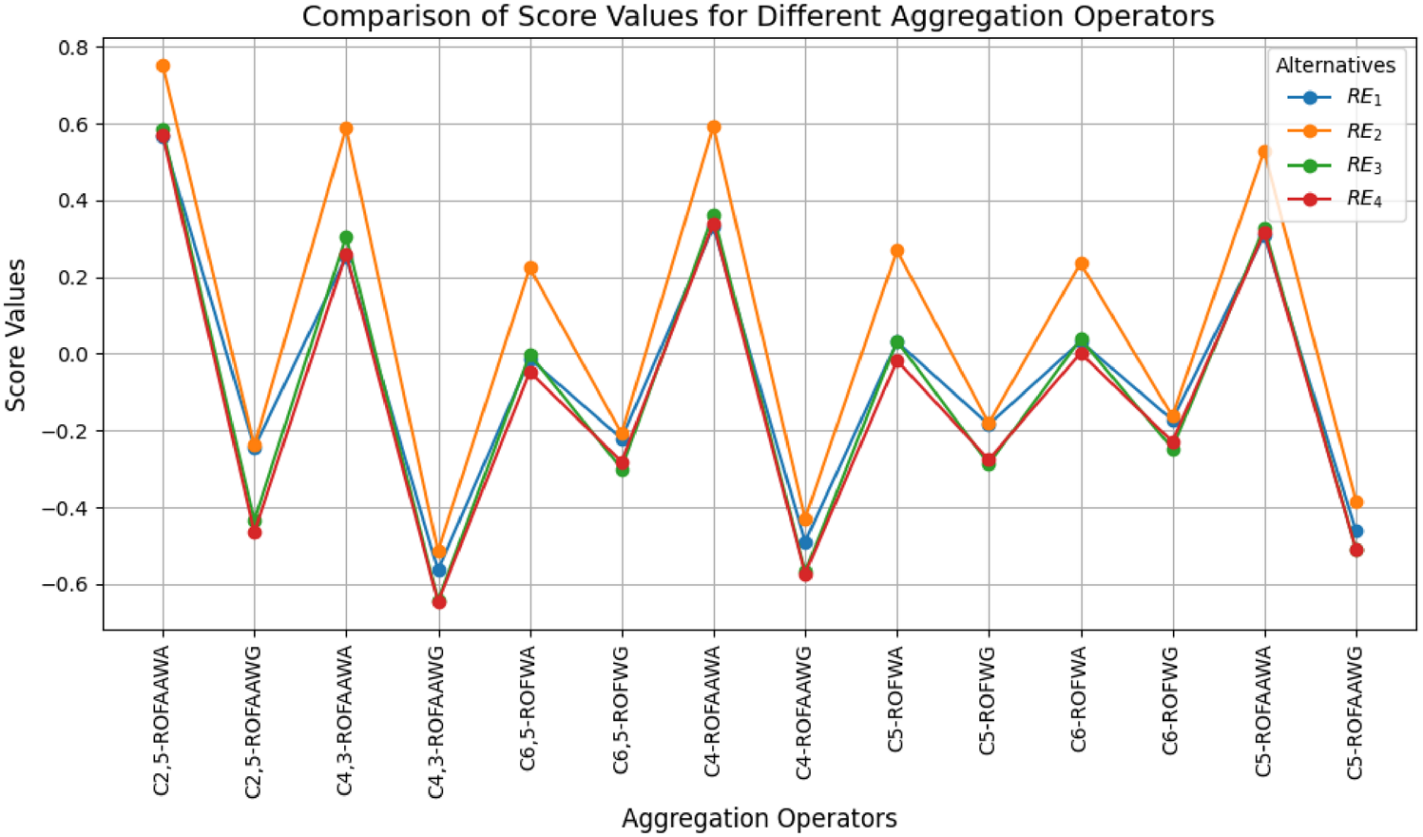
Findings from the comparison study.

Figure 5 provides a visual comparison of operator performance, clearly illustrating the superior ranking differentiation and stability of the proposed aggregation methods relative to traditional techniques. This comprehensive analysis demonstrates that our Cn,m-ROFAAWA and Cn,m-ROFAAWG operators offer a more flexible, reliable, and accurate framework for MADM applications, especially in renewable energy selection and similar complex decision-making problems.

**Table 7:** Comparison of existing AOs and our suggested techniques for $\mathscr {I}=7$.

Aggregation operators	$\dot{s}({RE}_1)$	$\dot{s}({RE}_2)$	$\dot{s}({RE}_3)$	$\dot{s}({RE}_4)$	Ranking
C2,5-ROFAAWA	0.5662	0.7525	0.5852	0.5710	${RE}_{2} \succ {RE}_{3} \succ {RE}_{4} \succ {RE}_{1}$
C2,5-ROFAAWG	– 0.2434	-0.2385	– 0.4330	– 0.4641	${RE}_{2} \succ {RE}_{1} \succ {RE}_{3} \succ {RE}_{4}$
C4,3-ROFAAWA	0.2530	0.5881	0.3063	0.2609	${RE}_{2} \succ {RE}_{3} \succ {RE}_{4} \succ {RE}_{1}$
C4,3-ROFAAWG	-0.5633	– 0.5133	– 0.6424	– 0.6462	${RE}_{2} \succ {RE}_{1} \succ {RE}_{3} \succ {RE}_{4}$
C6,5-ROFWA^24^	– 0.0130	0.2237	-0.0015	– 0.0472	${RE}_{2} \succ {RE}_{3} \succ {RE}_{1} \succ {RE}_{4}$
C6,5-ROFWG^24^	– 0.2211	-0.2077	-0.2995	– 0.2811	${RE}_{2} \succ {RE}_{1} \succ {RE}_{4} \succ {RE}_{3}$
C4,3-ROFWA^24^	-0.0631	0.2511	-0.0638	– 0.1465	${RE}_{2} \succ {RE}_{1} \succ {RE}_{3} \succ {RE}_{4}$
C4,3-ROFWG^24^	– 0.2763	-0.2745	-0.4158	-0.4237	${RE}_{2} \succ {RE}_{1} \succ {RE}_{3} \succ {RE}_{4}$
C5-ROFWA^22^	0.0311	0.2689	0.0332	-0.0174	${RE}_{2} \succ {RE}_{3} \succ {RE}_{1} \succ {RE}_{4}$
C5-ROFWG^22^	– 0.1844	-0.1803	-0.2868	-0.2758	${RE}_{2} \succ {RE}_{1} \succ {RE}_{4} \succ {RE}_{3}$
C6-ROFWA^22^	0.0320	0.2360	0.0383	0.0017	${RE}_{2} \succ {RE}_{3} \succ {RE}_{1} \succ {RE}_{4}$
C6-ROFWG^22^	– 0.1723	– 0.1624	– 0.2465	-0.2287	${RE}_{2} \succ {RE}_{1} \succ {RE}_{4} \succ {RE}_{3}$
C4-ROFAAWA^35^	0.3298	0.5914	0.3626	0.3380	${RE}_{2} \succ {RE}_{3} \succ {RE}_{4} \succ {RE}_{1}$
C4-ROFAAWG^35^	-0.4892	– 0.4290	– 0.5677	– 0.5720	${RE}_{2} \succ {RE}_{1} \succ {RE}_{3} \succ {RE}_{4}$
C5-ROFAAWA^35^	0.3080	0.5274	0.3289	0.3158	${RE}_{2} \succ {RE}_{3} \succ {RE}_{4} \succ {RE}_{1}$
C5-ROFAAWG^35^	– 0.4612	– 0.3828	– 0.5095	– 0.5100	${RE}_{2} \succ {RE}_{1} \succ {RE}_{3} \succ {RE}_{4}$
C3-ROFWA^22^	–	–	–	–	Unsuccessful
C3-ROFWG^22^	–	–	–	–	Unsuccessful
C3-ROFAAWA^35^	–	–	–	–	Unsuccessful
C3-ROFAAWG^35^	–	–	–	–	Unsuccessful
C2-ROFWA^22^	–	–	–	–	Unsuccessful
C2-ROFWG^22^	–	–	–	–	Unsuccessful
C2-ROFAAWA^35^	–	–	–	–	Unsuccessful
C2-ROFAAWG^35^	–	–	–	–	Unsuccessful
CFFWA^23^	–	–	–	–	Unsuccessful
CFFWG^23^	–	–	–	–	Unsuccessful
CFFAAWA^34^	–	–	–	–	Unsuccessful
CFFAAWG^34^	–	–	–	–	Unsuccessful
CPFWA^21^	–	–	–	–	Unsuccessful
CPFWG^21^	–	–	–	–	Unsuccessful
CPFAAWA^33^	–	–	–	–	Unsuccessful
CPFAAWG^33^	–	–	–	–	Unsuccessful
CIFWA^20^	–	–	–	–	Unsuccessful
CIFWG^20^	–	–	–	–	Unsuccessful
CIFAAWA^32^	–	–	–	–	Unsuccessful
CIFAAWG^32^	–	–	–	–	Unsuccessful

## Sensitivity analysis and limitations of the suggested operations

This section conducts a sensitivity analysis to evaluate the robustness of the proposed aggregation operators across different conditions. Furthermore, the limitations of the methodologies are critically assessed, highlighting their scope and suggesting areas for potential enhancement.

### **Sensitivity analysis of the suggested operations**

**Fig. 6:**
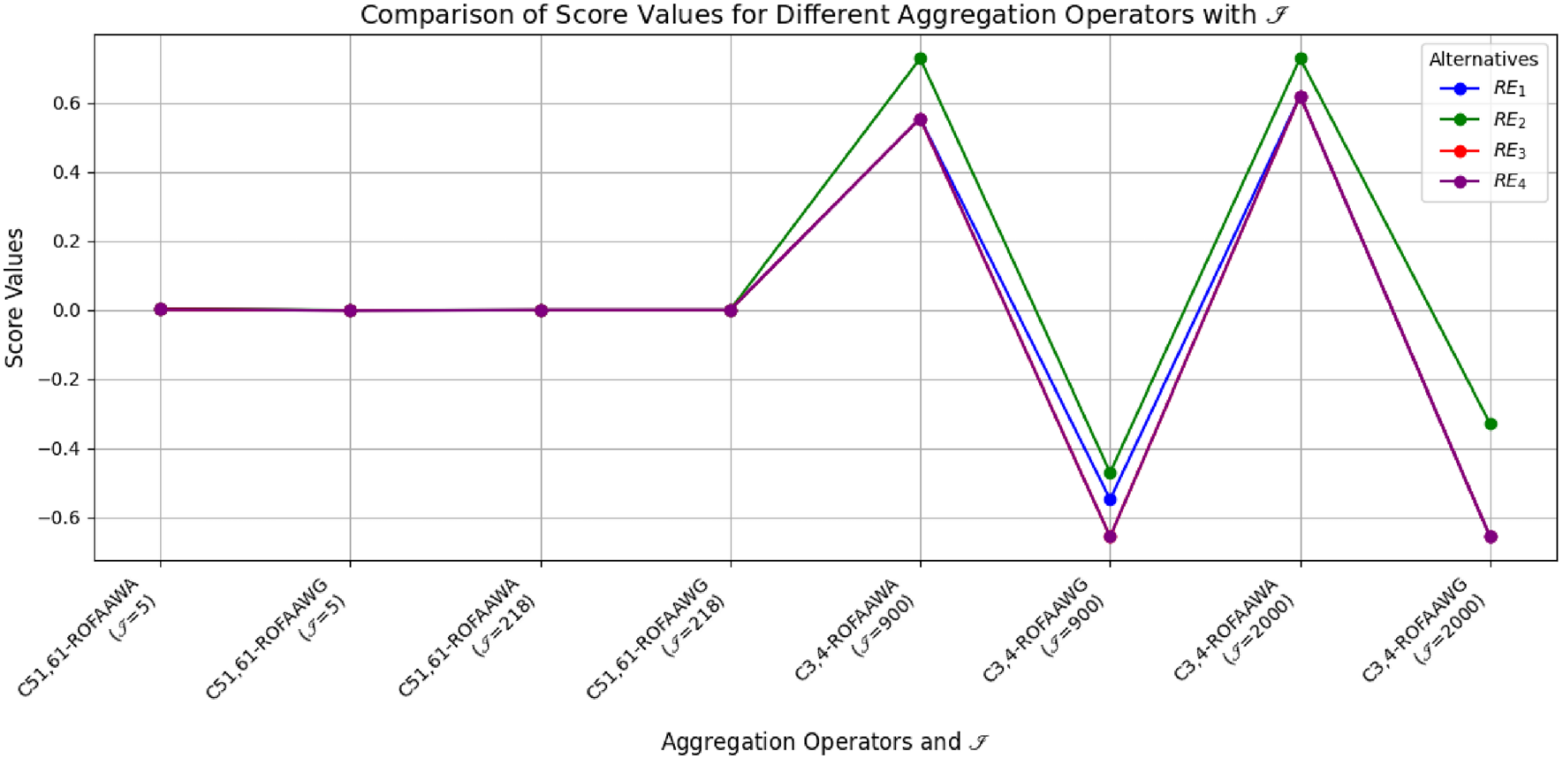
Chart visualization of data from Table 8.

By examining the effects of changing the values of $\mathscr {I}$, *n*, and *m* impacting the ranking outcomes, the sensitivity of the suggested decision support model was assessed. The findings show how parameter values affect the Cn,m-ROFAAWA and Cn,m-ROFAAWG models performance in various contexts:

1. In cases where *n* and *m* take large values while $\mathscr {I}$ is set to a small value, or conversely, when *n* and *m* are small and $\mathscr {I}$ is large, the output scores of the suggested methods tend to move toward convergence.
2. When all parameters $\mathscr {I}$, *n*, and *m* take on high values, the proposed operators generate output scores that converge to zero for all alternatives. These convergence results in nearly identical scores for the alternatives, diminishing the operators’ ability to effectively differentiate between them and yielding outcomes that lack reliability. The outcomes in this case are detailed in Table 8, with a corresponding chart visualization provided in Fig. 6.
3. When $\mathscr {I}$, *n*, and *m* are set to small values, the suggested approaches demonstrate superior effectiveness by accurately distinguishing between the alternatives. For this situation, the resulting scores effectively reveal the best option, ensuring strong and accurate rankings. The outcomes, presented in Tables 3 and 7, highlight the model’s capability in applied decision-making scenarios under small parameter settings.

### Constraints of the suggested operations

1. Influence of high parameter magnitudes: Setting the parameters $\mathscr {I}$, $n$, and $m$ to elevated levels affects the ranking sequence generated by the suggested AOs. Under these circumstances, the score outputs from the Cn,m-ROFAAWA and Cn,m-ROFAAWG functions tend to converge, leading to closely clustered scores among alternatives. This behavior, illustrated in the sensitivity analysis, restricts the models’ capacity to clearly differentiate between options.
2. Advantage of lower parameter values: For improved outcomes and maintaining clear separation in rankings, employing smaller values for $\mathscr {I}$, $n$, and $m$ is recommended. This approach enables the operators to better reflect the differences across alternatives.
3. Practical limitations concerning high parameter values: Large values for $\mathscr {I}$, $n$, and $m$ are uncommon in real-life applications. Hence, these situations can generally be excluded in favor of more realistic conditions where parameters assume smaller values.

**Table 8:** Sensitivity assessment of the parameters $\mathscr {I}$, *n*, and *m* in the case study of Section 5.2.

$\mathscr {I}$	Operators	$\dot{s}({RE}_1)$	$\dot{s}({RE}_2)$	$\dot{s}({RE}_3)$	$\dot{s}({RE}_4)$	Ranking and Ordering
5	C51,61-ROFAAWA	0.0017	0.0036	0.0017	0.0017	${RE}_{2} \succ {RE}_{1} \approx {RE}_{3} \approx {RE}_{4}$
5	C51,61-ROFAAWG	-0.0012	-0.0007	-0.0012	-0.0012	${RE}_{2} \succ {RE}_{1} \approx {RE}_{3} \approx {RE}_{4}$
218	C51,61-ROFAAWA	0.0000	0.0000	0.0000	0.0000	${RE}_{2} \approx {RE}_{1} \approx {RE}_{3} \approx {RE}_{4}$
218	C51,61-ROFAAWG	0.0000	0.0000	0.0000	0.0000	${RE}_{2} \approx {RE}_{1} \approx {RE}_{3} \approx {RE}_{4}$
900	C3,4-ROFAAWA	0.5548	0.7285	0.5549	0.5548	${RE}_{2} \succ {RE}_{3} \succ {RE}_{1} \approx {RE}_{4}$
900	C3,4-ROFAAWG	-0.5474	-0.4697	-0.6555	-0.6555	${RE}_{2} \succ {RE}_{1} \succ {RE}_{3} \approx {RE}_{4}$
2000	C3,4-ROFAAWA	0.6202	0.7288	0.6202	0.6202	${RE}_{2} \succ {RE}_{1} \approx {RE}_{3} \approx {RE}_{4}$
2000	C3,4-ROFAAWG	-0.6558	-0.3280	-0.6558	-0.6558	${RE}_{2} \succ {RE}_{1} \approx {RE}_{3} \approx {RE}_{4}$

## Conclusions

This study introduced a robust decision-making framework based on complex n,m-rung orthopair fuzzy sets and aczel-alsina aggregation operations, aimed at enhancing accuracy and flexibility in multiple attribute decision-making under uncertainty and ambiguity. Two novel aggregation operators, Cn,m-ROFAAWA and Cn,m-ROFAAWG, were developed and analyzed for key theoretical properties including boundedness, idempotency, and monotonicity. The proposed framework was applied to a renewable energy selection problem. The numerical simulations demonstrated that Wind Energy (${RE}_2$) consistently emerged as the top-ranked option, regardless of the operator used or the variations in parameter values, highlighting the reliability and robustness of the suggested methods. Comparative analysis with existing aggregation approaches confirmed that the proposed operators provide superior differentiation among alternatives and more precise decision-making outcomes. Parameter impact analysis revealed that increasing $\mathscr {I}$ generally raises the score values of all alternatives, while the optimal choice remains unchanged, confirming the isotonicity of the proposed operators. Sensitivity analysis further showed that high parameter magnitudes ($\mathscr {I}, n, m$) can lead to convergence of output scores, reducing the operators’ discrimination ability, whereas smaller parameter values allow clear and accurate differentiation between alternatives, making the operators practical for real-world applications. In summary, the integration of n,m-rung orthopair fuzziness with aczel-alsina operations provides enhanced flexibility, reliability, and accuracy in MADM. The proposed aggregation operators are both theoretically sound and practically effective, offering robust tools for decision-making in renewable energy and other complex domains.

Future research will focus on applying the framework to dynamic or large-scale decision-making problems, refining the aggregation operators to enhance efficiency and robustness, and exploring integration with other fuzzy information frameworks, such as $k^{n}_{m}$-rung picture fuzzy information^36^ or bipolar n,m-ROF sets^37^, to further expand the framework’s applicability and flexibility.

## Data Availability

All data generated or analysed during this study are included in this published article.

## Abbreviations

Cn,m-ROFS: Complex n,m-rung orthopair fuzzy set
Cn,m-ROFNs: Complex n,m-rung orthopair fuzzy numbers
Cn,m-ROFAAWA: Complex n,m-ROF aczel-alsina weighted average
Cn,m-ROFAAWG: Complex n,m-ROF aczel-alsina weighted geometric
Cn,m-ROFWA: Complex n,m-ROF weighted average
Cn,m-ROFWG: Complex n,m-ROF weighted geometric
Cq-ROF: Complex q-rung orthopair fuzzy
Cq-ROFWA: Complex q-ROF weighted average
Cq-ROFWG: Complex q-ROF weighted geometric
Cq-ROFAAWA: Complex q-ROF aczel-alsina weighted average
Cq-ROFAAWG: Complex q-ROF aczel-alsina weighted geometric
CFF: Complex Fermatean fuzzy
CFFWA: Complex Fermatean fuzzy weighted average
CFFWG: Complex Fermatean fuzzy weighted geometric
CFFAAWA: Complex Fermatean fuzzy aczel-alsina weighted average
CFFAAWG: Complex Fermatean fuzzy aczel-alsina weighted geometric
CPyF: Complex Pythagorean fuzzy
CPyFWA: Complex Pythagorean fuzzy weighted average
CPyFWG: Complex Pythagorean fuzzy weighted geometric
CPFAAWA: Complex Pythagorean fuzzy aczel-alsina weighted average
CPFAAWG: Complex Pythagorean fuzzy aczel-alsina weighted geometric
CIF: Complex intuitionistic fuzzy
CIFWA: Complex intuitionistic fuzzy weighted average
CIFWG: Complex intuitionistic fuzzy weighted geometric
CIFAAWA: Complex intuitionistic fuzzy aczel-alsina weighted average
CIFAAWG: Complex intuitionistic fuzzy aczel-alsina weighted geometric
MADM: Multiple attribute decision-making
AO: Aggregation operator
$\mathscr {I}$: Parameter in aczel-alsina operations
$\mathscr {S}$: General Cn,m-ROFN element
RE: Renewable energy option

## References

[1] Zadeh, L. A. Fuzzy sets. *Inf. Control* **8**(3), 338–353 (1965).

[2] Özlü, Ş & Karaaslan, F. Correlation coefficient of T-spherical type-2 hesitant fuzzy sets and their applications in clustering analysis. *J Ambient Intell Human Comput.* **13**, 329–357. 10.1007/s12652-021-02904-8 (2022).

[3] Özlü, Ş. Multi-criteria decision making based on vector similarity measures of picture type-2 hesitant fuzzy sets. *Granular Comput.* **8**, 1505–1531. 10.1007/s41066-023-00382-1 (2023).

[4] Özlü, Ş. Generalized dice measures of single valued neutrosophic type-2 hesitant fuzzy sets and their application to multi-criteria decision making problems. *Int. J. Mach. Learn. Cybern.* **14**, 33–62. 10.1007/s13042-021-01480-9 (2023).

[5] Alqahtani, M. H., Lou, D.-C., Sikander, F., Saber, Y. & Lee, C.-C. Novel fuzzy ostrowski integral inequalities for convex fuzzy-valued mappings over a harmonic convex set: Extending real-valued intervals without the sugeno integrals. *Mathematics* **12**, 3495. 10.3390/math12223495 (2024).

[6] Musa, S. Y., Asaad, B. A., Alohali, H., Ameen, Z. A. & Alqahtani, M. H. Fuzzy N-bipolar soft sets for multi-criteria decision-making: Theory and application. *Comput. Model. Eng. Sci.* **143**(1), 911–943. 10.32604/cmes.2025.062524 (2025).

[7] Atanassov, K. T. Intuitionistic fuzzy sets. *Fuzzy Sets Syst.* **20**(1), 87–96 (1986).

[8] Yager, R. R. Pythagorean fuzzy subsets. *in Proceedings of the 2013 Joint IFSA World Congress and NAFIPS Annual Meeting (IFSA/NAFIPS), IEEE, Edmonton, AB, Canada*, (2013), 57-61.

[9] Yager, R. R. Generalized orthopair fuzzy sets. *IEEE Trans. Fuzzy Syst.* **25**(5), 1222–1230 (2016).

[10] Senapati, T. & Yager, R. R. Fermatean fuzzy sets. *J. Ambient. Intell. Humaniz. Comput.* **11**, 663–674 (2020).

[11] Fahmi, A. et al. Triangular intuitionistic fuzzy frank aggregation for efficient renewable energy project selection. *Eur. J. Pure Appl. Math.* **18**(3), 6227. 10.29020/nybg.ejpam.v18i3.6227 (2025).

[12] Fahmi, A. et al. Circular intuitionistic fuzzy Hamacher aggregation operators for multi-attribute decision-making. *Sci. Rep.* **15**, 5618. 10.1038/s41598-025-88845-0 (2025). 39955333 10.1038/s41598-025-88845-0 PMC11830057

[13] Fahmi, A., Khan, A. & Abdeljawad, T. Group decision making based on cubic fermatean Einstein fuzzy weighted geometric operator. *Ain Shams Eng. J.* **15**(4), 102737. 10.1016/j.asej.2024.102737 (2024).

[14] Özlü, Ş & Aktaş, H. Correlation coefficient of r, s, t-spherical hesitant fuzzy sets and MCDM problems based on clustering algorithm and technique for order preference by similarity to ideal solution method. *Comput. Appl. Math.* **43**, 429. 10.1007/s40314-024-02942-w (2024).

[15] Ibrahim, H. Z. & Alshammari, I. n, m-rung orthopair fuzzy sets with applications to multicriteria decision making. *IEEE Access* **10**, 99562–99572 (2022).

[16] Ramot, D., Milo, R., Fiedman, M. & Kandel, A. Complex fuzzy sets. *IEEE Trans. Fuzzy Syst.* **10**(2), 171–186 (2002).

[17] Fahmi, A. et al. Analyzing global economic shifts due to the afghan-america war using complex cubic fuzzy TODIM method. *Eur. J. Pure Appl. Math.* **18**(3), 5866. 10.29020/nybg.ejpam.v18i3.5866 (2025).

[18] Özlü, Ş. Bipolar-valued complex hesitant fuzzy Dombi aggregating operators based on multi-criteria decision-making problems. *Int. J. Fuzzy Syst.* **27**, 162–189. 10.1007/s40815-024-01770-8 (2025).

[19] Fahmi, A., Khan, A. & Abdeljawad, T. A novel approach to group decision-making using generalized bipolar neutrosophic sets. *PLoS ONE* **20**(6), e0317746. 10.1371/journal.pone.0317746 (2025). 40498762 10.1371/journal.pone.0317746 PMC12157272

[20] Alkouri, A., Salleh, A. Complex intuitionistic fuzzy sets. *In: 2nd International Conference on Fundamental and Applied Sciences*, 464-47, (2012).

[21] Ullah, K., Mahmood, T., Ali, Z. & Jan, N. On some distance measures of complex Pythagorean fuzzy sets and their applications in pattern recognition. *Complex Intell. Syst.* **6**, 15–27. 10.1007/s40747-019-0103-6 (2020).

[22] Liu, P., Mahmood, T. & Ali, Z. Complex q-rung orthopair fuzzy aggregation operators and their applications in multi-attribute group decision making. *Information* **11**(1), 5. 10.3390/info11010005 (2020).

[23] Chinnadurai, V., Thayalan, S. & Bobin, A. Multi-criteria decision-making in complex Fermatean fuzzy environment. *J. Math. Comput. Sci.* **11**, 7209–7227 (2021).

[24] Ibrahim, H. Z. Exploring complex n, m-rung orthopair fuzzy aggregation operators for enhanced multi-attribute decision making. *Granul. Comput.*10.1007/s41066-024-00471-9 (2024).

[25] Aczél, J. & Alsina, C. Characterizations of some classes of quasilinear functions with applications to triangular norms and to synthesizing judgements. *Aeq. Math.* **25**, 313–315. 10.1007/BF02189626 (1982).

[26] Senapati, T., Chen, G. & Yager, R. R. Aczel-Alsina aggregation operators and their application to intuitionistic fuzzy multiple attribute decision making. *Int. J. Intell. Syst.* **37**(2), 1529–1551. 10.1002/int.22684 (2022).

[27] Hussain, A., Ullah, K., Alshahrani, M. N., Yang, M.-S. & Pamucar, D. Novel Aczel-Alsina operators for pythagorean fuzzy sets with application in multi-attribute decision making. *Symmetry* **14**, 940. 10.3390/sym14050940 (2022).

[28] Haq, I. U. et al. Novel Fermatean Fuzzy Aczel-Alsina model for investment strategy selection. *Mathematics* **11**, 3211. 10.3390/math11143211 (2023).

[29] Farid, H. M. A. & Riaz, M. q-rung orthopair fuzzy aczel–alsina aggregation operators with multi-criteria decision-making. *Eng. Appl. Artif. Intell.* **122**, 106105. 10.1016/j.engappai.2023.106105 (2023).

[30] Özlü, Ş. New q-rung orthopair fuzzy Aczel-Alsina weighted geometric operators under group-based generalized parameters in multi-criteria decision-making problems. *Comput. Appl. Math.* **43**, 122. 10.1007/s40314-024-02646-1 (2024).

[31] Ali, J. & Naeem, M. Analysis and application of p, q-Quasirung Orthopair Fuzzy Aczel-Alsina aggregation operators in multiple criteria decision-making. *IEEE Access* **2023**(11), 49081–49101. 10.1109/ACCESS.2023.3274494 (2023).

[32] Mahmood, T., Ali, Z., Baupradist, S. & Chinram, R. Complex intuitionistic Fuzzy Aczél-Alsina aggregation operators and their application in multi-attribute decision-making. *Symmetry* **14**, 2255. 10.3390/sym14112255 (2022).

[33] Jin, H., Hussain, A., Ullah, K. & Javed, A. Novel complex pythagorean fuzzy sets under Aczel-Alsina operators and their application in multi-attribute decision making. *Symmetry* **15**(1), 68. 10.3390/sym15010068 (2023).

[34] Chen, L., Zhou, X., Wu, M., Shi, Y. & Wang, Y. Aczél-Alsina aggregation operators on complex fermatean fuzzy information with application to multi-attribute decision making. *IEEE Access* **11**, 141703–141722. 10.1109/ACCESS.2023.3342175 (2023).

[35] Ali, J. & Naeem, M. Complex q-Rung Orthopair Fuzzy Aczel-Alsina aggregation operators and its application to multiple criteria decision-making with unknown weight information. *Access* **2022**(10), 85315–85342. 10.1109/ACCESS.2022.3197597 (2022).

[36] Ibrahim, H. Z. et al. -Rung picture fuzzy information in a modern approach to multi-attribute group decision-making. *Complex Intell. Syst.* **10**, 2605–2625. 10.1007/s40747-023-01277-z (2024).

[37] Ibrahim, H. Z. Multi-attribute group decision-making based on bipolar n, m-rung orthopair fuzzy sets. *Granul. Comput.* **8**, 1819–1836. 10.1007/s41066-023-00405-x (2023).

